# Characterization and Compensation of Network-Level Anomalies in Mixed-Signal Neuromorphic Modeling Platforms

**DOI:** 10.1371/journal.pone.0108590

**Published:** 2014-10-10

**Authors:** Mihai A. Petrovici, Bernhard Vogginger, Paul Müller, Oliver Breitwieser, Mikael Lundqvist, Lyle Muller, Matthias Ehrlich, Alain Destexhe, Anders Lansner, René Schüffny, Johannes Schemmel, Karlheinz Meier

**Affiliations:** 1 Ruprecht-Karls-Universität Heidelberg, Kirchhoff Institute for Physics, Heidelberg, Germany; 2 Technische Universität Dresden, Institute of Circuits and Systems, Dresden, Germany; 3 Department of Computational Biology, School of Computer Science and Communication, Stockholm University and Royal Institute of Technology, Stockholm, Sweden; 4 CNRS, Unité de Neuroscience, Information et Complexité, Gif sur Yvette, France; Georgia State University, United States of America

## Abstract

Advancing the size and complexity of neural network models leads to an ever increasing demand for computational resources for their simulation. Neuromorphic devices offer a number of advantages over conventional computing architectures, such as high emulation speed or low power consumption, but this usually comes at the price of reduced configurability and precision. In this article, we investigate the consequences of several such factors that are common to neuromorphic devices, more specifically limited hardware resources, limited parameter configurability and parameter variations due to fixed-pattern noise and trial-to-trial variability. Our final aim is to provide an array of methods for coping with such inevitable distortion mechanisms. As a platform for testing our proposed strategies, we use an executable system specification (ESS) of the BrainScaleS neuromorphic system, which has been designed as a universal emulation back-end for neuroscientific modeling. We address the most essential limitations of this device in detail and study their effects on three prototypical benchmark network models within a well-defined, systematic workflow. For each network model, we start by defining quantifiable functionality measures by which we then assess the effects of typical hardware-specific distortion mechanisms, both in idealized software simulations and on the ESS. For those effects that cause unacceptable deviations from the original network dynamics, we suggest generic compensation mechanisms and demonstrate their effectiveness. Both the suggested workflow and the investigated compensation mechanisms are largely back-end independent and do not require additional hardware configurability beyond the one required to emulate the benchmark networks in the first place. We hereby provide a generic methodological environment for configurable neuromorphic devices that are targeted at emulating large-scale, functional neural networks.

## Introduction

### 1.1 Modeling and Computational Neuroscience

The limited availability of detailed biological data has always posed a major challenge to the advance of neuroscientific understanding. The formulation of theories about information processing in the brain has therefore been predominantly model-driven, with much freedom of choice in model architecture and parameters. As more powerful mathematical and computational tools became available, increasingly detailed and complex cortical models have been proposed. However, because of the manifest nonlinearity and sheer complexity of interactions that take place in the nervous system, analytically treatable ensemble-based models can only partly cover the vast range of activity patterns and behavioral phenomena that are characteristic for biological nervous systems [1]. The high level of model complexity often required for computational proficiency and biological plausibility has led to a rapid development of the field of computational neuroscience, which focuses on the simulation of network models as a powerful complement to the search for analytic solutions [2].

The feasibility of the computational approach has been facilitated by the development of the hardware devices used to run neural network simulations. The brisk pace at which available processing speed has been increasing over the past few decades, as allegorized by Moore's Law, as well as the advancement of computer architectures in general, closely correlate to the size and complexity of simulated models. Today, network models with tens of thousands of neurons are routinely simulated on desktop machines, with supercomputers allowing several orders of magnitude more [3], [4]. However, as many authors have pointed out (see e.g. [5], [2]), the inherently massively parallel structure of biological neural networks becomes progressively difficult to map to conventional architectures based on digital general-purpose CPUs, as network size and complexity increase.

Conventional simulation becomes especially restrictive when considering long time scales, such as are required for modeling long-term network dynamics or when performing statistics-intensive experiments. Additionally, power consumption can quickly become prohibitive at these scales [6], [7].

### 1.2 Neuromorphic Hardware

The above issues can, however, be eluded by reconsidering the fundamental design principles of conventional computer systems. The core idea of the so-called neuromorphic approach is to implement features (such as connectivity) or components (neurons, synapses) of neural networks directly *in silico*: instead of calculating the dynamics of neural networks, neuromorphic devices contain physical representations of the networks themselves, behaving, by design, according to the same dynamic laws. An immediate advantage of this approach is its inherent parallelism (emulated network components evolve in parallel, without needing to wait for clock signals or synchronization), which is particularly advantageous in terms of scalability. First proposed by Mead in the 1980s [8]–[10], the neuromorphic approach has since delivered a multitude of successful applications [11]–[14].

By far the largest number of neuromorphic systems developed thus far are highly application-specific, such as visual processing systems [15]–[18] or robotic motor control devices [19]. Several groups have focused on more biological aspects, such as the neuromorphic implementation of biologically-inspired self-organization and learning [20], [21], detailed replication of Hodgkin-Huxley neurons [22] or hybrid systems interfacing analog neural networks with living neural tissue [23].

These devices, however, being rather specialized, can not match the flexibility of traditional software simulations. Adding configurability comes at a high price in terms of hardware resources, due to various hardware-specific limitations, such as physical size and essentially two-dimensional structure. So far there have only been few attempts at realizing highly configurable hardware emulators [24]–[28]. This approach alone, however, does not completely resolve the computational bottleneck of software simulators, as scaling neuromorphic neural networks up in size becomes non-trivial when considering bandwidth limitations between multiple interconnected hardware devices [29]–[33].

### 1.3 The BrainScaleS Hardware System

A very efficient way of interconnecting multiple VLSI (Very Large Scale Integration) modules is offered by so-called wafer-scale integration. This implies the realization of both the modules in question and their communication infrastructure on the same silicon wafer, the latter being done in a separate, post-processing step. The BrainScaleS wafer-scale hardware [27] uses this process to achieve a high communication bandwidth between individual neuromorphic cores on a wafer, thereby allowing a highly flexible connection topology of the emulated network. Together with the large available parameter space for neurons and synapses, this creates a neuromorphic architecture that is comparable in flexibility with standard simulation software. At the same time, it provides a powerful alternative to software simulators by avoiding the abovementioned computational bottleneck, in particular owing to the fact that the emulation duration does not scale with the size of the emulated network, since individual netowrk components operate, inherently, in parallel. An additional benefit which is inherent to this specific VLSI implementation is the high acceleration with respect to biological real-time, which is facilitated by the high on-wafer bandwidth. This allows investigating the evolution of network dynamics over long periods of time which would otherwise be strongly prohibitive for software simulations.

### 1.4 Hardware-Induced Distortions: A Systematic Investigation

Along with the many advantages it offers, the neuromorphic approach also comes with limitations of its own. These have various causes that lie both in the hardware itself and the control software. We will later identify these causes, which we henceforth refer to as *distortion mechanisms*. The neural network emulated by the hardware device can therefore differ significantly from the original model, be it in terms of pulse transmission, connectivity between populations or individual neuron or synapse parameters. We refer to all the changes in network dynamics (i.e., deviations from the original behavior defined by software simulations) caused by hardware-specific effects as *hardware-induced distortions*.

Due to the complexity of state-of-the-art neuromorphic platforms and their control software, as well as the vast landscape of emulable neural network models, a thorough and systematic approach is essential for providing reliable information about causal mechanisms and functional effects of hardware-induced distortions in model dynamics and for ultimately designing effective compensation methods. In this article, we design and perform such a systematic analysis and compensation for several hardware-specific distortion mechanisms.

First and foremost, we identify and quantify the most important sources of model distortions. We then proceed to investigate their effect on network functionality. In order to cover a wide range of possible network dynamics, we have chosen three very different cortical network models to serve as benchmarks. In particular, these models implement several prototypical cortical paradigms of computation, relying on winner-take-all structures (attractor networks), precise spike timing correlations (synfire chains) or balanced activity (self-sustained asynchronous irregular states).

For every emulated model, we define a set of functionality criteria, based on specific aspects of the network dynamics. This set should be complex enough to capture the characteristic network behavior, from a microscopic (e.g., membrane potentials) to a mesoscopic level (e.g., firing rates) and, where suitable, computational performance at a specific task. Most importantly, these criteria need to be precisely quantified, in order to facilitate an accurate comparison between software simulations and hardware emulations or between different simulation/emulation back-ends in general. The chosen functionality criteria should also be measured, if applicable, for various relevant realizations (i.e. for different network sizes, numbers of functional units etc.) of the considered network.

Because multiple distortion mechanisms occur simultaneously in hardware emulations, it is often difficult, if not impossible, to understand the relationship between the observed effects (i.e., modifications in the network dynamics) and their potential underlying causes. Therefore, we investigate the effects of individual distortion mechanisms by implementing them, separately, in software simulations. As before, we perform these analyses over a wide range of network realizations, since - as we will show later - these may strongly influence the effects of the examined mechanisms.

After having established the relationship between structural distortions caused by hardware-specific factors and their consequences for network dynamics, we demonstrate various compensation techniques in order to restore the original network behavior.

In the final stage, for each of the studied models, we simulate an implementation on the hardware back-end by running an appropriately configured executable system specification, which includes the full panoply of hardware-specific distortion mechanisms. Using the proposed compensation techniques, we then attempt to deal with all these effects simultaneously. The results from these experiments are then compared to results from software simulations, thus allowing a comprehensive assertion of the effectivity of our proposed compensation techniques, as well as of the capabilities and limitations of the neuromorphic emulation device.

### 1.5 Article Structure

In section Neuromorphic testbench and investigated distortion mechanisms, we describe our testbench neuromorphic modeling platform with its most relevant components, as well as the essential layers of the operation workflow. We continue by explaining the causes of various network-level distortions that are expected to be common for similar mixed-signal neuromorphic devices. In the same section, we also introduce the executable system specification of the hardware, which we later use for experimental investigations.

Section Hardware-induced distortions and compensation strategies contains the description of the three benchmark models. We start the section on each of the models with a short summary of all the relevant findings. We then describe its architecture and characteristic aspects of its dynamics which we later use as quality controls. We continue by discussing the effects of individual hardware-specific distortion mechanisms as observed in software simulations, propose various compensation strategies and investigate their efficacy in restoring the functionality of the network model in question. Subsequently, we apply these methods to large-scale neuromorphic emulations and examine the results.

Finally, we summarize and discuss our findings in the Conclusions section.

## Neuromorphic Testbench and Investigated Distortion Mechanisms

In this section we introduce the BrainScaleS neuromorphic wafer-scale hardware system and its executable system specification, henceforth called the ESS, as the testbench for our studies. The system's hardware and software components are only described on an abstract level, while highlighting the mechanisms responsible for distortions of the emulated networks. Finally, we identify the three most relevant causes of distortion as being synapse loss, synaptic weight noise and non-configurable axonal delays.

### 2.1 The BrainScaleS Wafer-Scale Hardware

**Figure 1:**
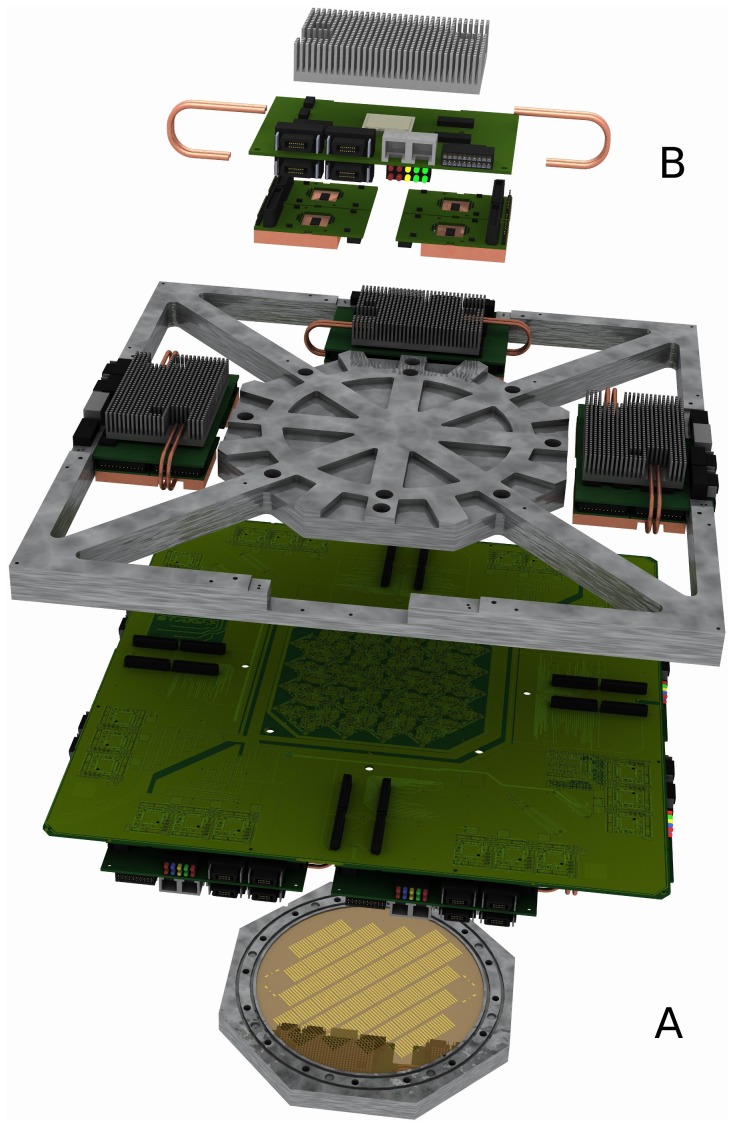
The BrainScaleS wafer-scale hardware system. (A) Wafer comprising HICANN building blocks and on-wafer communication infrastructure covered by an aluminium plate, (B) digital inter-wafer and wafer-host communication modules. Also visible: mechanical and electrical support.

**Figure 2:**
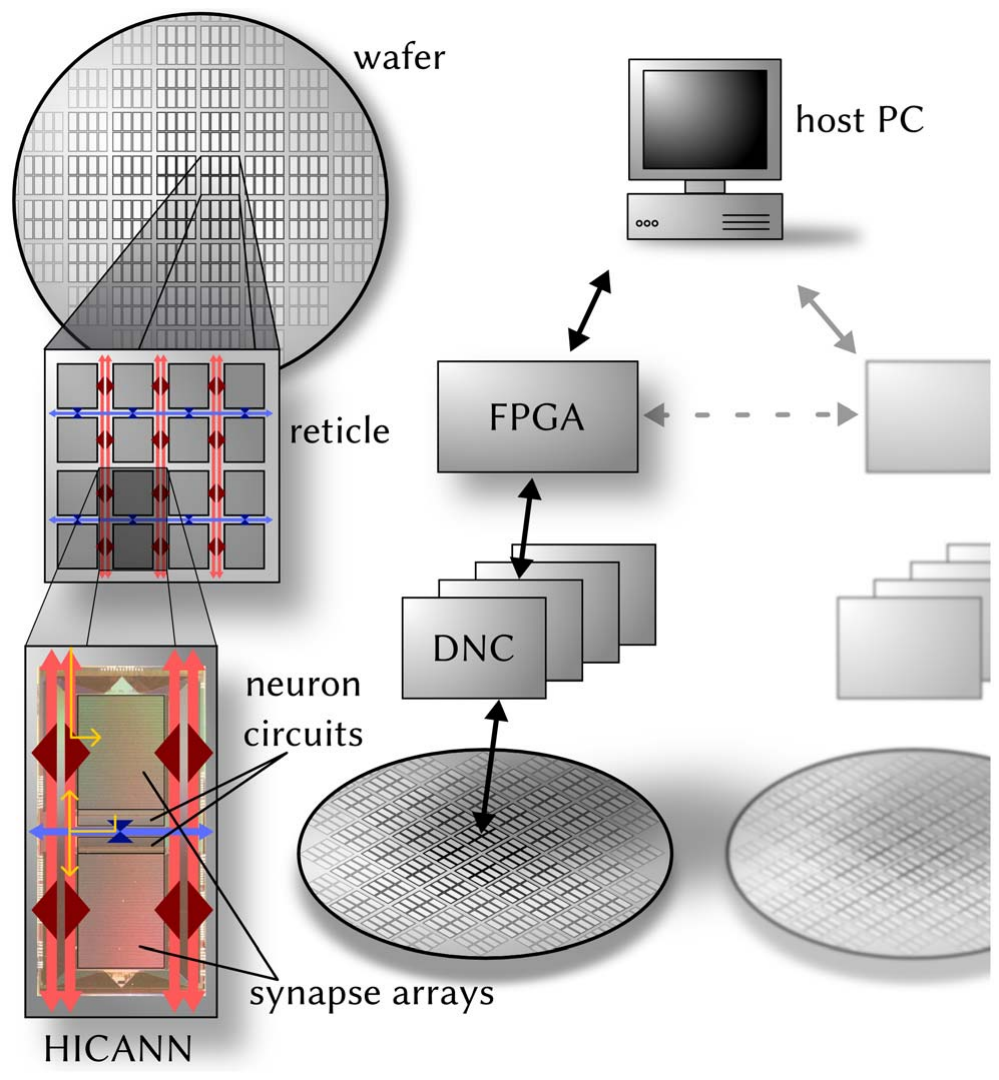
Architecture of the BrainScaleS wafer-scale hardware system. Left: The HICANN building block has two symmetric halves with synapse arrays and neuron circuits. Neural activity is transported horizontally (blue) and vertically (red) via asynchronous buses that span over the entire wafer. Exemplary spike paths are shown in yellow on the HICANN: The incoming spike packet is routed to the synapse drivers. In the event that a neuron spikes, it emits a spike packet back into the routing network. Right: Off-wafer connectivity is established by a hierarchical packed-based network via DNCs and FPGAs. It interfaces the on-wafer routing buses on the HICANN building blocks. Several wafer modules can be interconnected using routing functionality between the FPGAs.

Figure 1 shows a 3D-rendered image of the BrainScaleS wafer-scale hardware system: the 8 inch silicon wafer contains 196 608 neurons and 44 million plastic synapses implemented in mixed-signal VLSI circuitry. Due to the high integration of the circuits, the capacitances and thus the intrinsic time constants are small, so that neural dynamics take place approximately 10 000 faster than biological real time. The principal building block of the wafer is the so-called HICANN (High Input Count Analog Neural Network) chip [27], [34]. During chip fabrication one is limited to a maximum area that can be simultaneously exposed during photolitography, a reticle, thus usually such a wafer is cut into individual chips after production. For the BrainScaleS system, however, the wafer is left intact, and additional wiring is applied onto the wafer's surface in a post-processing step. This process establishes connections betwen all 384 HICANN blocks that allow a very high bandwidth for on-wafer pulse-event communication [34]. The neuromorphic wafer is accompanied by a stack of digital communication modules for the connection of the wafer to the host PC and to other wafers (Figure 2 and section 2.1.2).

#### 2.1.1 HICANN building block

**Figure 3:**
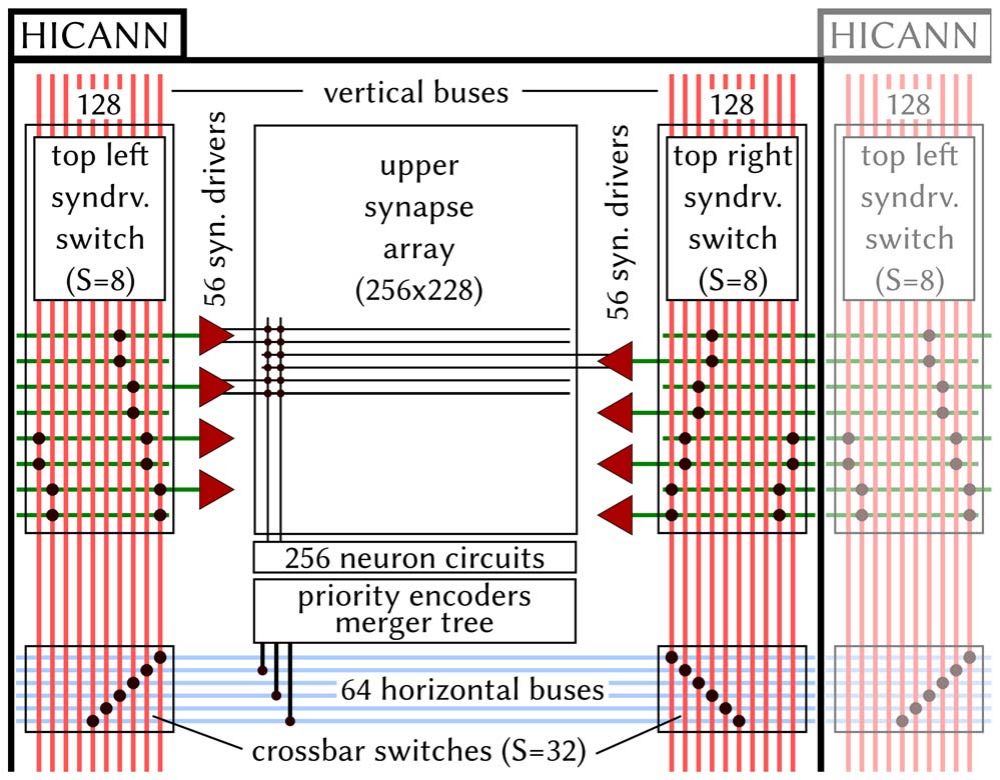
Components and connectivity of the HICANN building block. The figure shows the upper block of the HICANN chip: most of the area is occupied by the synapse array with 256 columns and 224 rows. Each synapse column is connected to one of 256 neuron circuits, from which up to 64 can be interconnected to form larger neurons with up to 14336 input synapses. When a neuron fires, a 6-bit address representing this neuron is generated and injected into one of eight accessible horizontal buses after passing a merger stage. Via two statically configurable switches (crossbar rsp. synapse driver switch) these pulses are routed to the synapse drivers, which operate two synapse rows each. Every synapse is configured to a specific 6-bit address, so that, when a pre-synaptic pulse with matching address arrives, a post-synaptic conductance course is generated at the associated neuron circuit. Both switch matrices are sparse, i.e. configurable switches do not exist at all crossings of horizontal and vertical lines, but e.g. only at every 8th crossing (Sparseness S = 8). On the wafer, the horizontal and vertical buses, as well as the horizontal lines connected to the synapse drivers do not end at the HICANN borders, but go beyond them.

On the HICANN chip (lower left of Figure 2), one can recognize two symmetric blocks which hold the analog core modules. The upper block is depicted in detail in Figure 3: Most of the area is occupied by the synapse array with 224 rows and 256 columns. All synapses in a column are connected to one of the 256 neuron circuits located at the center of the chip. For each two adjacent synapse rows, there is one *synapse driver* that forms the input for pre-synaptic pulses to the synapse array. Synapse drivers are evenly distributed to the left and right side of one synapse array (56 per side). A grid of horizontal and vertical buses enables the routing of spikes from neuron circuits to synapse drivers.

Up to 64 neuron circuits can be interconnected to form neurons with up to 14336 synapses. The neurons emulate the dynamics of the Adaptive-Exponential Integrate-and-Fire model (AdEx) [35] in analog circuitry, defined by equations for the membrane voltage *V*, the adaption current 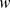 and a reset condition that applies when a spike is triggered:
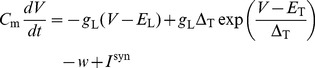
(1)

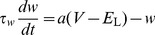
(2)

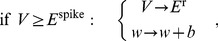
(3)where 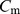, 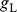 and 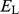 denote the membrane capacitance, leak conductance and leak potential, respectively, 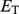 and 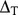 represent the spike initiation threshold and the threshold slope factor and 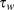 and 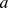 represent the adaptation time constant and coupling parameter. When 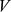 reaches a certain threshold value 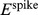, a spike is emitted and the membrane potential is reset to 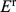. At the same time, the adaptation variable is increased by a fixed amount 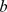, thereby allowing for spike-frequency adaptation. An absolute refractory mechanism is supported by clamping 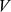 to its reset value for the refractory time 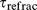. The generated spikes are transmitted digitally to synapse drivers (analog multiplier), synapses (digital multiplier) and finally other neurons, where postsynaptic conductance courses are generated and summed up linearly, resulting in the synaptic current 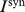:



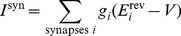
(4)

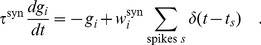
(5)


Here, 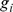 represents the synaptic conductance and 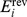 the synaptic reversal potential of the 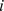-th synapse, 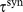 the time constant of the exponential decay and 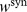 the synaptic weight. In the hardware implementation [36], each neuron features two of such synaptic input circuits, which are typically used for excitatory and inhibitory input. Nearly all parameters of the neuron model and the synaptic input circuits are individually adjustable by means of analog storage banks based on floating gate technology [37]. In the hardware neuron, both the circuit for the adaption mechanism and the exponential term circuit can be effectively disconnected from the membrane capacitance, such that a simple Leaky Integrate-and-Fire (LIF) model can also be emulated. The hardware membrane capacitance is fixed to one of two possible values. As the parameters controlling the temporal dynamics of the neuron such as 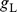 and the time constants are configurable within a wide range, the hardware is able to run at a variable speedup factor (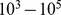) compared to biological real time. In particular, the translation of the membrane capacitance between the hardware and the biological domain can be chosen freely due to the independent configurability of both membrane and synaptic conductances, thereby effectively allowing the emulation of point neurons of arbitrary size - within the limits imposed by the hardware parameter ranges.

In contrast to neurons, where each parameter is fully configurable within the specified ranges, the *synaptic weights* are adjustable by a combination of analog and digital memories. The synaptic weight 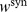 is proportional to a row-wise adjustable analog parameter 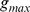 and to a 4-bit digital weight specific to each synapse. The 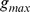 of two adjacent rows can be configured to be a fixed multiple of each other. This way, two synapses of adjacent rows can be combined to offer a weight resolution of 8 bits, at the cost of halving the number of synapses for this synapse driver.

*Long-term learning* is incorporated in every synapse through spike-timing-dependent plasticity (STDP) [38]. The implemented STDP mechanism follows a pairwise update rule with programmable update functions [39]. As STDP is not contained in the models investigated in this article, we refer to [40]–[42] for details on the hardware implementation and to [43] for an applicability study of these circuits.

In contrast to the long-term learning, the implemented *short-term plasticity* mechanism (STP) decays over several hundreds of milliseconds. It is motivated by the phenomenological model by [44] and depends only on the pre-synaptic activity, therefore being implemented in the synapse driver. For every incoming spike, a synapse only has access to a portion 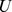 of the recovered partition 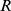 of its total synaptic weight 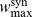, which then instantly decreases by a factor 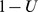 and recovers slowly along an exponential with the time constant 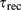, thus emulating synaptic depression. Facilitation is implemented by replacing the fixed 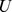 with a running variable 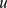, which increases with every incoming spike by an amount 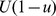 and then decays exponentially back to U with the time constant 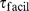:
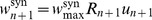
(6)

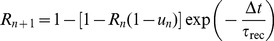
(7)

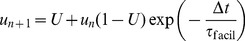
(8)with 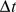 being the time interval between the *n*th and (*n*+1)st afferent spike. In contrast to the original Tsodyks-Markram (TSO) mechanism, the hardware implementation does not allow simultaneous depression and facilitation [34], [45]. See section S1.1 in Appendix S1 for details about the hardware implementation and the translation of the original model to the hardware STP.

All of the neuron and synapse parameters mentioned above are affected by fixed-pattern noise due to transistor-level mismatch in the manufacturing process. Additionally, the floating gate analog parameter storage reproduces the programmed voltage with a limited precision on each re-write. This leads to trial-to-trial variation for each experiment (see section S1.3 in Appendix S1 for exemplary measurements). Limited configurability, such as the discretization of available synaptic weights, is another source for discrepancy between targeted and realized configuration. The trial-to-trial variability, which cannot be remedied by calibration (section 2.2), is assumed to be less than 30% (standard-deviation-to-mean ratio) for synaptic weights. Other neuron parameters are assumed to have a much smaller variability. 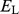, 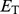, 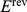 have a standard deviation of less than 1 mV in the biological domain (cf. section 2.2 and section S1.3 in Appendix S1). In this publication, we limit all investigations to the variation of synaptic weights, as they are assumed to be the dominant effect. To accomodate the total effect of trial-to-trial and fixed-pattern variation as well as parameter discretization, we simulate deviations of up to 50% (cf. section 2.4).

For technical details about the HICANN chip and its components we refer to [27], [34].

#### 2.1.2 Communication infrastructure

The infrastructure for pulse communication in the wafer-scale system is supplied by a two-layer approach: While the on-wafer network routes pulses between neurons on the same wafer, the off-wafer network connects the wafer to the outside world, i.e. to the host PC or to other wafers.

The backbone of the *on-wafer communication* consists of a grid of horizontal and vertical buses enabling the transport of action potentials by a mixture of time division and space division multiplexing. Each HICANN building block contains 64 horizontal buses at its center and 128 vertical buses located on each side of the synapse blocks, as can be seen in Figure 3. A bus can carry the spikes of up to 64 source neurons by transmitting a serial 6-bit signal encoding the currently sending neuron (with an ID from 0 to 63). When a neuron fires, its pulse is first processed by one of eight priority encoders and finally injected into a horizontal bus after passing a merger stage. By enabling a static switch of a sparse crossbar between horizontal and vertical buses, the injected serial signal can be made available to a vertical bus next to the synapse array. Another sparse switch matrix allows to feed the signals from the vertical buses into the synapse array, more precisely into the synapse drivers which represent the data sinks of the routing network. Synapse drivers can be connected in a chain, forwarding their input to their top or bottom neighbours, thereby allowing to increase the number of synapse rows fed by the same routing bus. The bus lanes do not end at the HICANN border but run over the whole wafer by edge-connecting the HICANN building blocks (Figure 2). We note that, due to electrotechnical reasons, the switches could not be implemented as full matrices, thus their sparseness was chosen as a compromise still providing maximum flexibility for implementing various neural network topologies [27], [32]. Both the sparseness of the switches and the limited number of horizontal and vertical buses represent a possible restriction for the connectivity of network models. If an emulated network requires a connectivity that exceeds the on-wafer bus capacity, some synapses will be impossible to map to the wafer and will therefore be lost.

Pulse propagation delays in the routing network are small, distance-dependent and not configurable: the time between spike detection and the onset of a post-synaptic potential (PSP) has been measured as 120 ns for a recurrent connection on a HICANN. The additional time needed to transmit a pulse across the entire wafer is typically less than 100 ns [34], hence the overall delay sums up to 1.2–2.2 ms in the biological time domain, assuming a speedup factor of 10^4^. Also, in case of synchronous bursting of the neurons feeding one bus, some pulses are delayed with respect to others, as they are processed successively: A priority encoder handles the spikes of 64 hardware neurons with priority fixed by design. If several neurons have fired, the pulse of the neuron with highest priority is transmitted first to the connected horizontal bus. The priority encoder can process one pulse every two clock cycles (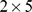 ns), leading to an additional delay for the pulses with lower priority. In rare cases some pulses may be completely discarded, e.g., when the total rate of all 64 neurons feeding one bus exceeds 10 kHz for longer than 6.4 ms (in biological real-time).

A hierarchical packet-based network provides the infrastructure for *off- and inter-wafer communication*. All HICANNs on the wafer are connected to the surrounding system and to other wafers via 12 pulse communication subgroups (PCS). Each PCS consists of one FPGA (Field Programmable Gate Array) and 4 ASICs (Application Specific Integrated Circuits) that were designed for high-bandwidth pulse-event communication (so-called Digital Network Chips or DNCs). Being the only communication link to/from the wafer, the off-wafer network also transports the configuration and control information for all the circuits on the wafer. As depicted in Figure 2, the network is hierarchically organized: one FPGA is connected to four DNCs, each of which is connected to 8 HICANNs of a reticle. Each FPGA is also connected to the host PC and potentially to up to 4 other FPGAs. When used for pulse-event communication, an FPGA-DNC-HICANN connection supports a throughput of 40 Mevents/s [46] with a timing precision of 4 ns. In the biological time domain, this corresponds to monitoring the spikes of all 512 neurons on a HICANN firing with a mean rate of 8 Hz each with a resolution of 0.04 ms. The same bandwidth is available simultaneously in the opposite direction, allowing a flexible network stimulation with user-defined spiketrains. For each FPGA-DNC-HICANN connection there are 512 pulse addresses that have to be subdivided into blocks of 64 used for either stimulation or recording. For all technical details about the PCS, the FPGA design and the DNC, we refer to [47]–[49].

Although the off-wafer communication interface allows the interconnection of multiple wafers, we restrict our studies here to the use of a single wafer.

### 2.2 Software Framework

The utilized software stack [40] allows the user to define a network description and maps it to a hardware configuration.

The network definition is accomplished by using PyNN [50], a simulator-independent API (Application Programming Interface) to describe spiking neural network models. It can interface to several simulation platforms such as NEURON [51] or NEST [52] as well as to neuromorphic hardware platforms [53], [54].

The mapping process [40], [55] translates the PyNN description of the neural network structure, as well as its neuron and synapse models and parameters, in several steps into a neuromorphic device configuration. This translation is constrained by the architecture of the device and its available resources.

The first step of the mapping process is to allocate static structural neural network elements to particular neuromorphic components during the so-called *placement*. Subsequently, a *routing* step is executed for establishing connections in between the placed components. During the final *parameter transformation* step, all parameters of the network components (neurons and synapses) are translated into hardware parameters. First, the model parameters are transformed to the voltage and time domain of the hardware, taking into account the acceleration and the voltage range of 0 V to 1.8 V [36]. Second, previously obtained *calibration* data is used to reduce mismatches between ideal neuromorphic circuitry behavior and real analogue signal hardware behavior.

The objective of the mapping process is to find a configuration of the hardware that best reproduces the neural network experiment specified in PyNN. The most relevant constraints are sketched in the following:

Each hardware neuron circuit has a limited number of 224 incoming synapses. By interconnecting several neuron circuits one can form “larger” neurons with more incoming synapses (Section 2.1.1), with the trade-off that the overall number of neurons is reduced. Still, each hardware synapse can not be used to implement a connection from an arbitrary neuron but only from a subset of neurons, namely the 64 source neurons whose pulses arrive at the corresponding synapse driver. For networks larger than 10 000 neurons it is the *limited number of inputs* to one HICANN that becomes even more restricting, as there are only 224 synapse drivers (cf. Figure 3), yielding a maximum of 14366 different source neurons for all neurons that are placed to the same HICANN. Hence, one objective of the mapping process is to reduce this number of source neurons per HICANN, thus increasing the number of realized synapses on the hardware. In general, this criterion is met when neurons with common pre-synaptic partners are placed onto the same HICANN and neurons with common targets inject their pulses into the same on-wafer routing bus.

All of the above, as well as the limited number of on-wafer routing resources (section 2.1.2) make the mapping optimization an NP-hard problem. The used placement and routing algorithms, which improve upon the ones described in [40] and [32] but are far from being optimal, can minimize the effect of these constraints only to a certain degree. Thus, depending on the network model size, its connectivity, and the choice of the mapping algorithms, *synapses are lost* during the mapping process; in other words, some synapses of a network defined in PyNN will be inexistent in the corresponding network emulated on the hardware. For an estimation of the amount of synapse loss, we first scaled all three benchmark models to sizes between 1000 and 100 000 neurons and mapped them onto the hardware using a simple, not optimized placement strategy. The results strongly depend on the size and the connectivity structure of the emulated network. In order to allow a comprehensive discussion within this study, we then used various placement strategies, sometimes optimizing the mapping by hand to minimize the synapse loss, or purposely using a wasteful allocation of resources to generate synapse loss.

### 2.3 Executable System Specification (ESS)

The ESS is a detailed simulation of the hardware platform [40], [56] that replicates the topology and dynamics of the communication infrastructure as well as the analog synaptic and neuronal components.

The simulation encompasses a numerical solution of the equations that govern the hardware neuron and synapse dynamics (Equations 1 to 5 and Equations S1.1 to S1.3 in Appendix S1) and a detailed reproduction of the digital communication infrastructure at the level of individual spike transmission in logical hardware modules. The ESS is a *specification* of the hardware in the sense that its configuration space faithfully maps the possible interconnection topologies, parameter limits, parameter discretization and shared parameters. Being executable, the ESS also covers dynamic constraints, such as the consecutive processing of spikes which can lead to spike time jitter or spike loss. Variations in the analog circuits due to production variations are not simulated at transistor level but are rather artificially imposed on ideal hardware parameters. In this article, only synaptic weight noise is considered, as detailed in section 2.4. All of this allows to simultaneously capture the complex dynamic behavior of the hardware and comply with local bandwidth limitations, while allowing relatively quick simulations due to the high level of abstraction. Simulations on the ESS can be controlled using PyNN (Section 2.2), similarly to any other PyNN-compatible back-end. Both for the real hardware and for the ESS, the mapping process translates a PyNN network into a device configuration, which is then used as an input for the respective back-end. One particular advantage of the ESS is that it allows access to state variables which can otherwise not be read out from the real hardware, such as the logging of lost or jittered time events.

### 2.4 Investigated Distortion Mechanisms

Reviewing the hardware and software components of the BrainScaleS wafer-scale system (Section 2.1 and 2.2) leaves us with a number of mechanisms that can affect or impede the emulation of neural network models:

- neuron and synapse models are cast into silicon and can not be altered after chip production
- limited ranges for neuron and synapse parameters
- discretized and shared parameters
- limited number of neurons and synapses
- restricted connectivity
- synapse loss due to non-optimal algorithms for NP-hard mapping
- parameter variations due to transistor level mismatch and limited re-write precision
- non-configurable pulse delays and jitter
- limited bandwidth for stimulation and recording of spikes

It is clear that, for all of the above distortion mechanisms, it is possible to find a corner case where network dynamics are influenced strongly. However, a few of these effects stand out: on one hand, they are of such fundamental nature to mixed-signal VLSI that they are likely to play some role in any similar neuromorphic device; on the other hand, they are expected to influence any kind of emulated network to some extent. We have therefore directed our focus towards these particular effects, which we summarize in the following. In order to allow general assessments, we investigate various magnitudes of these effects, also beyond the values we expect for our particular hardware implementation.

#### Neuron and synapse models

While some network architectures employ relatively simple neuron and synapse models for analytical and/or numerical tractability, others rely on more complex components in order to remain more faithful to their biological archetypes. Such models may not allow a straightforward translation to those available on the hardware, requiring a certain amount of fitting. In our particular case, we search for parameters to Equations 1 to 5 and Equations S1.1 to S1.3 in Appendix S1 that best reproduce reproduce low-level dynamics (e.g. membrane potential traces for simple stimulus patterns) and then tweak these as to optimally reproduce high-level network behaviors. Additionally, further constraints are imposed by the parameter ranges permitted by the hardware (Table S1.1 in Appendix S1).

#### Synapse loss

Above a certain network size or density, the mapping process may not be able to find enough hardware resources to realize every single synapse. We use the term “synapse loss” to describe this process, which causes a certain portion of synaptic connections to be lost after mapping. In a first stage, we model synapse loss as homogeneous, i.e., each synapse is deleted with a fixed probability between 0 and 50%. To ease the analysis of distortions, we make an exception for synapses that mediate external input, since, in principle, they can be prioritized in the mapping process such that the probability of losing them practically vanishes. Ultimately however, the compensation methods designed for homogeneous synapse loss are validated against a concrete mapping scenario.

#### Non-configurable axonal delays

Axonal delays on the wafer are not configurable and depend predominantly on the processing speed of digital spikes within one HICANN, but also on the physical distance of the neurons on the wafer. In all simulations, we assume a constant delay of 1.5 ms for all synaptic connections in the network, which represents an average of the expected delays when running the hardware with a speedup of 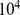 with respect to real time.

#### Synaptic weight noise

As described in section 2.1.1, the variation of synaptic weights is assumed to be the most significant source of parameter variation within the network. This is due to the coarser discretization (4-bit weight vs. 10 bit used for writing the analog neuron parameters) as well as the large number of available synapses, which prohibits the storage of calibration data for each individual synapse. The quality of the calibration only depends on the available time and number of parameter settings, while the trial-to-trial variability and the limited setting resolution remains. To restrict the parameter space of the following investigations, only the synaptic weights are assumed to be affected by noise. In both software and ESS simulations, we model this effect by drawing synaptic weights from a Gaussian centered on the target synaptic weight with a standard-deviation-to-mean-ratio between 0 and 50%. Occasionally, this leads to excitatory synapses becoming inhibitory and vice versa, which can not happen on the hardware. Such weights are clipped to zero. Note that this effectively leads to an increase of the mean of the distribution, which however can be neglected, e.g., for 50% noise the mean is increased by 0.425%. For ESS simulations we assume a synaptic weight noise of 20%, as test measurements on the hardware indicate that the noise level can not be reduced to below this number.

It has to be noted that the mechanism of distortion plays a role in the applicability of the compensation mechanisms. The iterative compensation in section 3.3.6 is only applicable when the dominant distortion mechanism is fixed-pattern noise. The other compensation methods, which do not rely on any kind of knowledge of the fixed-pattern distribution, function independently of the distortion mode.

### Hardware-Induced Distortions and Compensation Strategies

**Figure 4:**
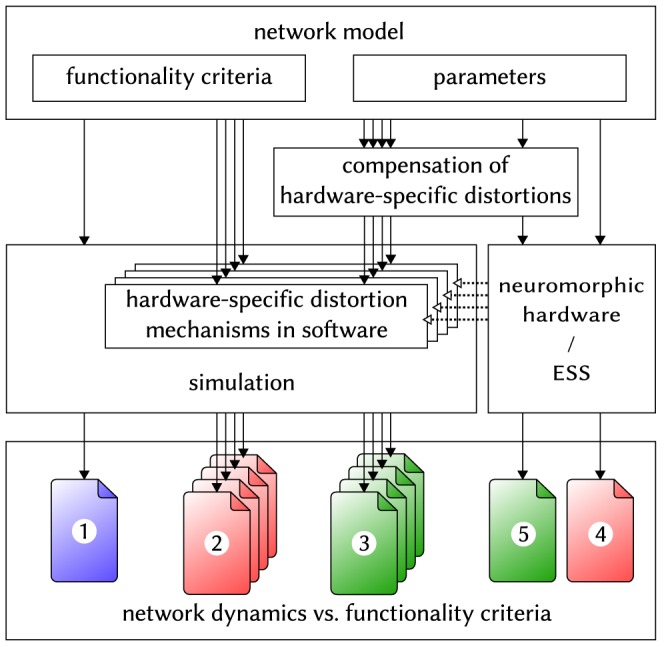
Schematic of the workflow used for studying and compensating hardware-induced distortions of network dynamics. (1) A given network model is defined by providing suitable parameters (for its connectivity and components) and well-defined functionality criteria. (2) The distortion mechanisms that are expected to occur natively on the hardware back-end are implemented and studied individually in software simulations. (3) Compensation methods are designed and tested, with the aim of recovering the original network dynamics as determined by the functionality criteria. (4) The network model is run on the hardware (here: the ESS) without any compensation to evaluate the full effect of the combined distortion mechanisms. (5) The compensation methods are combined and applied to the hardware (here: the ESS) simulation in order to restore the original network dynamics.

In the following, we analyze the effects of hardware-specific distortion mechanisms on a set of neuronal network models and propose adequate compensation mechanisms for restoring the original network dynamics. The aim of these studies is twofold. On one hand, we propose a generic workflow which can be applied for different neural network models regardless of the neuromorphic substrate, assuming it posesses a certain degree of configurability (Figure 4). On the other hand, we seek to characterize the universality of the BrainScaleS neuromorphic device by assessing its capability of emulating very different large-scale network models with minimal, if any, impairment to their functionality.

In order to allow a comprehensive overview, the set of benchmark experiments is required to cover a broad range of possible network architectures, parameters and function modi. To this end, we have chosen three very different network models, each of which highlights crucial aspects of the biology-to-hardware mapping procedure and poses unique challenges for the hardware implementation. In order to facilitate the comparison between simulations of the original model and their hardware implementation, all experimental setups were implemented in PyNN, running the same set of instructions on either simulation back-end.

For each of our benchmark models we define a set of specific well-quantifiable functionality criteria. These criteria are measured in software simulations of the ideal, i.e., undistorted network, which is then further referenced as the “original”.

Assuming that the broad range of hardware-specific distortion mechanisms affects various network parameters, their impact on these measures are investigated in software simulations and various changes to the model structure are proposed in order to recover the original functionality. The feasibility of these compensation methods is then studied for the BrainScaleS neuromorphic platform with the help of the ESS described in Section 2.3.

All software simulations were performed with NEST [57] or Neuron [58].

### 3.1 Cortical Layer 2/3 Attractor Memory

As our first benchmark, we have chosen an attractor network model of the cerebral cortex which exihbits characterisic and well-quantifiable dynamics, both at the single-cell level (membrane voltage UP and DOWN states) and for entire populations (gamma band oscillations, pattern completion, attentional blink). For this model, the mapping to the hardware was particularly challenging, due to the complex neuron and synapse models required by the original architecture on the one hand, as well as its dense connectivity on the other. In particular, we observed that the shape of synaptic conductances strongly affects the duration of the attractor states. As expected for a model with relatively large populations as functional units, it exhibits a pronounced robustness to synaptic weight noise. Homogeneous synapse loss, on the other hand, has a direct impact on single-cell dynamics, resulting in significant deviations from the expected high-level functionality, such as the attenuation of attentional blink. As a compensation for synapse loss, we suggest two methods: increasing the weights of the remaining synapses in order to maintain the total average synaptic conductance and reducing the size of certain populations and thereby decreasing the total number of required synapses. After mapping to the hardware substrate, synapse loss is not homogeneous, due to the different connectivity patterns of the three neuron types required by the model. However, we were able to apply a population-wise version of the suggested compensation methods and demonstrate their effectiveness in recovering the previously defined target functionality measures.

#### 3.1.1 Architecture

As described in [59] and [60], this model (henceforth called L2/3 model) implements a columnar architecture [61], [62]. The connectivity is compliant with data from cat cerebral cortex connectivity [63]. The key aspect of the model is its modularity, which manifests itself on two levels. On a large scale, the simulated cortical patch is represented by a number 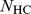 of hypercolumns (HCs) arranged on a hexagonal grid. On a smaller scale, each HC is further subdivided into a number 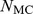 of minicolumns (MCs) [61], [62]. Such MCs should first and foremost be seen as functional units, and could, in biology, also be a group of distributed, but highly interconnected cells [64]–[66]. In the model, each MC consists, in turn, of 30 pyramidal (PYR), 2 regular spiking non-pyramidal (RSNP) and 1 basket (BAS) cells [67], [68]. Within each MC, PYR neurons are mutually interconnected, with 25% connectivity, such that they will tend to be co-active and code for similar input.

**Figure 5:**
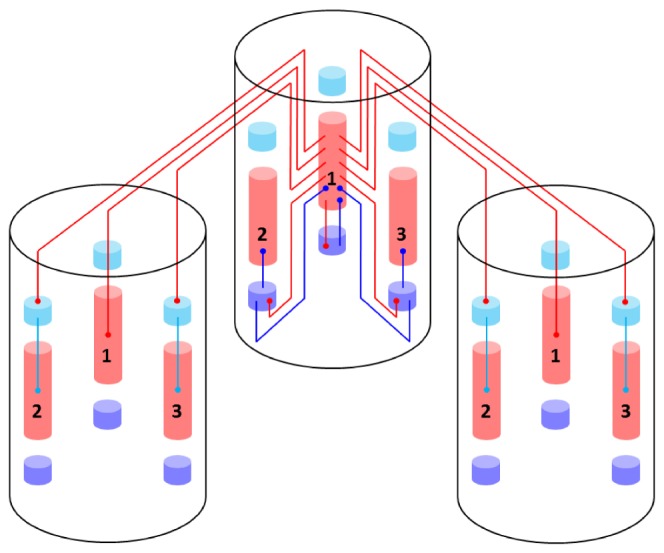
Pseudo-3D schematic of the layer 2/3 model architecture. Excitatory (PYR) cell populations are represented as red cylinders, inhibitory populations as blue ones (BAS: dark, RSNP: light). A minicolumn (MC) consists of three vertically aligned populations: one PYR, one BAS and one RSNP. Multiple MCs are grouped into hypercolumns (HCs, transparent cylinders). MCs with the same ID (one per HC) form a so-called pattern or attractor. When active, all PYR cells belonging to an attractor excite each other via short-range (within an MC) and long-range (between MCs) connections. The inhibition of PYR cells belonging to other attractors occurs via inhibitory interneurons: locally (within an HC) through BAS cells and globally (between HCs) through RSNP cells. Only a subset of connections are shown, namely those which are mainly used during active periods of attractor 1.

The functional units of the network, the MCs, are connected in global, distributed patterns containing a set of MCs in the network (Figure 5). Here the attractors, or patterns, contain exactly one MC from each HC. We have only considered the case of orthogonal patterns, which implies that no two attractors share any number of MCs. Due to the mutual excitation within an attractor, the network is able to perform pattern completion, which means that whenever a subset of MCs in an attractor is activated, the activity tends to spread throughout the entire attractor.

Pattern rivalry results from competition between attractors mediated by short and long-range connections via inhibitory interneurons. Each HC can be viewed as a soft winner-take-all (WTA) module which normalizes activity among its constituent MCs [60]. This is achieved by the inhibitory BAS cells, which receive input from the PYR cells from the 8 closest MCs and project back onto the PYR cells in all the MCs within the home HC. Apart from providing long-range connections to PYR cells within the same pattern, the PYR cells within an MC project onto RSNP cells in all the MCs which do not belong to the same pattern and do not lie within the same HC. The inhibitory RSNP cells, in turn, project onto the PYR cells in their respective MC. The effect of this connectivity is a disynaptic inhibition between competing patterns. Figure 5 shows a schematic of the default architecture, emphasizing the connectivity pattern described above. It consists of 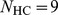 HCs, each containing 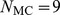 MCs, yielding a total of 2673 neurons. Due to its modular structure, this default model can easily be scaled up or down in size with preserved dynamics, as described in Appendix S2 (Section S2.4).

When a pattern receives enough excitation, its PYR cells enter a state reminiscent of a so-called local UP-state [69], which is characterized by a high average membrane potential, several mV above its rest value, and elevated firing rates. Pattern rivalry leads to states where only one attractor may be active (with all its PYR cells in an UP-state) at any given time. Inter-PYR synapses feature an STP mechanism which weakens the mutual activation of PYR cells over time and prevents a single attractor from becoming persistently active. Additionally, PYR neurons exhibit spike-frequency adaptation, which also suppresses prolonged firing. These mechanisms impose a finite life-time on the attractors such that after their termination more weakly stimulated or less excitable attractors can become active, in contrast to what happens in classical WTA networks.

The inputs to the layer 2/3 PYR cells arrive from the cortical layer 4, which is represented by 5 cells per MC. The layer 4 cells project onto the layer 2/3 PYR cells and can be selectively activated by external Poisson spike trains. Additionally, the network receives unspecific input representing activity in various connected cortical areas outside the modeled patch. This input is modeled as diffuse noise and generates a background activity of several Hz.

More details on the model architecture, as well as neuron and synapse parameters, can be found in Appendix S2.

#### 3.1.2 Functionality criteria

**Figure 6:**
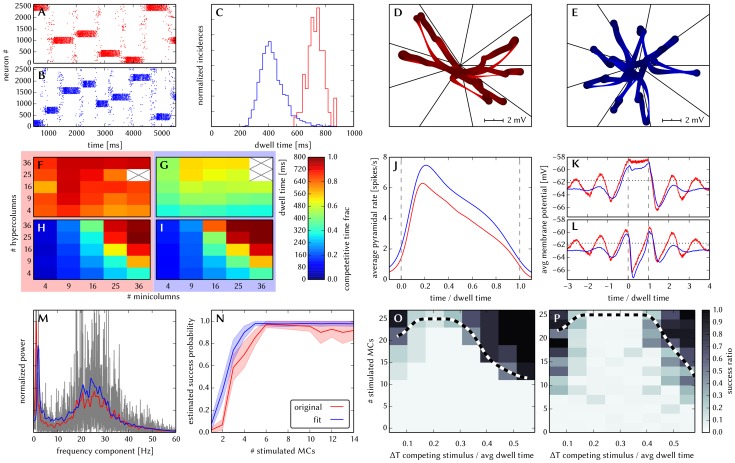
Comparison between original and adapted L2/3 network models. Unless explicitly stated otherwise, the default network model (9HC
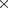
9MC) was used. Measurements from the original model are depicted (or highlighted) in red, while those from the adapted model are depicted (or highlighted) in blue. (A, B): Raster plots of spiking activity. Attractors activate spontaneously only due to diffuse background noise. Only PYR cells are shown. The MCs are ordered such that those belonging to the same attractor (and *not* those within the same HC) are grouped together. C: Attractor dwell time distributions. The shorter average dwell times in the adapted model are caused by sharper PSPs which miss the long NMDA time constants. (D, E): Star plots of average PYR cell voltages from a sample of 5 PYR cells per MC. Details on this representation of multidimensional data can be found in section S2.8 of Appendix S2. (F, G): Average dwell time for various network sizes. (H, I): Fraction of time spent in competitive states (i.e. no active attractors) for various network sizes. While dwell times remain relatively constant, competition times increase with network size, suppressing spontaneous attractors in large networks. (J): Average firing rate of PYR within an active period of their parent attractor. (K): Average voltage of PYR cells before, during and after their parent attractor is active (UP state). (L): Average voltage of PYR cells before, during and after an attractor they do not belong to is active (DOWN state). For subplots J, K and L, the abscissa has been subdivided into multiples of the average attractor dwell time in the respective simulations. The oscillations of the average voltages occur due to spike-triggered adaptation: after an active period, PYR cells need to recover before being able to support an active period of their home attractor, during which time they are inhibited by other active attractors. The more pronounced attenuation of the oscillations in the adapted model happens due to a higher relative variability of dwell times (compare subplot C). In subplots K and L the dotted line indicates the leak potential 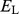 of the PYR cells. (M): Smoothed power spectrum of PYR firing rate averaged over all MCs. The grey curve in the background represents the unsmoothed spectral density for the original model. Attractor switches (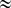 2 Hz) and gamma oscillations (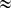
25 Hz) can be clearly observed. (N): Pattern completion in a 25HC
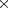
25MC network. Estimated probability of an attractor to fully activate (success ratio) as a function of the number of stimulated constituent MCs, measured over 25 trials per abscissa value. (O, P): Attentional blink in a 25HC
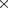
25MC network. Two attractors are stimulated (the second one only partially, i.e. a certain number of constituent MCs) with a temporal lag of 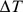 in between. Activation probability of the second attractor and 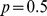 iso-probability contours, measured over 14 trials per (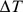, #MCs) pair. A detailed description of the data and methods used for all figures concerning the L2/3 model can be found in Appendix S2.

Figure 6 shows some characteristic dynamics of the L2/3 model, which have also been chosen as functionality criteria and are described below.

The core functionality of the original model is easily identifiable by its distinctive display of spontaneously activating attractors in e.g. raster plots (**A**) or voltage star plots (**D**, for an explanation of star plots see section S2.8 in Appendix S2). However, in particular for large network sizes, spontaneous attractors become increasingly sparse. Additionally, many further indicators of functionality can be found, such as the average membrane potential or the gamma oscillations observed in UP states. Finally, when receiving L4 stimulation in addition to the background noise, the original model displays important features such as pattern completion and attentional blink, which need to be reproducible on the hardware as well. Consequently, we consider several measures of functionality throughout our analyses.

When an attractor becomes active, it remains that way for a characteristic dwell time 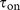. The dwell time depends strongly on the neuron and synapse parameters (as will be discussed in the following sections) and only weakly on the network size (**C**, **F**), since the scaling rules ensure a constant average fan-in for each neuron type. Conversely, this makes 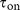 sensitive to hardware-induced variations in the average synaptic input. The detection of active attractors is performed automatically using the spike data (for a description of the algorithm, see section S2.5 in Appendix S2).

We describe the periods between active attractors as competition phases and the time spent therein as the total competition time. The competition time varies strongly depending on the network size (**H**). One can observe that the competition time is a monotonically increasing function of both 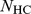 and 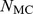. For an increasing number of HCs, i.e., a larger number of neurons in every pattern, the probability of a spontaneous activation of a sufficiently large number of PYR cells decreases. For an increasing number of MCs per HC, there is a larger number of competing patterns, leading to a reduced probability of any single pattern becoming dominant.

When an attractor becomes active, the average spike rate of its constituent PYR cells rises sharply and then decays slowly until the attractor becomes inactive again (**J**). Two independent mechanisms are the cause of this decay: neuron adaptation and synaptic depression. The characteristic time course of the spike rate depends only weakly on the size of the network.

As described in Section 3.1.1, PYR cells within active attractors enter a so-called local UP state, with an increased average membrane potential and an elevated firing rate (**K**). While inactive or inhibited by other active attractors, PYR cells are in a DOWN state, with low average membrane potential and almost no spiking at all (**L**). In addition to these characteristic states, the average PYR membrane potential exhibits oscillations with a period close to 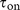. These occur because the activation probability of individual attractors is an oscillatory function of time as well. In the immediate temporal vicinity of an active period (i.e., assuming an activation at 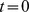, during 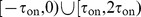) the same attractor must have been inactive, since PYR populations belonging to an activated attractor need time to recuperate from synaptic depression and spike-triggered adaptation before being able to activate again.

An essential emerging feature of this model are oscillations of the instantaneous PYR spike rate in the gamma band within active attractors (**M**). The frequency of these oscillations are independent of size and rather depend on excitation levels in the network [60]. Although the gamma oscillations might suggest periodic spiking, it is important to note that individual PYR cells spike irregularly (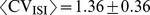 within active attractors).

Apart from these statistical measures, two behavioral properties are essential for defining the functionality of the network: the pattern completion and attentional blink mentioned above. The pattern completion ability of the network can be described as the successful activation probability of individual patterns as a function of the number of stimulated MCs (**N**). Similarly, the attentional blink phenomenon can also be quantified by the successful activation rate of an attractor as a function of the number of stimulated MCs if it is preceded by the activation of some other attractor with a time lag of 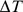 (**O**). For small 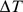, the second attractor is completely “blinked out”, i.e., it can not be activated regardless of the number of stimulated MCs. To facilitate the comparison between different realizations of the network with respect to attentional blink, we consider the 50% iso-line, which represents the locus of the input variable pair which leads to an attractor activation ratio of 50%. These functional properties are easiest to observe in large networks, where spontaneous attractors are rare and do not interfere with stimulated ones.

A detailed description of the data and methods used for these figures can be found in the Appendix S2.

#### 3.1.3 Neuron and synapse model translation

**Figure 7:**
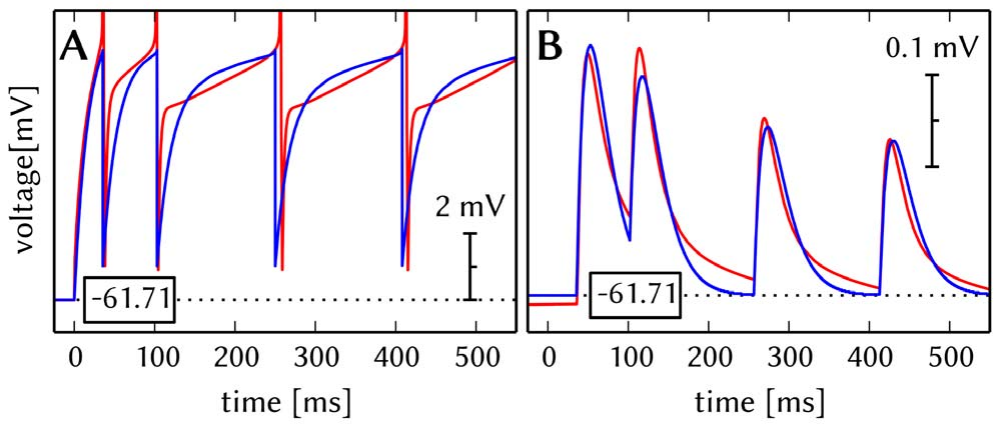
Comparison of original and fitted neuron and synapse dynamics. Original neuron (multi-compartment HH) and synapse (NMDA+AMPA) dynamics are shown in red, the fitted dynamics of hardware-compatible neuron (point AdEx) and synapse (single decay time constant) models in blue. (A) Membrane potential of PYR cells under spike-inducing current stimulation. While the precise membrane potential time course of the original neuron model can not be reproduced by a single-compartment AdEx neuron, spike timing and especially firing rates can be recovered. (B) PSPs generated by PYR
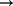
PYR synapses between MCs where the spikes from A were used as input. As a replacement for the multiple synaptic time constants in the original model, we have chosen an intermediate value for 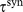, which constitutes the main reason for the difference in PSP shapes. Additionally, the combination of STP and saturation in the original model had to be replaced by STP alone.

A particular feature of this benchmark model is the complexity of both neuron and synapse models used in its original version. Therefore, the first required type of compensation concerns the parameter fitting for the models implemented on the hardware. Some exemplary results of this parameter fit can be seen in Figure 7. More details can be found in section S2.2 of Appendix S2.

*Neurons.* In general, the typical membrane potential time course during a spike of a Hodgkin-Huxley neuron can be well approximated by the exponential term in the AdEx equation [35]. However, when fitting for spike timing, we found that spike times were best reproduced when eliminating the exponential term, i.e. setting 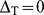.

Adaptation is an essential feature of both the PYR and the RSNP cells in the original model, where it is generated by voltage-dependent 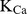 channels. We were able to reproduce the correct equilibrium spike frequency by setting the AdEx adaptation parameters *a* and *b* to nonzero values. One further difference resides in the original neurons being modeled as having several compartments, whereas the hardware only implements point neurons. The passive neuron properties (membrane capacitances and leak conductances) were therefore determined by fitting the membrane potential time course under stimulation by a step current which was not strong enough to elicit spikes.

*Synapses.* We have performed an initial estimation of synaptic weights and time constants by fitting the membrane potential time course of the corresponding neurons in a subthreshold regime. However, two important differences remain between the synapses in the original model and the ones available on our hardware.

In the original model, PYR-PYR and PYR-RSNP synapses contain two types of neurotransmitters: Kainate/AMPA and NMDA (see Table S2.2 in Appendix S2). Due to the vastly different time constants for neurotransmitter removal at the postsynaptic site (6 ms and 150 ms, respectively), the PSPs have a characteristic shape, with a pronounced peak and a long tail (red curve in Figure 7 **B**). While, in priniciple, the HICANN supports several excitatory time constants per neuron (Section 2.1.1), the PyNN API as well as the mapping process support only one excitatory time constant per neuron. With this limitation the PSP shape can not be precisely reproduced.

One further difference lies in the saturating nature of the postsynaptic receptor pools after a single presynaptic spike. In principle, this behavior could be emulated by the TSO plasticity mechanism by setting 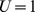 and 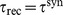. However, this would conflict with the TSO parameters required for modeling short-term depression of PYR synapses and would also require parameters outside the available hardware ranges.

For these reasons, we have further modified synaptic weights and time constants by performing a behavioral fit, i.e., by optimizing these parameters towards reproducing the correct firing rates of the three neuron types in two scenarios - first without and then subsequently with inhibitory synapses. Because the original model was characterized by relatively long and stable attractors, we further optimized the excitatory synapse time constants towards this behavior.

*Post-fit model behavior.* Figure 6 shows the results of the translation of the original model to hardware-compatible dynamics and parameter ranges. Overall, one can observe a very good qualitative agreeement of characteristic dynamics with the original model. In the following, we discuss this in more detail and explain the sources of quantitative deviations.

When subject to diffuse background noise only, the default size network clearly exhibits its characteristic spontanous attractors (**B**). Star plots exhibit the same overall traits, with well-defined attractors, characterized by state space trajectories situated close to the axes and low trajectory velocities within attractors (**E**). Attractor dwell times remain relatively stable for different network sizes, while the competition times increase along with the network size (**G** and **I**). The average value of dwell times, however, lies significantly lower than in the original (**C**). The reason for this lies mainly in the shape of EPSPs: the long EPSP tails enabled by the large NMDA time constants in the original model caused a higher average membrane potential, thereby prolonging the activity of PYR cells.

Within attractors, active and inactive PYR cells enter well-defined local UP and DOWN states, respectively (**K** and **L**). Before and after active attractors, the dampened oscillations described in section 3.1.2 can be observed. In the adapted model, attenuation is stronger due to a higher coefficient of variation of the dwell times (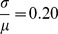 as compared to 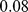 in the original model).

Average PYR firing rates within active attractors have very similar time courses (**J**), with a small difference in amplitude, which can be attributed to the difference in EPSP shapes discussed earlier. Both low-frequency switches between attractors (¡ 3 Hz, equivalent to the incidence rate) and high-frequency gamma oscillations arising from synchronous PYR firing (with a peak around 25 Hz) can be clearly seen in a power spectrum of the PYR firing rate (**M**).

Pattern completion occurs similarly early, with a steep rise and nearly 100% success rate starting at 25% of stimulated MCs per attractor (**N**). Attentional blink follows the same qualitative pattern (**P**, **Q**), although with a slightly more pronounced dominance of the first activated attractor in the case of the adapted network, which happens due to the slightly higher firing rates discussed above.

Having established the quality of the model fit and in order to facilitate a meaningful comparison, all following studies concerning hardware-induced distortions and compensation thereof use data from the adapted model as reference.

#### 3.1.4 Synapse loss

**Figure 8:**
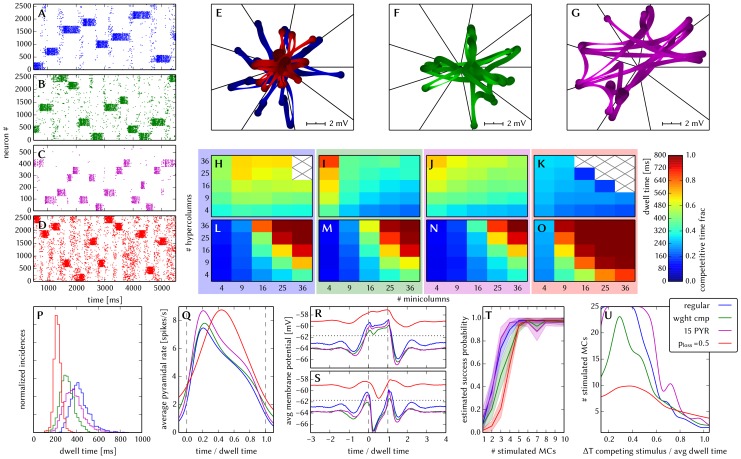
Compensation of homogeneous synaptic loss in the L2/3 model. Unless explicitly stated otherwise, the default network model (9HC
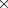
9MC) was used. Here, we use the following color code: blue for the original model, red for the distorted case (50% synapse loss), green for the compensation via increased synaptic weights and purple for the compensation by scaling down the size of the PYR populations. (A–D) Raster plots of spiking activity. The MCs are ordered such that those belonging to the same attractor (and *not* those within the same HC) are grouped together. Synapse loss weakens the interactions within and among MCs, causing shorter dwell times and longer competition times. Both compensation methods successfuly counter these effects. These phenomena can also be observed in subplots H–P. (E–G) Star plots of average PYR voltages from a sample of 5 PYR cells per MC. Synapse loss leads to a less pronounced difference between the average PYR membrane potential within and outside of active attractors. After compensation, the differences between UP and DOWN states become more pronounced again. These phenomena can also be observed in subplots R and S. (H–K) Average dwell time for various network sizes. (L–O) Fraction of time spent in competitive states (i.e. no active attractors) for various network sizes. (P) Distributions of dwell times. (Q) Average firing rate of PYR cells within an active period of their parent attractor. (R) Average voltage of PYR cells before, during and after their parent attractor is active (UP state). (S) Average voltage of PYR cells before, during and after an attractor they do not belong to is active (DOWN state). For subplots Q, R and S, the abscissa has been subdivided into multiples of the average attractor dwell time in the respective simulations. In subplots R and S the dotted line indicates the leak potential 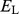 of the PYR cells. (T) Pattern completion in a 25HC
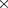
25MC network. Estimated activation probability from 25 trials per abscissa value. Synapse loss shifts the curve to the right, i.e., more MCs need to be stimulated to achieve the same probability of activating their parent attractor. Both compensation methods restore the original behavior to a large extent. (U) Attentional blink in a 25HC
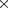
25MC network: 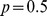 iso-probability contours, measured over 14 trials per (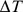, #MCs) pair. Synapse loss suppresses attentional blink, as inhibition from active attractors becomes to weak to prevent the activation of other stimulated attractors. Compensation by increasing the weight of the remaining synapses alleviates this effect, but scaling down the PYR population sizes directly reduces the percentage of lost synapses and is therefore more effective in restoring attentional blink.

The effects of homogeneous synapse loss and the results of the attempted compensation are depicted in Figure 8. More detailed plots can be found in Appendix S2 (Figure S2.3).

*Effects.* With increasing synapse loss, the functionality of the network gradually deteriorates. Attractors become shorter or disappear entirely, with longer periods of competition in between (**D**, **K**, **O**).

While average excitatory conductances are only affected linearly by synaptic loss, inhibitory conductances feel a compound effect of synapse loss, as it affects both afferent and efferent connections of inhibitory interneurons. Therefore, synapse loss has a stronger effect on inhibition, leading to a net increase in the average PYR membrane potential (**R**, **S**). Additionally, since all connections become weaker, the variance of the membrane potential becomes smaller, as observed in the corresponding star plots as well (**E**). The weaker connections also decrease the self-excitation of active attractors while decreasing the inhibition of inactive ones, thereby leading to shorter attractor dwell times (**P**). Somewhat surprisingly, the maximum average PYR firing rate in active attractors remains almost unchanged when subjected to synapse loss. However, the temporal evolution of the PYR firing rate changes significantly (**Q**).

The pattern completion ability of the network suffers particularly in the region of weak stimuli, due to weaker internal excitation of individual attractors. The probability of triggering a partially stimulated pattern can drop by more than 50% (**T**). Due to the decreased stability of individual attractors discussed above, rival attractors are easier to excite, thereby significantly suppressing the attentional blink phenomenon (**U**).

*Compensation.* As a first-order approximation, we can consider the population average of the neuron conductance as the determining factor in the model dynamics. For synapses with exponential conductance courses, the average conductance generated by the *i*th synapse is proportional to both synaptic weight 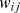 and afferent firing rate 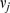. Because conductances sum up linearly, the total conductance that a neuron from population 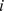 receives from some other population 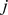 is, on average (see Equation S2.6 in Appendix S2)
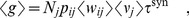
(9)where 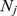 represents the size of the presynaptic population and 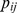 represents the probability of a neuron from the presynaptic population to project onto a neuron from the postsynaptic population. Since homogeneous synapse loss is equivalent to a decrease in 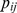, we can compensate for synapse loss that occurs with probability 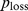 by increasing the weights of the remaining synapses by a factor 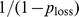. Figure 8 shows the results of this compensation strategy for 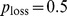. In all aspects, a clear improvement can be observed. The remaining deviations can be mainly attributed to two effects. First of all, preserving the average conductance by compensating homogeneous synapse loss with increasing synaptic weights leads to an increase in the variance of the membrane potential (Equation S2.5 in Appendix S2). Secondly, finite population sizes coupled with random elimination of synapses lead to locally inhomogeneous synapse loss and further increase the variability of neuronal activity.

Instead of compensating for synapse loss after its occurrence, it is also possible to circumvent it altogether after having estimated the expected synapse loss in a preliminary mapping run. For the L2/3 model, this can be done without altering the number of functional units (i.e., the number of HCs and MCs) by changing the size of the PYR cell populations. For this approach, however, the standard scaling rules (Section S2.4 in Appendix S2) need to be modified. These rules are designed to keep the average number of inputs per neuron constant and would increase the total number of PYR-incident synapses by the same factor by which the PYR population is scaled. This would inevitably lead to an increased number of shared inputs per PYR cell, with the immediate consequence of increased firing synchrony. Instead, when reducing the PYR population size, we compensate for the reduced number of presynaptic partners by increasing relevant synaptic weights instead of connection probabilities. This modified downscaling leads to a net reduction of the total number of synapses in the network, thereby potentially reducing synaptic loss between all populations. Figure 8 shows the effects of scaling down the PYR population size until the total remaining number of synapses is equal to the realized number of synapses in the distorted case (50% of the total number of synapses in the undistorted network). More detailed plots of the effects of PYR population downscaling can be found in Figure S2.4 of Appendix S2. The two presented compensation methods can also be combined to further improve the final result, as we show in Section 3.1.7.

#### 3.1.5 Synaptic weight noise

One would not expect the synaptic weight noise to affect the L2/3 model strongly, as it should average out over a large number of connections between the constitutent populations. It turns out that the surprisingly strong impact of synaptic weight noise is purely due to the implementation of background stimulus in this model and can therefore be easily countered.

*Effects.* The relative deviation of the total synaptic conductance scales with 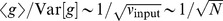 (see Equation S2.5 in Appendix S2), where 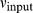 is the total input frequency and N the number of presynaptic neurons. Therefore, interactions between large populations are not expected to be strongly affected by synaptic weight noise.

The only connections where an effect is expected are the RSNP
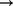
PYR connections, because the presynaptic RSNP population consists of only 2 neurons per MC. However, long-range inhibition also acts by means of a second-order mechanism, in which an active MC activates its counterpart in some other HC, which then in turn inhibits all other MCs in its home HC via BAS cells. This mechanism masks much of how synaptic weight noise affects RSNP
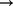
PYR connections.

**Figure 9:**
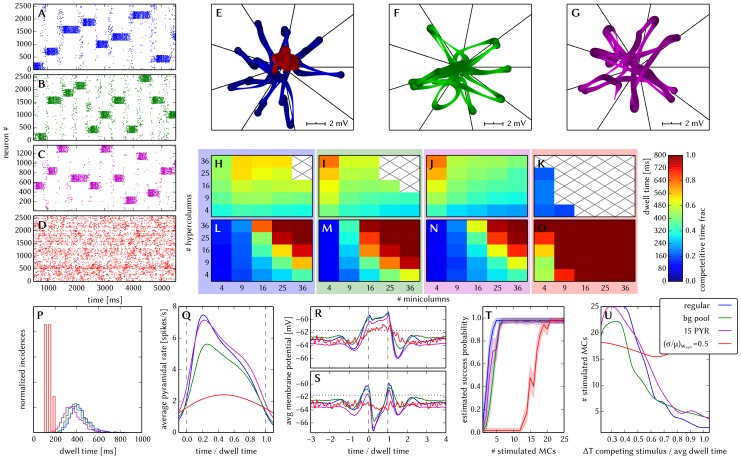
Compensation of synaptic weight noise in the L2/3 model. Unless explicitly stated otherwise, the default network model (9HC
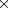
9MC) was used. Here, we use the following color code: blue for the original model, red for the distorted case (50% synaptic weight noise), green for the compensation via multiple background sources per PYR cell and purple for the same compensation method, but with scaled down PYR populations. Altogether, we note that the observed effects happen almost exclusively due to each PYR cell receiving background input via a single synapse. When compensated via the inclusion of multiple background sources, the network exhibits remarkable robustness towards synaptic weight noise. (A–D) Raster plots of spiking activity. The MCs are ordered such that those belonging to the same attractor (and *not* those within the same HC) are grouped together. When each PYR cell has a single background source, high levels of synaptic weight noise cause some PYR cells to become completely silent, while others spike disproportionately often. This can completely disrupt the stability of attractors, resulting in largely random spiking, with long competition times between the occasional appearance of weak, unstable attractors. The inclusion of multiple background sources per PYR cell efficiently counters these effects. This compensation strategy works just as well for downscaled PYR populations. The phenomena described above can also be observed in subplots H–P. (E–G) Star plots of average PYR voltages from a sample of 5 PYR cells per MC. The disrupted attractor behavior and erratic PYR spiking result in weak fluctuations of average PYR voltages with essentially no clear UP or DOWN states. After compensation, the differences between UP and DOWN states become more pronounced again. These phenomena can also be observed in subplots R and S. (H–K) Average dwell time for various network sizes. (L–O) Fraction of time spent in competitive states (i.e. no active attractors) for various network sizes. (P) Distributions of dwell times. (Q) Average firing rate of PYR cells within an active period of their parent attractor. (R) Average voltage of PYR cells before, during and after their parent attractor is active (UP state). (S) Average voltage of PYR cells before, during and after an attractor they do not belong to is active (DOWN state). For subplots Q, R and S, the abscissa has been subdivided into multiples of the average attractor dwell time in the respective simulations. In subplots R and S the dotted line indicates the leak potential 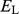 of the PYR cells. (T) Pattern completion in a 25HC
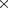
25MC network. Estimated activation probability from 25 trials per abscissa value. Due to erratically firing PYR cells in the distorted network, much stronger stimulation is needed to guarantee the appearance of an attractor. Compensation restores the original behavior to a large extent. (U) attentional blink in a 25HC
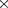
25MC network: 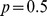 iso-probability contours, measured over 14 trials per (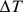, #MCs) pair. Due to the highly unstable attractors in the distorted network, attentional blink is completely suppressed. Compensation restores blink, but not to its original strength, due to the synaptic weight noise within the network itself.

Nevertheless, synaptic weight noise appears to have a strong effect on network dynamics (Figure 9, red curves). The reason for that lies in the way the network is stimulated. In the original model, each PYR cell receives input from a single Poisson source. This is of course a computational simplification and represents diffuse noise arriving from many neurons within other cortical areas. However, having only a single noise source connected by a single synapse to the target neuron makes the network highly sensitive to synaptic weight noise (see section S2.11 in Appendix S2).

*Compensation.* The compensation for this effect was done by increasing the number of independent noise sources per neuron, thereby reducing the statistically expected relative noise conductance variations per PYR cell. The only limitation lies in the total number of available external spike sources and the bandwidth supplied by the off-wafer communication network (Section 2.1.2). Once this limit is reached, the number of noise inputs per PYR cell can still be increased even further if PYR cells are allowed to share noise sources. Given a total number of available Poisson sources 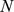 and a noise population size of 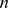 sources per PYR cell, the average pairwise overlap between two such populations is 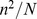. Therefore, as long as the average overlap remains small enough, the overlap-induced spike correlations will not affect the network dynamics.

In our example (Figure 9, green curves), we have chosen 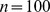, while the total number of Poisson sources is set at 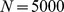. Note how this relatively simple compensation method efficiently restores most functionality criteria. The most significant remaining differences can be seen in pattern completion and attentional blink (**T**, **U**) and appear mainly due to the affected RSNP
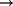
PYR connections.

In addition to the investigation of synaptic weight noise on the default model, we repeated the same experiments for the model with reduced PYR population sizes (Figure 9, purple curves), which we have previously suggested as a compensation method for synaptic weight noise (Section 3.1.4). The fact that PYR population reduction does not affect the network functionality in the case of (compensated) synaptic weight noise is an early indicator for the compatibility of the suggested compensation methods when all distortion mechanisms are present (Section 3.1.7).

#### 3.1.6 Non-configurable axonal delays

In the original model, axonal delays between neurons are proportional to the distance between their home HCs. At an axonal spike propagation velocity of 0.2 m/ms, the default (9HC
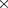
9MC) network implements axonal delays distributed between 0.5 and 8 ms. While PYR cells within an MC tend to spike synchronously in gamma waves, the distribution of axonal delays reduces synchronicity between spike volleys of different MCs.

**Figure 10:**
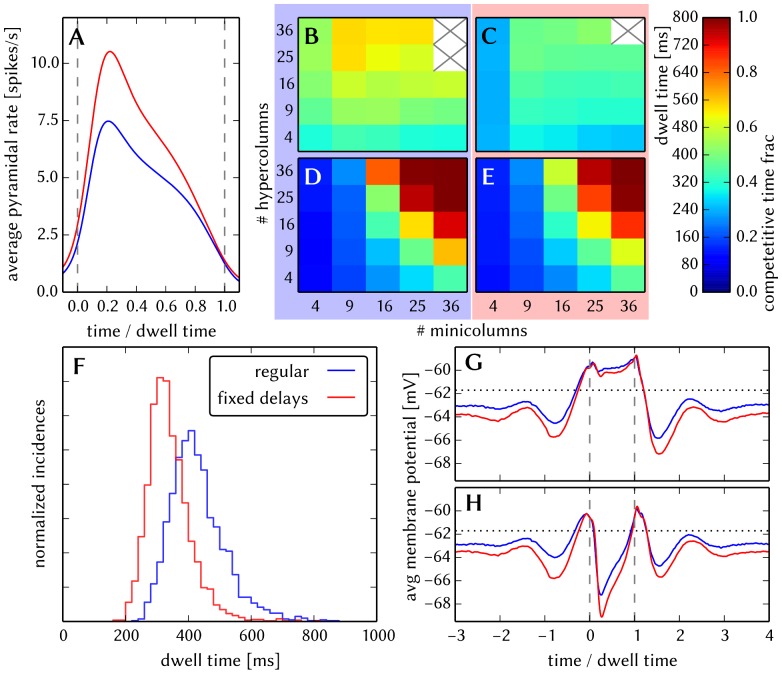
Effects of fixed axonal delays on the L2/3 model. Unless explicitly stated otherwise, the default network model (9HC
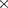
9MC) was used. Data from the regular and distorted models is depicted (or highlighted) in blue, and red, respectively. (A) Average firing rate of PYR cells within an active period of their parent attractor. (B, C) Average dwell time for various network sizes. (D, E) Fraction of time spent in competitive states (i.e. no active attractors) for various network sizes. (F) Distributions of dwell times. (G) Average voltage of PYR cells before, during and after their parent attractor is active (UP state). (H) Average voltage of PYR cells before, during and after an attractor they do not belong to is active (DOWN state). For subplots A, G and H, the abscissa has been subdivided into multiples of the average attractor dwell time in the respective simulations. In subplots G and H the dotted line indicates the leak potential 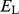 of the PYR cells.

Fixed delays, on the other hand, promote synchronicity, thereby inducing subtle changes to the network dynamics (Figure 10). The synchronous arrival of excitatory spike volleys causes PYR cells in active attractors to spike more often (**A**). Their higher firing rate in turn causes shorter attractor dwell times, due to their spike frequency adaptation mechanism (**B**, **C**, **F**). During an active attractor, the elevated firing rate of its constituent PYR cells causes a higher firing rate of the inhibitory interneurons belonging to all other attractors. This, in turn, leads to a lower membrane potential for PYR cells during inactive periods of their parent attractor (**G**, **H**). As these effects are not fundamentally disruptive and also difficult to counter without significantly changing other functional characteristics of the network, we chose not to design a compensation strategy for this distortion mechanism in the L2/3 network.

#### 3.1.7 Full simulation of combined distortion mechanisms

In a final step, we emulate the L2/3 model on the ESS (Section 2.3), and compensate simultaneously for all of the effects discussed above. We first investigate how much synapse loss to expect for different network sizes, and then realize the network at two different scales in order to investigate all of the chosen functionality criteria. The default network (9HC
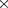
9MC) is used to analyze spontaneous attractors, while a large-scale model (25HC
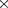
25MC) serves as the test substrate for pattern completion and pattern rivalry.

**Figure 11:**
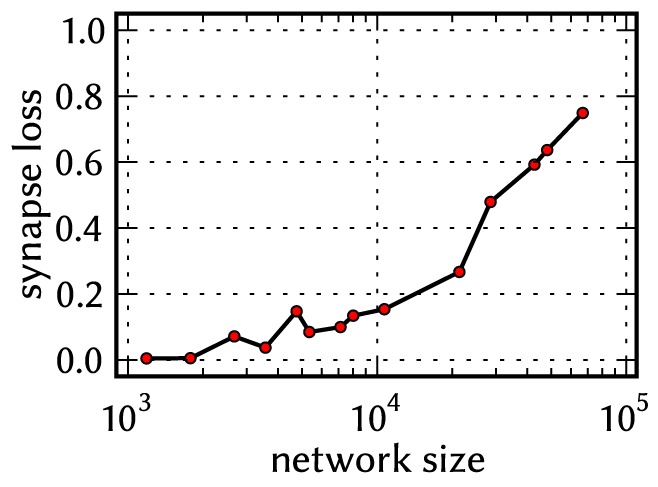
Synapse Losses after mapping the L2/3 model.

*Synapse loss.* The synapse loss after mapping the L2/3 model onto the BrainScaleS hardware is shown in Figure 11 for different sizes, using the scaling rules defined in section S2.4 of Appendix S2. Synapse loss starts to occur already at small sizes and increases rapidly above network sizes of 20 000 neurons. The jumps can be attributed to the different ratios between number of HCs and number of MCs per HC (Table S2.10 in Appendix S2).

**Figure 12:**
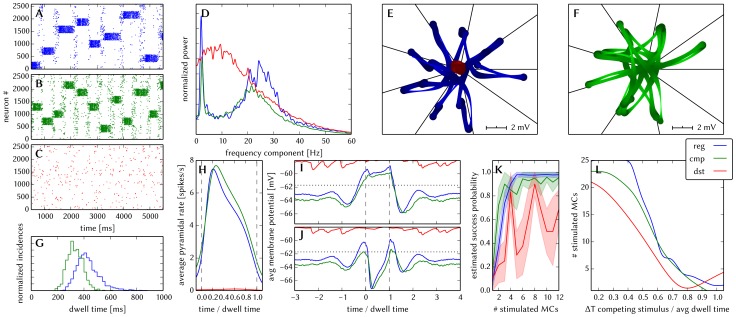
ESS emulation of the L2/3 model. Unless explicitly stated otherwise, the default network model (9HC
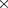
9MC) was used. Here, we use the following color code: blue for the original model, red for the distorted case on the ESS (with 20% synaptic weight noise and 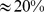 synapse loss), and green for the compensated case on the ESS. (A–C) Raster plots of spiking activity. The MCs are ordered such that those belonging to the same attractor (and *not* those within the same HC) are grouped together. A synapse loss of 32% on the background synpases (see Table 1) is the main reason for which no spontaneous attractors are evoked. For this reason, there are no red curves in G, H, I and J. Applying a weight compensation and increasing the number of background sources from 9 to 50 effectively restores the original behavior. (D) Power spectrum of global activity. Since no spontaneous attractors are evokes, neither attractor switching (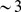
Hz) nor gamma oscillations (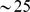
Hz) can be observed. The spectrum of the distorted network complies with the asynchronous irregular firing observed in C. Compensation restores both of the characteristic peaks in the spectrum. (E and F) Star plots of average PYR voltages from a sample of 5 PYR cells per MC. The disrupted attractor behavior results in a weak fluctuations of average PYR voltages with essentially no clear UP or DOWN states. After compensation, the differences between UP and DOWN states become more pronounced again. (G) Distributions of dwell times. The disrupted network effectively shows no spontaneous attractors. As expected from the software simulations, the dwell times remain, on average, slightly shorter after compensation. (H) Average firing rate of PYR cells within an active period of their parent attractor. The higher firing rates after compensation are caused by the fixed, short delays, which promote synchronous firing and therefore stronger mutual excitation among PYR cells. (I) Average voltage of PYR cells before, during and after their parent attractor is active (UP state). (J) Average voltage of PYR cells before, during and after an attractor they do not belong to is active (DOWN state). For subplots H, I and J, the abscissa has been subdivided into multiples of the average attractor dwell time in the respective simulations. In subplots I and J the dotted line indicates the leak potential 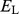 of the PYR cells. (K) Pattern completion in a 25HC
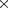
25MC network. Estimated activation probability from 25 trials per abscissa value. (L) Attentional blink in a 25HC
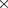
25MC network: 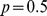 iso-probability contours, measured over 14 trials per (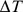, #MCs) pair. Since the distorted network showed no spontaneous attractors (C), we used the average dwell time from the pattern completion experiment (K) for normalization.

*Small-scale model.* The default model (9HC
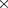
9MC) can, in principle, be mapped onto the hardware without any synapse loss if the full wafer is available for use. Nevertheless, in some scenarios, a full wafer might not be available, due to faulty components or part of its area being used for emulating other parts of a larger parent network. We simulate this scenario by limiting the usable wafer area to 4 reticles (out of a total of 48 on the full wafer). With the reduced available hardware size, the available pulse bandwidth of the off-wafer communication network decreases as well, such that diffusive background noise can not be modeled with one individual Poisson source per neuron. Hence, each pyramidal neuron receives input from 9 out of 2430 background sources. The total synapse loss for the given network setup amounts to 22.2% and affects different projection types with varying strength (Table 1). Also external synapses are lost, since, in contrast to the synapse loss study (Section 3.1.4), they have not been prioritized in the mapping process in this case. Additionally, we applied 20% synaptic weight noise and simulated the network with a speedup factor of 12 000. The behavior on the ESS is shown in Figure 12. The distorted network shows no spontaneous attractors (**C**), which can be mainly attributed to the loss of over 32% of the background synpases. To recover the original network behavior, we first increased the number of background neurons per cell from 9 to 50 to compensate for synaptic weight noise, and also scaled the weights by 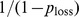 for each projection type with extracted synapse loss values 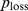 (Table 1), following the synapse loss compensation method described in Section 3.1.4. Note that here the complete PyNN experiment is re-run: synaptic weights are scaled in the network definition leading to a new configuration of 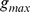 and the digital weights on the HICANNs (Section 2.1.1) after the mapping process. These measures effectively restored the attractor characteristics of the network (Figure 12). The attractor dwell times remained a bit smaller than for the regular network (**G**), which can be ascribed to the non-configurable delays (Section 3.1.6).

**Table 1:** Projection-wise synapse loss of the L2/3 model after the mapping process.

	9HC×9MC		25HC×25MC	
projection	dist.	comp.	dist.	comp.
PYR 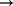 PYR (local)	21.1	21.0	0.9	0.3
PYR 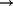 PYR (global)	20.8	21.2	8.0	0.4
PYR 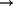 RSNP	22.6	21.9	37.0	28.8
PYR 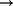 BAS	8.2	7.6	15.0	0.2
BAS 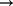 PYR	23.3	39.4	0.5	0.2
RSNP 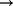 PYR	22.7	39.9	0.0	3.9
L4 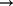 PYR	44.1	45.4	15.5	2.3
background 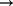 PYR	32.3	31.3	17.3	1.3
total	22.2	25.2	17.9	9.8

Projection-wise synapse loss in % for the default (9HC×9MC) and large-scale (25HC×25MC) network. See text for the respective differences between the distorted (dist.) and compensated (comp.) networks.

*Large-scale model.* The ability of the network to perform pattern completion and exhibit pattern rivalry was tested on the ESS for the large-scale model with 25 HCs and 25 MCs per HC. From the start, we use a background pool with 5000 Poisson sources and 100 sources per neuron to model the diffusive background noise, as used for the compensation of synaptic weight noise (Section 3.1.5). As with the small-scale network, the synapse loss of 17.9% shows significant heterogeneity (Table 1), and affects mainly projections from PYR to inhibitory cells, but also connections from the background and L4 stimulus. In contrast to the idealized case in Section 3.1.4, where each synapse is deleted with a given probability, the synapse loss here happens for entire projections at the same time, i.e. all synapses between two populations are either realized completely or not at all. We note that the realization of all PYR-RSNP synapses is a priori impossible, as each RSNP cell has 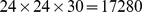 potential pre-synaptic neurons (cf. scaling rules in section S2.4 of Appendix S2), which is more than the maximum possible number of pre-synaptic neurons per HICANN (14336, see Section 2.1.1). The simulation results with 20% synaptic weight noise for pattern completion and pattern rivalry are shown in Figure 12 **K** and **L** (red curves). In both cases the network functionality is clearly impaired. In particular, the ability of an active pattern to suppress other patterns is noticeably detoriated, which can be traced back to the loss of 37% of PYR-RNSP connections.

In order to restore the functionality of the network we used a two-fold approach: First, we attempted to reduce the binary loss of PYR-RSNP projections by reducing the number of PYR cells per MC from 30 to 20, which decreases the total number of neurons in the network, as well as the number of potential pre-synaptic neurons per RNSP cell. The synapse loss was thereby reduced to 28.8% for PYR-RSNP projections and was eliminated almost completely for all other projections (Table 1). Secondly, we compensated for the remaining synapse loss by scaling the synaptic weights as described in Section 3.1.4.

After application of these compensation mechanisms, we were able to effectively restore the original functionality of the network. Both pattern completion and attentional blink can be clearly observed. The small remaining deviations from the default model can be attributed to the inhomogeneity of the synapse loss and the fixed delays on the wafer.

### 3.2 Synfire Chain with Feed-Forward Inhibition

Our second benchmark network is a model of a series of consecutive neuron groups with feed-forward inhibition, called *synfire chain* from here on [70]. This network acts as a selective filter to a synchronous spike packet that is applied to the first neuron group of the chain. The behavior of the network is quantified by the dependence of the filter properties on the strength and temporal width of the initial pulse. Our simulations show that synapse loss can be compensated in a straightforward manner. Further, the major impact of weight noise on the network functionality stems from weight variations in background synapses, which can be countered by modification of synaptic and neuronal parameters. The effect of fixed axonal delays on the filtering properties of the network can be countered only to a limited extent by modifying synaptic time constants and the strength of local inhibition. Simulations using the ESS show that the developed compensation methods are applicable simultaneously. Furthermore, they highlight some further sources of potential failure of pulse propagation that originate from bandwidth limitations in the off-wafer communication infrastructure.

#### 3.2.1 Architecture

Feed-forward networks with a convergent-divergent connection scheme provide an ideal substrate for the investigation of activity transport. Insights have been gained regarding the influence of network characteristics on its response to different types of stimulus [71]–[73]. Similar networks were also considered as computational entities rather than purely as a medium for information transport [74]–[76]. The behavior of this particular network has been shown to depend on the connection density between consecutive groups, on the balance of excitation and inhibition as well as on the presence and magnitude of axonal delays in [70]. This makes it sensitive to hardware-specific effects such as an incomplete mapping of synaptic connectivity, the variation of synaptic weights, bandwidth limitations which cause loss of individual spike events and limited availability of adjustable axonal delays and jitter in the spike timing that may be introduced by different hardware components.

The feed-forward network comprises a series of successive neuron groups, each group containing one excitatory and one inhibitory population. The excitatory population consists of 100 regular-spiking (RS), the inhibitory of 25 fast-spiking (FS) cells. Both cell types are modeled as LIF neurons with exponentially shaped synaptic conductance without adaptation, as described in section 2.1.1. Both RS and FS neurons are parameterized using identical values (Table S3.1 in Appendix S3).

**Figure 13:**
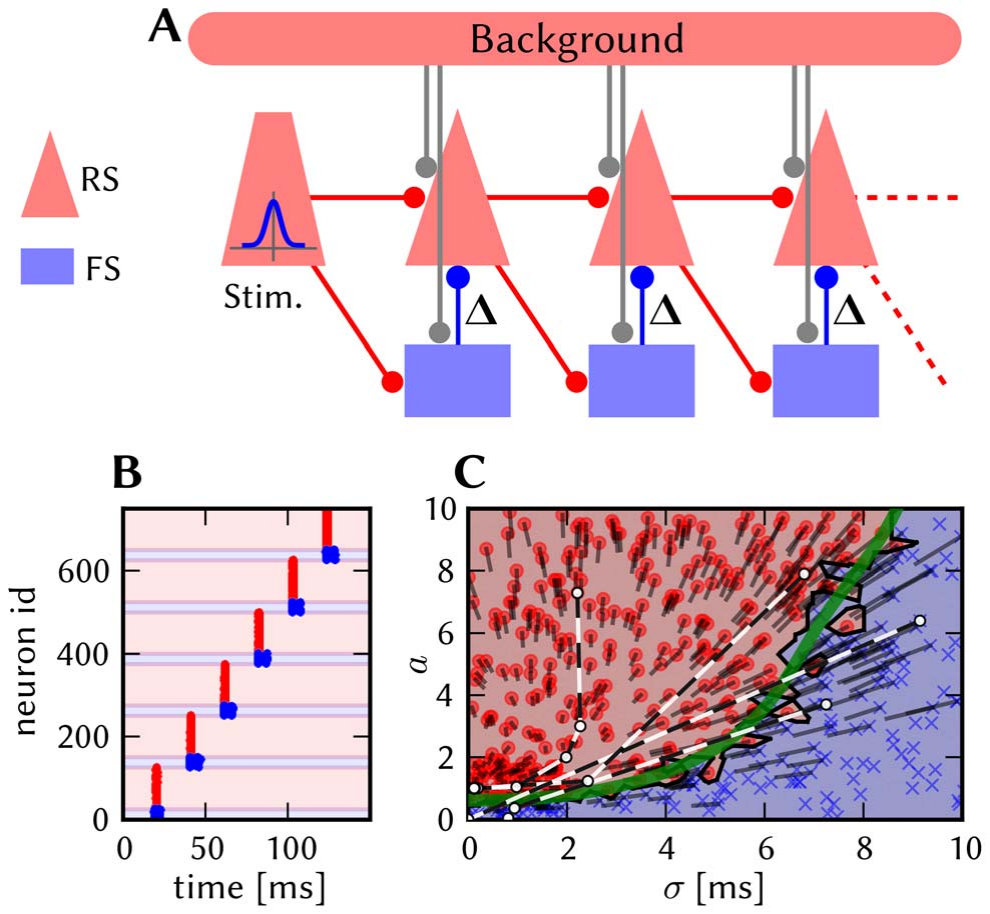
Synfire chain network. (A) Connectivity of the synfire chain with feed-forward inhibition [70]. Excitatory projections are shown in red, inhibitory in blue. In the default realization the network consists of six consecutive groups. The local FS 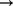 RS projection has an adjustable delay 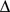, which affects the network dynamics. The intergroup delay is set to 20 ms for visualization purposes following the previous work; this has no influence on the filter properties because the delay of both intergroup projections is equal. The background stimulus is realized using random Gaussian current (original) and Poisson background spikes (adapted version for the hardware). The parameters for neurons and connections are given in Table S3.1 and Table S3.2 in Appendix S3. (B) Exemplary raster plot of the network behavior. The first group receives a pulse packet with 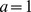 and 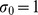
ms, which propagates as a synchronous spike volley along the chain. (C) Characterization of the network behavior in the 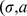) state space. Each marker represents the initial stimulus parameters 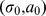. The stimulus parameters were selected randomly from the region (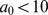, 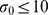
ms). The region with (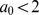, 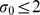
ms) was simulated more frequently to increase the resolution near the convergence points of the propagation. The marker color is linearly scaled with the activity in the last group, 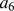, being blue for 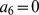 and red for 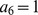 and is set to red for 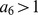. To improve visibility, the background is colored according to the color of the nearest marker, red for 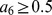 and blue otherwise. Experiments where the RS group did not fire are marked as 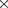. The gray lines originating from each marker denote the direction towards the pulse volley parameters 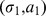. The green line shows a fit to the separatrix between zero and nonzero activity at the last group of the synfire chain (see section 3.2.2 for details). This approximation is used to compare the behavior of different modifications of the original network. The dashed black and white lines show four exemplary trajectories through the 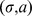 state space.

Each excitatory population projects to both populations of the consecutive group while the inhibitory population projects to the excitatory population in its local group (Figure 13 **A**). There are no recurrent connections within the RS or FS populations. In the original publication [70], each neuron was stimulated independently by a Gaussian noise current. Because the hardware system does not offer current stimulus for all neurons, all neurons in the network received stimulus from independent Poisson spike sources. For Gaussian current stimulus, as well as in the diffusion limit of Poisson stimulus (high input rates, low synaptic weights), the membrane potential is stationary Gaussian, with an autocorrelation dominated by the membrane time constant. The only remaining differences are due to the finite, but small, synaptic time constants. The rate and synaptic weight of the background stimulus were adjusted to obtain similar values for the mean and variance of the membrane potential, resulting in a firing rate of 2 kHz with a synaptic weight of 1 nS.

The initial synchronous stimulus pulse is emitted by a population of spike sources, which has the same size and connection properties as a single RS population within the network. A temporally localized *pulse packet* was used as a stimulus, whereby each of the 100 spike sources emitted 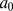 spikes that were sampled from a Gaussian distribution with a common mean time and a given standard deviation 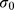. The variables 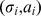 are later used to describe the characteristics of the activity in the *i*th group of the chain, referring to the temporal pulse width and number of spike pulses per neuron, respectively.

#### 3.2.2 Functionality criteria

**Figure 14:**
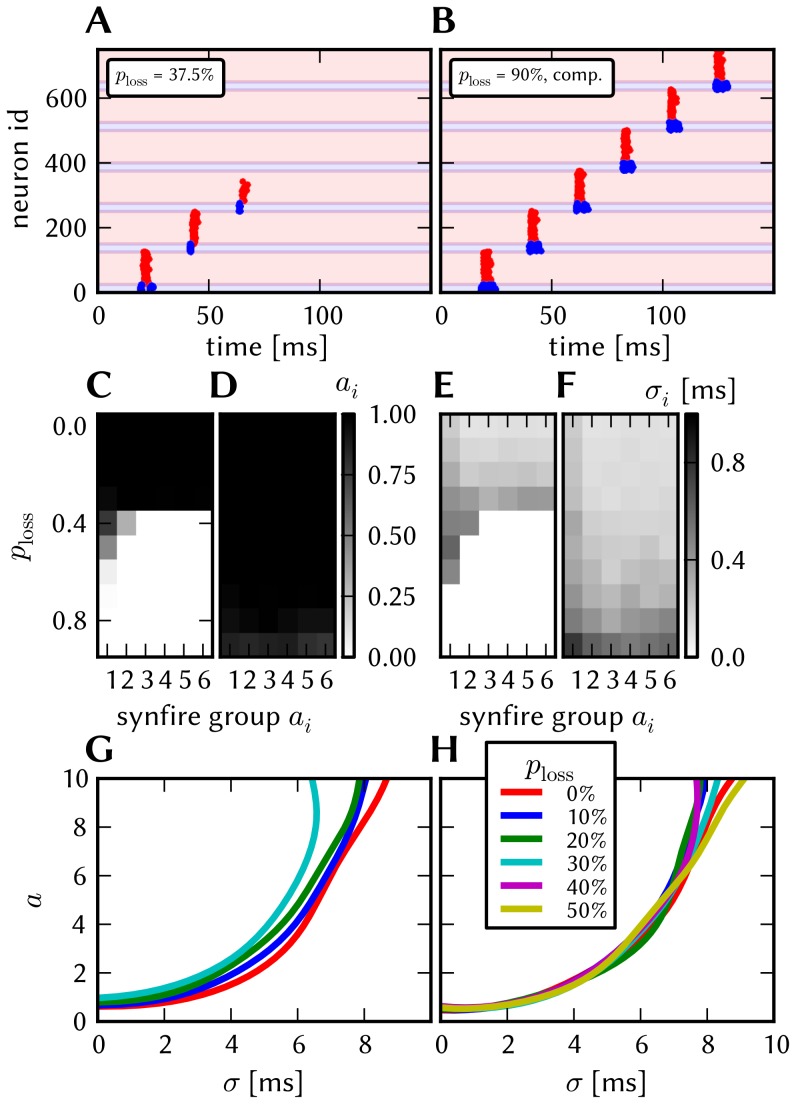
Effect and compensation of synapse loss for the synfire chain network. (A) Synfire network with 37.5% synapse loss applied to all connections within the network. External connections (synchronous stimulus and background) are not affected. (B) Raster plot with active compensation at 90% synapse loss. (C) Activation 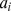 in each group 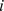 with varying values of synapse loss. (D) 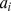 as in C but with active compensation. (E) Pulse width 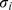 in each group 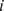 with varying values of synapse loss. (F) Pulse width as in E but with active compensation. (G) Comparison of approximated separatrix locations for synapse loss values from 0% to 50%. The lines for 40% and 50% are missing because no stable region exists. (H) Approximated separatrix locations with active compensation.

The functionality of the feed-forward network is assessed by examining the propagation of a synchronous pulse after the stimulus is applied to the first group in the chain (Figure 13 **B**). The propagation is quantified by applying initial stimuli of varying strength 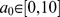 and temporal spread 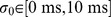. For each synfire group 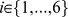, the activation is determined by setting 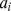 to the number of emitted spikes divided by the number of neurons in the RS population. 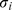 is the standard deviation of the spike pulse times. Typically, the resulting “trajectory” in the 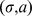 space (Figure 13) is attracted to one of two fixed points: either near 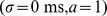, i.e., the pulse packet propagates as a synchronous spike volley, and 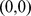, i.e., the propagation dies out (e.g., Figure 14 **A**).

The network behavior is characterized by the separating line between successful and extinguished propagation in the state space 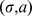 of the initial stimulus; this line will be called *separatrix* from here on. The differentiation between extinguished and successful propagation is defined by 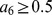 resp. 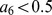 in the last (6th) group. This is justified because in the undistorted case, 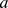 is clustered around the values 0 and 1 (Figure S3.1 in Appendix S3). Due to the statistic nature of the connectivity, background stimulus and pulse packet, the macroscopic parameters 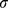 and 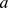 do not fully determine the behavior of the system. This means that in the reference simulation, there is a small region around the separatrix where the probability of a stable pulse propagation is neither close to zero nor to one. Thus, in addition to the location of the separatrix (see section S3.3.2 in Appendix S3), the width of this region is taken as a functionality criterion.

The background stimulus is adjusted such that the spontaneous firing rate in the network is below 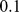 Hz, in accordance with [70]. In cases in which distortion mechanisms induce a much stronger background firing, the spike trains are filtered before the analysis by removing spikes which appear not to be within a spike volley (Section S3.3.4 in Appendix S3).

#### 3.2.3 Synapse loss

Homogeneous synapse loss affects the strength of excitatory and inhibitory projections equally on average. Additionally, the number of incoming spikes seen by a single neuron varies as synapses are removed probabilistically, in contrast to the undistorted model with a fixed number of incoming connections for each neuron type (Table S3.2 in Appendix S3). Synapse loss was applied to all internal connections as well as to the connection from the synchronized stimulus population to the first group in the network; background stimulus was not affected (cf. section 2.4). Figure 14 **A** shows a single experiment with synapse loss of 37.5%, contrasting with the undistorted case (Figure 13 **A**). Above a certain value of synapse loss, the signal fails to propagate to the last group. As shown in Figure 14 **C** and **E** for one stimulus parameter set, successful propagation stops at a synapse loss value between 30% and 40%. The pulse width increases with rising synapse loss due to the increasing variation of synaptic conductance for individual neurons (**E**). The effect is reversed by increasing all synaptic weights in the network by a factor of 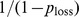, with 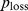 being the probability of synapse loss. This compensation strategy can effectively counter synapse loss of up to 90% (**B**, **D**) and the pulse width increase is shifted to larger values of synapse loss (**F**). The distortion mechanism has only a minor effect on the 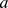-value of the separatrix in the depicted region (**G**). However, the location of the separatrix at 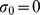 rises with synapse loss until it reaches the fixed point at approx. 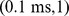, at which point a bifurcation occurs and the the attractor region for 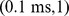 disappears (as described in [77] for the case of varying weights). In the compensated case, the separatrix locations are identical with the undistorted case within the measurement precision.

#### 3.2.4 Synaptic weight noise

**Figure 15:**
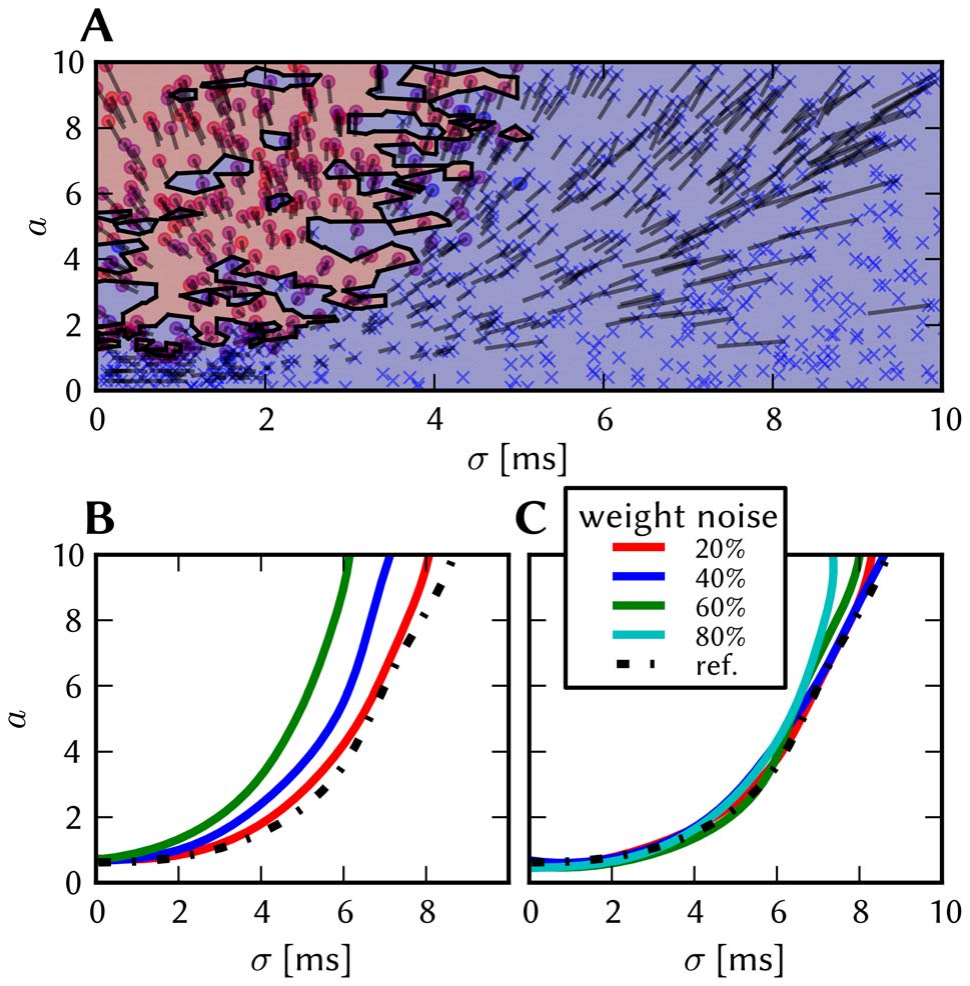
Effect of synaptic weight noise on the synfire chain model. The spike data for all three plots was filtered to remove spontaneous spikes in individual neurons, which stem from weight increase in some background synapses due to weight noise. (The filter parameters were 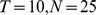, cf. section S3.3.4 in Appendix S3) (A) State space at 80% weight noise. The set of inputs that evokes activity in the last group is patchy as a consequence of the distortion mechanism. In the compensated case the separation is sharp again, as shown in Figure S3.3 of Appendix S3. (B) Approximate separatrix locations for smaller values of weight noise. (C) Approximate separatrix locations for the compensated case.

The effect of synaptic weight noise is shown in Figure 15. Similarly to the effect of synapse loss, the region of stable propagation shrinks (**B**); additionally, the border between the regions of stable and extinguished propagations becomes less sharp (**A**). This is caused by two effects: Varying strength of the background stimulus, and varying strength of the synaptic connections within the network. The first effect is significant because the background stimulus to each neuron is provided through a single synapse. Thus, the effective resting potential of each neuron is shifted, significantly changing its excitability and, in some cases, inducing spontaneous activity. One possibility of countering this effect is to utilize several synapses for background stimulus thereby averaging out the effect of individual strong or weak synapses, as has been done in the case of the L2/3 model in section 3.1.5. Here, a different method was employed: The resting potential 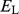 was raised while simultaneously lowering the synaptic weight from the background stimulus. The parameters were chosen in such a way that the mean and variance of the distribution of membrane voltages in each neuron population was kept at the value of the undistorted network:
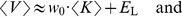
(10)

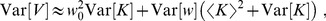
(11)where 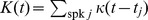 represents the effect of the background stimulus, 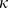 being the PSP kernel, and 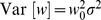 appears due to synaptic weight noise. In the distorted case, the width of this distribution is a combined effect of the random background stimulus and the weight variation, while in the original case it originates from the stochasticity of the stimulus only. In the undistorted case, 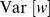 is 0, and only the first term contributes to 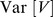. With increasing 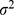, the contribution of the second term to 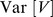 increases, which is compensated by changing 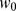 accordingly, keeping 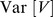 at the original level. This, in turn, changes 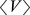, which is compensated by a change of 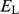.

The effect of synaptic weight noise within the network itself is less significant compared to its impact on the noise stimulus. Figure 15 **C** shows that removing the effect of background stimulus noise alone is sufficient to counteract synaptic noise values of up to 50%.

#### 3.2.5 Non-configurable axonal delays

**Figure 16:**
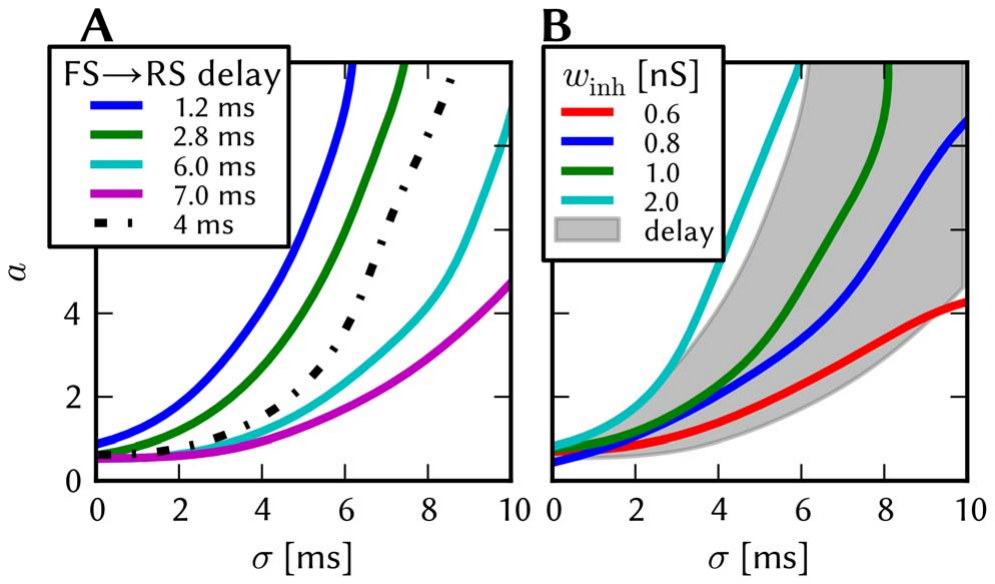
Delays in the synfire chain model. (A) Reproduction of [70], fig. 4c. The location of the separatrix is modified by changing the axonal delay of local inhibition. For a value of 0.4 ms, no stable region is present. (B) The location of the separatrix is modified by varying weights for synapses taking part in local inhibition. The axonal delay of local inhibition was fixed at 1.5 ms and the inhibitory time constant was increased by a factor of 3. The gray region shows the range of the separatrix location for delay values from 1.2 ms to 7 ms (the range in plot A) as reference.

Figure 16 **A** shows the effect of varying axonal delays between the inhibitory and excitatory population of a single synfire group. As was shown in [70], the delay can be employed to control the position of the separatrix between stable and unstable propagation. Because the axonal delay is not configurable for on-wafer connections, a different method is required to regain the ability to control the separatrix. While section 3.2.3 and section 3.2.4 show that synaptic weight noise and synapse loss can influence the location of the separatrix, a method is required that is independent of those distortion mechanisms. [78] shows that several parameters, including group size and noise level, can modify the separatrix location, albeit for a model without feed-forward inhibition. Here, we investigate to which extent parameter modification can reproduce the effect of variable delays. For very short delays (in this case, 0.1 ms, not shown), stable propagation does not occur, because the onset of local inhibition is nearly synchronous with the onset of external excitation. This effect was countered by increasing the synaptic time constant and simultaneously decreasing the synaptic weight for local inhibition, thus extending the duration of inhibition that acts on the RS population. The inhibitory synaptic time constant was increased by a factor of 3 while simultaneously reducing the synaptic weight of the inhibitory projection. Figure 16 **B** shows the result of the compensation for 1.5 ms local inhibition delay. For both values of axonal delay, the location of the separatrix can be controlled by changing the weight of inhibition. However, its shape differs from the delay-induced case because of the modified delay mechanism of inhibition. Reduction of the weight beyond a certain point is not possible, as balanced inhibition is required for network functionality [70]. It is important to note that this kind of compensation is specific to the state space region which is examined, and that it can not be extended to arbitrarily large delays.

#### 3.2.6 Full simulation of combined distortion mechanisms

At last, we simulate the synfire chain with the ESS and compensate simultaneously for all the causes of distortions addressed above. Before running ESS simulations, we have verified the compatibility of the proposed compensation strategies for different distortion mechanisms in software simulations dealing with the simultaneous incidence of synaptic weight noise, synapse loss and non-configurable axonal delays (Section S3.3.1 in Appendix S3). We proceed with a quantification of synapse loss after mapping the synfire chain for different network sizes to the hardware. For the ESS simulations we limit the model to very few hardware resources to artificially generate synapse loss, such that all of the above distortion mechanisms are present. Additional hardware simulations investigating the influence of spike loss and jitter on the network functionality are provided in section S3.3.5 of Appendix S3.

**Figure 17:**
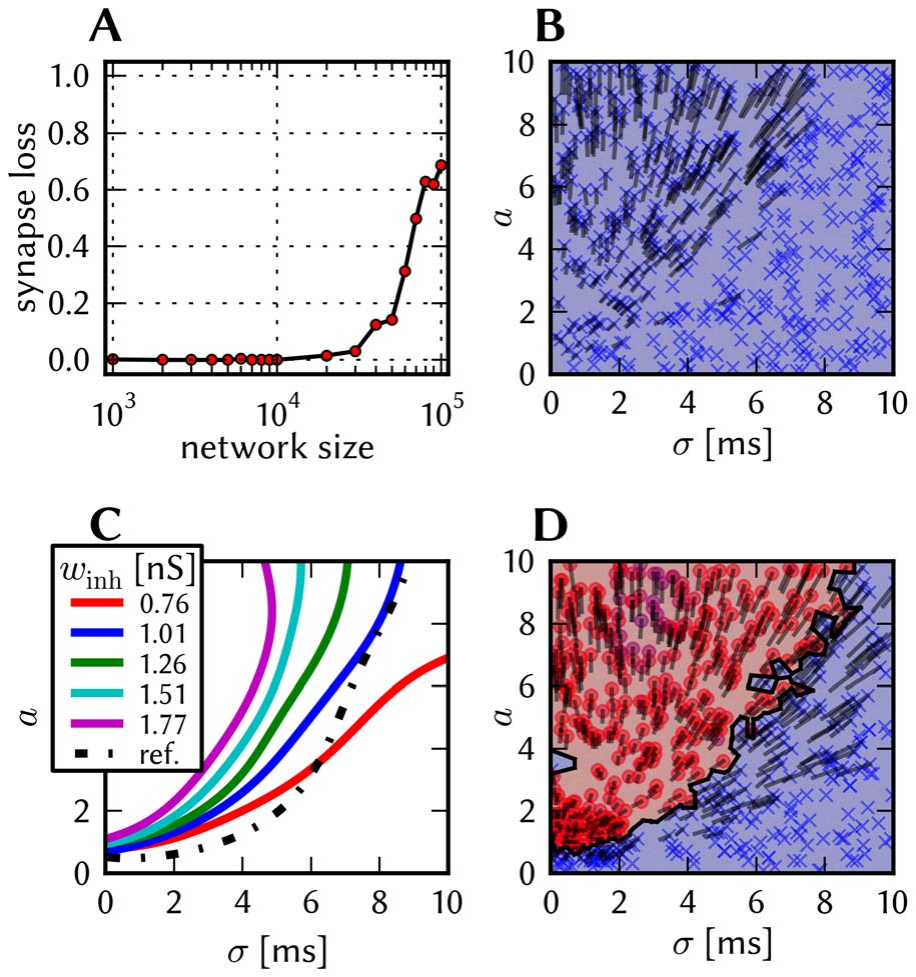
Distorted and compensated simulations of the feedforward synfire chain on the ESS. (A) Synapse loss after mapping the model with different numbers of neurons onto the BrainScaleS System. (B) (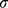,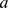) state space on the ESS with default parameters, 20% weight noise, and 27.4% synapse loss. (C) After compensation for all distortion mechanisms, different separatrices are possible by setting different values of the inhibitory weight. (D) Compensated state space belonging to the blue separatrix in C.

*Synapse loss.* We mapped the synfire chain at different network sizes onto the BrainScaleS wafer-scale hardware in order to quantify the synapse loss (Figure 17 **A**). For this purpose we developed network scaling rules that depend on the number and the size of the synfire groups (Section S3.2 in Appendix S3). Due to its modular structure and feed-forward connectivity scheme, there is no synapse loss for networks with up to 10 000 neurons. However, for network sizes above 30 000 neurons, the ratio of lost synapses increases abruptly. With increasing network size more neurons have to be mapped onto one HICANN thereby reducing the number of hardware synapses per neuron. Moreover, as the group size grows with the network size (cf. Table S3.3 in Appendix S3), also the number of pre-synaptic neurons for all neurons mapped onto one HICANN increases, so that the maximum number of inputs to a HICANN, i.e. the synapse drivers, becomes a limiting constraint. The combination of both factors unavoidably leads to synapse loss.

*Distorted and compensated simulation.* For the ESS simulation, we applied the following modifications to the benchmark network: originally, each cell in the network receives Poisson background stimulus from an individual source with 2000 Hz. Because the off-wafer pulse routing network does not support such high bandwidths (cf. section 2.1.2), we reduce the total number of background sources from 750 to 192 and let each neuron receive input from 8 sources, while decreasing the Poisson rate by a factor of 8, using the same mechanism as for the compensation of synaptic weight noise in the L2/3 model (cf. section 3.1.5). For the same reason, the network was emulated with a speedup factor of 5000 compared to biological real-time, whereby the effective bandwidth for stimulation and recording is doubled with respect to the normal operation with a speedup of 10 000. As seen before, no synapse loss occurs for small networks. However, as discussed for the L2/3 model in Section 3.1.7, one can consider situations where only a small part of the wafer is available for experiments, or where some neurons or synaptic elements are defective or missing a calibration. Therefore, in order to generate synapse loss in the feed-forward network, we limited the network to only 8 out of 48 reticles of the wafer and furthermore declare half of the synapse drivers as not available. This resulted in a total synapse loss of 27.4%. As with the L2/3 model, the synapse loss was not homogeneous but depended strongly on the projection type (Table 2).

**Table 2:** Projection-wise synapse loss of the synfire chain model after the mapping process.

projection	synapse loss [%]
Pulse Packet 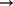 RS_0_	21.3
Pulse Packet 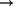 FS_0_	12.7
RS*_n_* 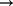 RS*_n_*_+1_	32.4
RS*_n_* 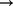 FS*_n_*_+1_	32.0
FS*_n_* 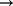 RS*_n_*	20.8
Poisson background 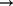 ALL	0
total	27.4

We simulated the synfire chain with default neuron and synapse parameters on the ESS with 20% synaptic weight noise and the above synapse loss. The 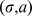 state space (Figure 17 **B**) shows no stable point of propagation. This can be mainly attributed to the small and non-configurable axonal delays which are in the range of 0.6 ms to 1.1 ms for the chosen speedup factor of 5000.

In order to recover the original behavior, we applied the previously developed compensation methods described in Sections 3.2.3 to 3.2.5. Synapse loss was compensated separately for each projection type using Table 2. For synaptic weight noise effectively two compensation methods were applied, as, by using 8 Poisson sources per neuron instead of one, the effect of weight variations is already reduced. Therefore, this fact was considered in the implementation of the second compensation method that scales the synaptic weight and shifts the resting potential 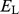 to keep the mean and variance of the membrane voltage constant (Section 3.2.4), by replacing 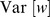 with 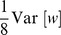 in Equation 11. We were able to compensate for all distortion mechanisms while still maintaining control over the position of the separatrix (Figure 17 **C**).

However, we encountered some abnormalities as can be seen in Figure 17 **D** showing the 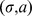 state space for one of the separatrices: For 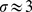 and 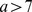 one can recognize a purple region indicating that not all RS cells of the last group spiked. Actually, spikes occurred for all RS cells in the simulated hardware network, but not all spikes were recorded because they were lost in the off-wafer communication network (Section 2.1.2). For very small 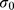 an additional effect can appear: input bandwidth limitations can result in very dense pulse volleys not being propagated through the synfire chain, as can be seen e.g. for the blue point with 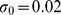
ms and 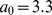 in the left of **D**. In that particular case the large majority of input spikes were lost in the off-wafer communication network so that they did not even reach the first synfire group. We remark that this effect only appeared for 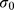 smaller than 0.1 ms.

### 3.3 Self-Sustained Asynchronous Irregular Activity

Our third benchmark is a cortically inspired network with random, distance-dependent connectivity which displays self-sustained asynchronous and irregular firing (short: “AI network”). We define functionality measures on several levels of abstraction, starting from single network observables such as the network firing rate, the correlation coefficient and the coefficient of variation, the properties of the power spectrum of the network activity, up to global behavior such as the dependence of network dynamics on the internal synaptic weights 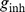 and 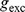. We test two compensation strategies based on a mean field approach and on iterative modification of individual neuron parameters. While the first method offers a way to control the mean firing rate in the presence of synapse loss, the second is applicable to synapse loss and fixed-pattern weight noise simultaneously, in contrast to the other presented compensation methods. Non-configurable axonal delays do not significantly affect the network functionality because the intrinsic hardware delay is approximately equal to the delay utilized in the model. A scaling method for the network size is introduced and the effectivity of the second compensation method was demonstrated using the ESS on a large network with mapping-induced synapse loss and imposed fixed-pattern synapse noise.

#### 3.3.1 Architecture

Self-sustained states in spiking neural networks are known to be exquisitely sensitive to the correlation dynamics generated by recurrent activity [79], [80]. Because of this sensitivity, a model of self-sustained activity within the asynchronous-irregular regime can provide a strong comparison between hardware and software platforms, by requiring the hardware network to reproduce the low firing, weakly correlated, and highly irregular dynamics of this state. Notably, it is often observed that this activity regime provides a good match to the dynamics observed experimentally in the awake, activated cortex [81]–[83]. Additionally, one can note that the self-sustained activity regime provides an interesting test of the BrainScaleS hardware system, as in this state, the model network is not driven by external Poisson input, but has dynamics dominated by internally generated noise [84], beyond the initial brief Poisson stimulation to a small percentage of the network.

The self-sustained regime constitutes an attractor of a dynamical system [85]. Networks based on this principle have been implemented in neuromorphic VLSI hardware [86].

Here, we used a reduced model based on that published in [87]. Neurons in the network followed the AdEx equations 1 to 3 with parameters as in [88], modeling regular spiking pyramidal cells (PY) with spike frequency adaptation [89] and fast spiking inhibitory cells (INH) with relatively little spike frequency adaptation. Instead of explicitly modeling the thalamocortical or corticocortical networks, as in the previous work, we have chosen to modify the model, simplifying it to a single two-dimensional toroidal sheet and adding local connections and conduction delays. The addition of local connectivity follows the experimental observation that horizontal connections in neocortex project, for the most part, to their immediate surroundings [90], while the choice of linear conduction delays reflects electrophysiological estimates of conduction velocity in these unmyelinated horizontal fibers, in the range of 0.1 to 0.5 m s^−1^ [91]–[95]. Propagation delays are known to add richness to the spatiotemporal dynamics of neural network models [96], and in this case are observed to expand the region in the 2D space spanned by the excitatory and inhibitory conductances that supports self-sustained activity, albeit only slightly.

**Figure 18:**
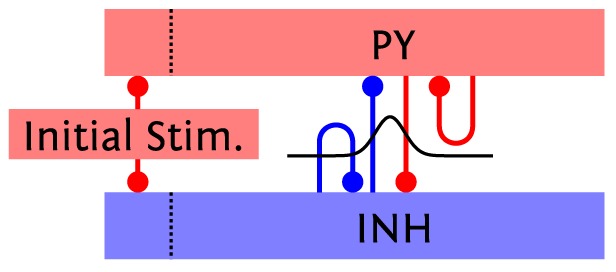
Schematic of the connectivity of the random cortical network. Excitatory PY and inhibitory INH neurons are connected randomly with a spatial, Gaussian connection probability profile. The connection properties are given in Appendix S4. A small part of the network is stimulated in the beginning of the experiment.

Figure 18 shows a schematic of the AI network with its distance-dependent connectivity. A small part of the neurons is stimulated at the beginning of the experiment. Depending on its parameters, the network is able to sustain asynchronous irregular firing activity. The details about the architecture and the parameters used are given in Appendix S4.

#### 3.3.2 Functionality criteria

The global functionality criterion for this network consists of the ability to sustain activity in an asynchronous and irregular activity regime. The activity is considered self-sustained upon persistence to the end of the chosen simulation period. The activity characteristics are quantified for the pyramidal cells using the mean and variance of the firing rates, the irregularity of individual spike trains (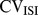, see Equation S4.1 in Appendix S4), the synchrony via the correlation coefficient (CC, Equation S4.2 in Appendix S4) and the power spectrum (see, e.g. 3.1.4 in [97]) of the excitatory activity. The implementation details are given in section S4.2 of Appendix S4.

**Figure 19:**
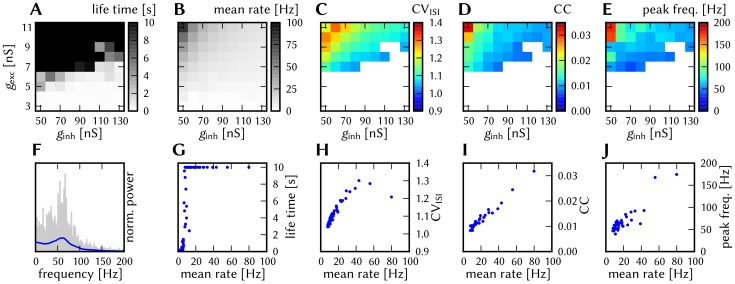
Behavior of the undistorted AI network. On the top: survival time (A), mean firing rate (B), coefficient of variance 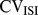 (C), coefficient of correlation CC (D) and position of peak in power spectrum of global activity (E) in the parameter space for 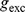 and 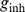 for the default network with 3920 neurons without any distortions. (F) Power spectrum of the global pyramidal activity for the state (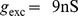, 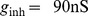). The population activity was binned with a time of 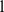
ms, the raw spectrum is shown in gray, the blue curve shows a Gauss-filtered (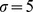
Hz) version for better visualization. The position of the peak in the filtered version was used for (E). In (G–J) the dependence of single criteria on the mean firing rate is shown: survival time (G), 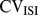 (H), CC (I), position of peak in power spectrum (J). In the last three plots only surviving states of the (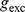, 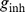) space were considered.

These criteria were evaluated for a range of excitatory and inhibitory synaptic weights 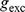 and 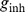 for the default network consisting of 3920 neurons. Figure 19 (**A**) shows the region in the (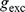, 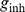) parameter space that allows self-sustained activity, which is achieved at pyramidal firing rates above 8 Hz (**G**).

**Figure 20:**
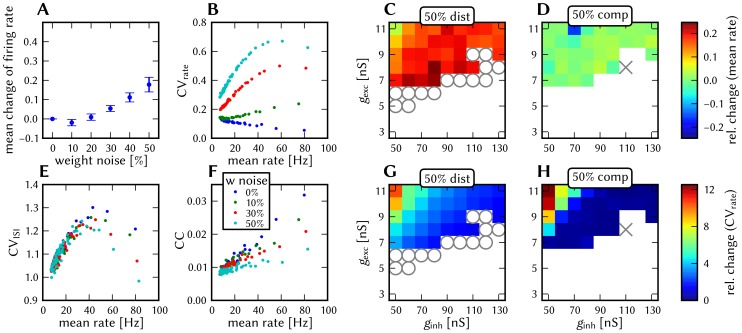
Effect and compensation of synapse weight noise in the AI network. (A) Relative change of the firing rate with respect to the undistorted network averaged over all sustained states for varying synapse weight noise. (B) 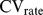 as a function of mean rate for every survived state for varying synapse weight noise. (C and D) Relative change of the firing rate with respect to the undistorted for each state for 50% synapse weight noise(C) and compensated (D). (E) 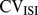 as a function of mean rate for varying synapse weight noise. (F) CC as a function of mean rate for varying synapse weight noise. (G and H) Relative change of 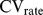 with respect to the undistorted for each state for 50% synapse weight noise(G) and compensated (H). In (C and D) and (G and H): A cross marks a state that was sustained in the undistorted but not sustained in the compared case. A circle marks a state that was not sustained in the original but sustained in the compared case.

The coefficient of variation of the firing rates across neurons (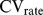) is small (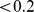, see the 0% weight noise data in Figure 20 **B**), as all neurons have identical numbers of afferent synapses with identical weights in each network realization. In addition to the parameter space plots in the top row of Figure 19, we plot the other criteria against the mean firing rate in the bottom row and recognize the latter as the principal property of each state that mostly determines all other criteria.

The activity is irregular (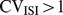) across all states (**C**) and is mainly determined by the network firing rate: the 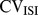 first increases with the firing rate, then saturates and decreases for rates higher than 50 Hz (**H**). Over the entire parameter space, the spike trains of the pyramidal cells are only weakly correlated, with a CC between 0.01 and 0.03.

The average CC increases with the firing rate, which can be attributed to local areas in which neurons synchronize over short time periods. At last, we look at the power spectrum of the global pyramidal activity, exemplarily for the (9 nS, 90 nS) state in (**F**). As a comparison for further studies we follow [82] and use the position of the non-zero peak in the power-spectrum, which is shown for each (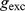, 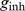) point (**E**) and as a function of the firing rate (**J**): The position of the power spectrum peak frequency (section S4.2 in Appendix S4) increases linearly with the mean firing rate.

#### 3.3.3 Non-configurable axonal delays

For the analysis of the effects of non-configurable delays we repeated the (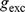, 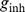) sweep with all axonal delays set to 1.5 ms, cf. section 2.4. This distortion mechanism did not affect any of the functionality criteria, as each neuron still received synaptic input comparable to the reference case. One might expect an influence on the power spectrum of global activity as we switched from distance-dependent delays to a globally constant delay of 1.5 ms as it changes the temporal correlation of the effect of a neuron on all of its efferents. In fact, the power spectra did not change significantly, which can be explained as follows: In the reference case, the *average* of all distance-dependent delays in the network amounts to 1.55 ms (cf. Figure S4.1 in Appendix S4), which is close to the constant delay value of 1.5 ms we use to model the non-configurable delays on the hardware. In this particular case, the hardware delay matches the average delay in the network such that no distortion is introduced. Accordingly, parameter space sweeps on the ESS yielded the same results.

In Appendix S4 (Section S4.4.2) we provide further simulations on the influence of the distribution of delays on the behavior of the network, showing that the effect of the distance-dependent delays is small and that it is mostly the average delay which matters. In our case, this delay exactly corresponds to the average delays on the wafer when running at a speedup of 10 000 compared to biological real-time, such that there is no need for a compensation here.

We note that for variants of this benchmark, where the average network delay is higher or lower than 1.5 ms, there exists a simple but effective compensation strategy by just modifying the speedup of the emulation on the hardware, such that the average network delay is directly mapped onto the hardware delay. We can assume a modified experiment where the average delay amounts to 3 ms. By choosing a speedup of 20 000, this delay can be directly mapped to the 150 ns average delay on the hardware. Such a change of emulation speed is not arbitrary, as one has to make sure that the neural dynamics can still be emulated at the chosen speed (cf. supported parameter ranges in Table S1.1 in Appendix S1). Furthermore, the reduced bandwidth for the pulse communication, especially for external stimulation, must be considered. While this is no issue for this self-sustaining kind of network, these conditions must be also fulfilled for potential other networks that are interconnected to the AI network.

#### 3.3.4 Synaptic weight noise

The effects of synaptic weight noise between 10% and 50% (cf. Section 2.4) on the AI network are shown in Figure 20: The region of self-sustained states in the (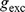, 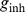) space is increased by this distortion mechanism, cf. the circles in (**C**) marking states that survived with 50% synaptic weight noise but not in the undistorted case. The firing rate increases with the degree of noise (**A**): the change is the stronger the lower 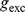 and diminishes for states with an already high firing rate in the undistorted case (**C**). Synaptic weight noise leads to an increase of the variation of firing rates (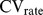), with the change being stronger for high population firing rates (**B**). The 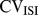 as a function of firing rates remains unchanged for low rates, but decreases for higher firing rates in proportion to the noise level (**E**). Furthermore, weight noise introduces randomness into the network, thereby reducing synchrony: The pairwise correlation between neurons decreases linearly with the amount of weight noise (**F**). The power spectrum of the global activity is not affected by this distortion mechanism.

#### 3.3.5 Synapse loss

**Figure 21:**
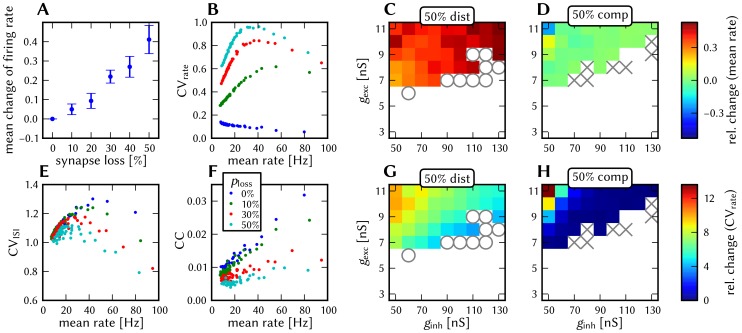
Effect and compensation of synapse loss in the AI network. (A) Relative change of the firing rate with respect to the undistorted network averaged over all sustained states for varying synapse loss. (B) 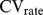 as a function of mean rate for every survived state for varying synapse loss. (C, D) Relative change of the firing rate with respect to the undistorted case for each state for 50% synapse loss (C) and compensated (D). (E) 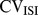 as a function of mean rate for varying synapse loss. (F) CC as a function of mean rate for varying synapse loss. (G, H) Relative change of 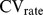 with respect to the undistorted case for each state for 50% synapse loss (G) and compensated (H). In C, D, G and H: A cross marks a state that was sustained in the undistorted but not sustained in the compared case. A circle marks a state, that was not sustained in the original but sustained in the compared case.

Synapse loss has a similar influence on the network behavior as synaptic weight noise: Figure 21 shows the results of the 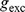-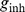 sweeps for synapse loss values between 10% and 50% (cf. Section 2.4). The region of sustained states increases with synapse loss but not as strongly as for weight noise (**C**). The firing rate increases with synapse loss (**A**): Compared to the change caused by synaptic weight noise, however, the effect is much stronger for synapse loss. The same holds for the variance of the firing rates across the pyramidal neurons, which again increases with synapse loss, as can be seen in (**B**). Note that the 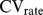 first increases with the mean rate, then reaches a maximum and finally saddles for high rates. We remark that for high synapse loss, some neurons did not fire at all. Both the irregularity and the correlation of firing decrease with increasing synapse loss, leaving the network still in an asynchronous irregular state (**E** and **F**). Synapse loss shows no effect on the power spectrum of global pyramidal activity.

#### 3.3.6 Compensation strategies

The hardware-induced distortions on the AI network analyzed in the previous sections leave two major criteria that need to be recovered: The population firing rate and the variation of firing rates across the population. We consider the other effects (change of CC, 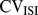, peak frequency in power spectrum) as minor because they are mainly determined by the mean rate and discard them in the following.

One apparent approach for recovering the original firing rate is to change the strengths of the synaptic weights 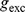 and 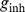. Considering the conducted (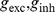) parameter space sweeps, we could simply select the distorted state that best matches the criteria of the undistorted reference. However, this method requires to scan 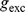 and 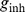 over a wide range to finally get to the desired result. Preferably, one wants to have a compensation method that can be applied to a single experiment and works without huge parameter sweeps.

*Mean field compensation for rate change.* The mean firing rate in the network rises with an increasing synapse loss value. This effect can be understood using a mean-field approach (see, e.g. [79]) in which the response rate of a single neuron's firing rate is assumed to be a function of the mean network firing rate.
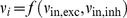
(12)


With this ansatz, which is similar to the approach in [82] where the afferent neurons are replaced by independent Poisson processes with equal instantaneous rate in a sparse random network, the mean firing rate in a self-sustained state can be calculated as a stable, self-consistent solution of the gain function being equal to the firing rate of a single neuron:
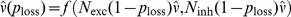
(13)


**Figure 22:**
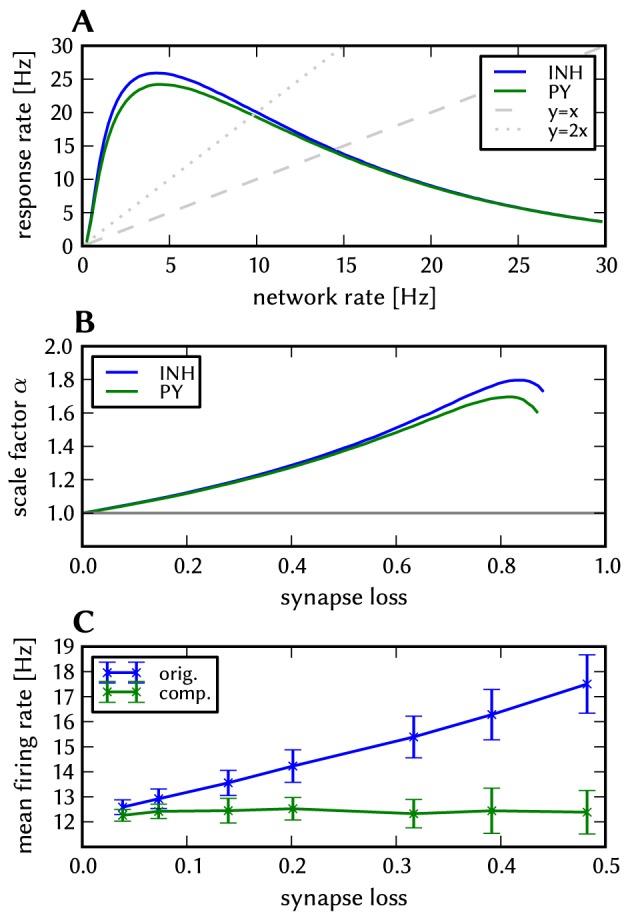
Mean-field-based compensation method for the AI network. (A) Mean firing rate of a single PY and INH neuron given a poisson stimulus by the external network with a given rate. (B) Compensation factor 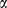 calculated from the data in A. (C) Compensation applied to the self-sustained network (with parameters 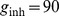
nS, 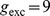
nS). The error bars denote the standard deviation of mean firing rates across all neurons. “orig.” marks the original network without compensation, in “comp.” the neuron parameters were modified according to the compensation factor. The scaling of internal delays had only minimal effect on the firing rate (not shown).

Here, 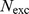 and 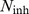 are the number of pre-synaptic connections of a given neuron, and 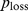 is the modeled synapse loss value. Figure 22 **A** shows the gain function (right-hand side of Equation 13) of PY and INH neurons for 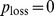 yielding the stable solution 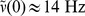 as the intersection of the 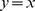 diagonal and the gain function. Analogously, the solution for 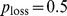 can be determined as the intersection with the 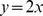 line (considering 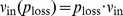). The result justifies the assumption of the mean firing rate of inhibitory and excitatory neurons being equal for 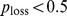.

The parameter change that is necessary to restore the original mean firing rate can be calculated using the following relationship for the time scaling of the solution of a differential equation:
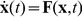
(14)

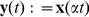
(15)

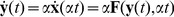
(16)

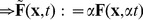
(17)


Assuming that 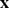 is the state of the dynamic variables within a network, 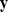 describes a network which follows the same time dependence with the dynamics scaled by the factor 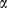 in time. As the given random cortical network shows self-sustained behavior, the transition from 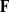 to 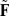 requires only the modification of internal network parameters, because there is no external input (which would have to also be modified otherwise). In particular, the transition encompasses scaling 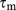, 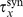, 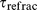, 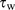 and the synaptic delays by 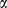, while leaving the conductance jump after each presynaptic PSP unchanged. 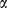 is calculated from the measured gain function (cf. Figure 22) via
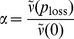
(18)


The resulting firing rate with and without compensation is shown in Figure 22 **C**. The results also show that the variance of the firing rates across neurons grows with rising synapse loss due to the increasing difference in connectivity within the networks. An extension of the mean-field-based compensation to this kind of inhomogeneous connectivity would be impractical, as it requires knowledge of the actual network realization (which is available only after the mapping step) and the measurement of Figure 22 **A** for all occuring counts of presynaptic inhibitory and excitatory neurons. Thus, a different method is considered in section 3.3.6.

In conclusion, this method can be applied when the actual synapse loss value and the mean response function of a single neuron is known. It only depends on the single neuron response properties; the amount of synapse loss has to be known a priori, but not the complete network dynamics. The method depends on the ability to modify synaptic delays according to the scaling rule. However, for the given network, this scaling has only a minimal effect on the mean firing rate.

*Iterative compensation.* The iterative compensation method aims at reducing two distortion effects: the change of the mean firing rate of the pyramidal neurons and its variance across neurons, which are both apparent for synapse loss and synaptic weight noise. It relies on the controlability of the hardware neuron parameters allowing to fine tune the AdEx parameters for every individual neuron (Section 2.1.1). The iterative compensation functions as follows: We start with the results of the reference and the distorted network. From the reference simulation we extract the target mean rate 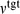 of the neurons in a population. For each neuron in the distorted network, we compare its actual firing rate against 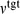, and modify the excitability of the neuron in proportion to the difference between target and measured firing rate. The distorted network with modified neuron parameters is then simulated and the output is compared again to the reference network. This iterative compensation step is repeated until the characteristics of the last step approximately match those of the reference simulation. In our simulations, we modified the spike initiation threshold 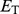, with its change 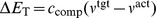 being proportional to the difference between the actual and the target rate. We found that, when choosing the compensation factor 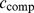 appropriately, 10 iterations are sufficient to restore the mean and variance of the firing rates in the undistorted network. While the compensated mean rate exactly corresponds to 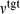, the compensated 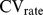 is higher than in the reference network, but reliably below the 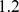-fold of the reference value. The iterative compensation applied in the following is described in detail in section S4.3 of Appendix S4. We remark that the proposed iterative compensation requires a controllable, deterministic mapping, which guarantees that in each iteration the neurons and synapses are always mapped onto the same hardware elements. Furthermore, the complete compensation process needs to be repeated for each network instance. In fact, we perform a calibration of the apparent permanent causes of distortion (fixed-pattern noise and synapse loss) similar to [98] in order to reduce their effects. Hence, whenever we change the random seed that is used to generate the probabilistic connectivity between the neurons, the iterative compensation needs to be run anew. Thus, a reference from a non-distorted simulation or, e.g., from theory is needed. However, once obtained, the result of the compensation can be used for long-running simulations or as part of a larger compound network.

#### 3.3.7 Results of iterative compensation

*Synaptic weight noise.* In order to verify the iterative compensation strategy we applied it to the distorted parameter space with 50% synaptic weight noise. Note that, here and in section 3.3.8, weight noise was implemented persistently, being always the same in all iterations, representing the case where fixed-pattern noise, and not trial-to-trial variability, determines the synaptic weight noise (cf. section 2.4). Accordingly, the following findings are not applicable to the opposite case. The results of the iterative compensation are shown in Figure 20, which displays the relative difference of the mean and variance of the firing rates with respect to the reference simulation in **D** and **H**. The region of sustained activity in the (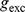, 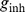) parameter space of the compensated network matches the one of the reference simulation very well. The mean and variance of firing rates could be successfully recovered for most of the states; with the exception of states with a mean rate higher than 25 Hz, where both criteria still differ notably from the reference after 10 iterations (upper left regions in the parameter spaces). We expect that the performance of the iterative compensation for those states could be further improved by tuning the compensation factor 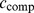 (Section S4.3 in Appendix S4) for high firing rates. The other criteria such as 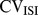 and peak frequency could be fully recovered, following the assumption made earlier, that those criteria mainly depend on the firing rate. However, the coefficient of pairwise cross-correlation (CC) of the compensated networks is lower than in the reference simulation, i.e., the randomness introduced by the synaptic weight noise is still effective.

*Synapse loss.* The results of the application of the iterative compensation strategy to the (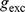, 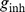) parameter space with 50% synapse loss are shown in Figure 21 (**D** and **H**), displaying the relative difference of mean and variance of firing rates. The compensation was not as effective as for synaptic weight noise: Some states with a low base firing rate were unstable (marked with a cross), i.e. the network did not survive until the end of simulation. As before, the mean and variance of firing rates can be successfully restored for low and medium base firing rates. Again, for high firing rates, the iterative compensation only performed moderately (upper left regions in the parameter spaces **D** and **H**). The other criteria show the same behavior as in the weight noise compensation, i.e. the peak frequency and 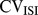 are in good match with the reference while the pairwise correlation (CC) decreased due to the randomness introduced by the synapse loss. We repeated the iterative compensation for the parameter space with 30% synapse loss: The results (not shown) are comparable to the 50% case, but exhibit fewer unstable states, i.e., there were more combinations of 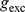 and 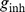 whose compensated network survived.

*Conclusion.* We conclude that the iterative compensation of distorted networks works for both synapse loss and fixed-pattern synaptic weight noise. The compensation also works when both are present at the same time, see section S4.4.3 in Appendix S4 for details. While there seems to be no limit for weight noise, compensation of synapse-loss induced distortions is only possible up to a certain degree, as the network tends to become less stable with fewer synapses involved.

#### 3.3.8 Full simulation of combined distortion mechanisms

In a last step the iterative compensation method designed for the AI network was tested in ESS simulations. Like for the other two models we forced distortions to test the developed compensation strategies. Therefore, we scaled up the network such that a significant fraction of synapses was lost during the mapping process. This large-scale network was then emulated on the ESS and compared to the undistorted reference simulation with NEST. Afterwards, we applied the compensation strategy developed in the previous section to restore the original behavior of the AI network.

**Figure 23:**
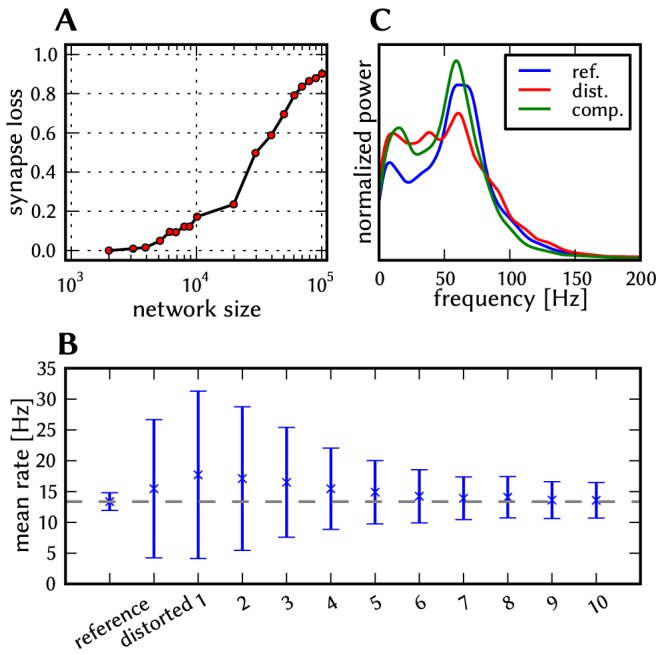
AI network on the ESS. (A) Synapse loss after mapping the network with different sizes onto the BrainScaleS system (B) Iterative compensation of the large-scale network with 22 445 neurons on the ESS: evolution of mean and standard deviation of firing rates for 10 iterations (C) Gauss-filtered power spectrum of global activity of the pyramidal neurons in the large-scale network. Reference spectrum shown in blue (simulated with Nest), distorted and compensated spectra in red resp. green, both simulated with the ESS.

*Synapse loss.* Mapping such homogeneous networks that lack any modularity represents the worst-case scenario for the mapping process, as they have little room for optimization. In Figure 23 **A** the relative synapse loss is plotted for various network sizes using the scaling method described in section S4.1.2 of Appendix S4. One can see that already for low numbers of neurons some synapse loss occurs, although there are sufficient hardware synapses and synapse drivers: due to the sparseness of the on-wafer routing switches some routing buses don't find a free switch to connect to its respective target HICANNs, such that synapses are lost. A kink in the graph of the synapse loss can be seen at around 20 000 neurons, where at least 64 neurons are mapped onto one HICANN (cf. Table S1.2 in Appendix S1). In such a network with random connectivity it is merely possible to find 64 neurons whose pool of pre-synaptic neurons is smaller than 14 336, which is the maximum number of pre-synaptic neurons per HICANN, such that synapse loss must occur. Recall that there is a maximum of 14 336 pre-synaptic neurons for all neurons mapped onto one HICANN. As the connectivity in the AI network is probabilistic, the chance to find groups of 64 neurons whose pool of pre-synaptic neurons is smaller than 14 336 is close to zero.

*Large-scale network.* In order to produce a demanding scenario, we scaled the model to a size of 22 445 neurons (Section S4.1.2 in Appendix S4). The size was chosen such that the network almost occupies an entire wafer, while mapping up to 64 neurons onto one HICANN. This large-scale network has a total of approximately 5.6 million synapses. The statistics of the reference simulation can be found in Table 3 and are in accordance with the scaling behavior investigated in Appendix S4 (Section S4.4.1).

**Table 3:** Statistics of the large-scale AI network.

criteria	ref.	dist.	comp.
Rate [Hz]	13.4	15.5	13.6
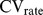	0.107	0.726	0.212
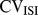	1.12	1.11	1.09
CC	0.00103	0.0011	0.00166
Peak Frequency[Hz]	60.3	60.7	59.0

Reference (ref.) simulated with NEST, distorted (dist.) and compensated (comp.) with the ESS.

*Distorted network.* In the above scenario, 28.1% of synapses were lost during the mapping process (for projection-wise numbers see Table 4). We remark that the synapse loss at this size is higher than during the synapse loss sweep in Figure 23 **A**, as we used a sequence of mapping algorithms that guarantees a balance between synapse loss of excitatory and inhibitory connections. Still, there were slightly more inhibitory connections lost than excitatory ones (Table 4). Additionally, we applied a fixed-pattern noise of 20% to the synaptic weights in the ESS simulation. The result of the latter can be found in Table 3: the network still survived until the end of the simulation, but the firing rate and its variance increased compared to the reference simulation, which complies with the prediction of the distortion analysis.

**Table 4:** Projection-wise synapse loss of the large-scale AI network after the mapping process.

projection	synapse loss [%]
PY 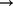 PY	26.9
PY 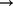 INH	28.1
INH 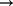 PY	31.1
INH 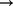 INH	33.4
STIM 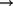 PY	77.5
STIM 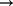 INH	89.4
total	28.1

PY: excitatory pyramidal neurons. INH: fast spiking inhibitory cells, STIM: external Poisson sources for initial stimulation.

*Compensated network.* We then used the iterative compensation method from Section 3.3.6 to compensate the abovementioned distortions and repeated the ESS simulation with the modified network. The evolution of the firing rates over 10 iterations is shown in Figure 23 **B**: One can clearly see how, step by step, the firing rate approaches the target rate and that at the same time the variance of firing rates decreases. The statistics of the final iteration are listed in Table 3: It was possible to fully recover the target mean rate. The variation of firing across neurons (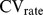) was significantly reduced from 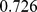 to 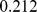 but was still twice as large as in the reference network. The other functionality criteria match the reference simulation very well (Table 3), as does the power spectrum of global activity in Figure 23 **C**.

## Conclusions

In this study, we have presented a systematic comparison between neural network simulations carried out with ideal software models and a specific implementation of a neuromorphic computing system. The results for the neuromorphic system were obtained with a detailed simulation of the hardware architecture. The core concept is, essentially, a functionalist one: neural networks are defined in terms of functional measures on muliple scales, from individual neuron behavior up to network dynamics. The various neuron and synapse parameters are then tuned to achieve the target performance in terms of these measures.

The comparison was based on three cortically inspired benchmark networks: a layer 2/3 columnar architecture, a model of a synfire chain with feed-forward inhibition and a random network with self-sustained, irregular firing activity. We have chosen these specific network architectures for two reasons. First of all, they implement very different, but widely acknowledged computational paradigms and activity regimes found in neocortex: winner-take-all modules, spike-correlation-based computation, self-sustained activity and asynchronous irregular firing. Secondly, due to their diverse properties and structure, they pose an array of challenges for their hardware emulation, being affected differently by the studied hardware-specific distortion mechanisms.

All three networks were exposed to the same set of hardware constraints and a detailed comparison with the ideal software model was carried out. The agreement was quantified by looking at several chosen microscopic and and macroscopic observables on both the cell and network level, which we dubbed “functionality criteria”. These criteria were chosen individually for each network and were aimed at covering all of the relevant aspects discussed in the original studies of the chosen models.

Several hardware constraint categories have been studied: the dynamics of the embedded neuron and synapse models, limited parameter ranges, synapse loss due to limited hardware resources, synaptic weight noise due to fixed-pattern and trial-to-trial variations, and the lack of configurable axonal delays. The final three effects were studied in most detail, as they are expected to affect essentially every hardware-emulated model. The investigated distortion mechanisms were studied both individually, as well as combined, similarly to the way they would occur on a real hardware substrate. As expected, above certain magnitudes of the hardware-specific distortion mechanisms, substantial deviations of the functionality criteria were observed.

For each of the three network models and for each type of distortion mechanism, several compensation strategies were discussed, with the goal of tuning the hardware implementation towards maximum agreement with the ideal software model. With the proposed compensation strategies, we have shown that it is possible to considerably reduce, and in some cases even eliminate the effects of the hardware-induced distortions. We therefore regard this study as an exemplary workflow and a toolbox for neuromorphic modelers, from which they can pick the most suitable strategy and eventually tune it towards their particular needs.

In addition to the investigated mechanisms, several other sources of distortions are routinely observed on neuromorphic hardware. A (certainly not exhaustive) list might include mismatch of neuron and synapse parameters, shared parameter values (i.e., not individually configurable for each neuron or synapse) or limited parameter programming resolution. These mechanisms are highly back-end-specific and therefore difficult to generalize. However, although they are likely to pose individual challenges by themselves, some of their ultimate effects on the target network functionality can be alleviated with the compensation strategies proposed here.

Our proposed strategies aim at neuromorphic implementations that compete in terms of network functionality with conventional computers but offer major potential advantages in terms of power comsumption, simulation speed and fault tolerance of the used hardware components. If implemented successfully, such neuromorphic systems would serve as fast and efficient simulation engines for computational neuroscience. Their potential advantages would then more than make up for the overhead imposed by the requirement of compensation.

From this point of view, hardware-induced distortions are considered a nuisance, as they hinder precise and reproducible computation. In an alternative approach, one might consider the performance of the system itself at some computational task as the “fitness function” to be maximized. In this context, some particular architecture of an embedded model, together with an associated target behavior, would then become less relevant. Instead, one would design the network structure specifically for the neuromorphic substrate or include training algorithms that are suitable for such an inherently imperfect back-end. The use of particular, “ideal” software models as benchmarks might then given up altogether in favor of a more hardware-oriented, stand-alone approach. Here, too, the proposed compensation strategies can be actively embedded in the design of the models or their training algorithms.

The hardware architecture used for our studies is, indeed, suited for both approaches. It will be an important aspect of future research with neuromorphic systems to develop procedures that tolerate or even actively embrace the temporal and spatial imperfections inherent to all electronic circuits. These questions need to be addressed by both model and hardware developers, in a common effort to determine which architectural aspects are important for the studied computational problems, both from a biological and a machine learning perspective.

## Supporting Information

Appendix S1 **Neuromorphic hardware.** Details about the short-term plasticity mechanism, parameter ranges, and parameter variation measurements of the BrainScales neuromorphic hardware system.
(PDF)

Appendix S2 **Cortical layer 2/3 attractor memory.** Methods, model parameters, and detailed simulation results of the Layer 2/3 model.
(PDF)

Appendix S3 **Synfire chain with feed-forward inhibition.** Model parameters and additional simulations of the synfire chain network.
(PDF)

Appendix S4 **Self-sustained asynchronous irregular activity.** Methods, model parameters, and further simulations of the AI network.
(PDF)
