# Supplementary material for: Characterization and Compensation of Network-Level Anomalies in Mixed-Signal Neuromorphic Modeling Platforms
Source: PLoS One. 2014 Oct 10;9(10):e108590. doi: 10.1371/journal.pone.0108590 (PMC4193761; doi:10.1371/journal.pone.0108590)
Supplement: Appendix S1 — Neuromorphic hardware. Details about the short-term plasticity mechanism, parameter ranges, and parameter variation measurements of the BrainScales neuromorphic hardware system. (PDF) [file pone.0108590.s001.pdf]

Appendix S1 Neuromorphic hardware

S1.1 Short-term plasticity

As mentioned in section 2.1.1, the hardware short-term plasticity mechanism is an implementation of the phenomenological model by [44]. We first describe the hardware STP model and then provide the translation between the original and the hardware model.

Model description Unlike the theoretical model [44], which allows the occurrence of both depression and facilitation at the same time, the hardware implementation does not allow their simultaneous activation. The ongoing pre-synaptic activity is tracked with a time-varying active partition I with $0 \leq I \leq 1$, which decays exponentially to zero with time constant τ_{stdf}. Following a pre-synaptic spike, I is increased by a fixed fraction $U_{\mathrm{SE}}(1-I)$, resulting in the following dynamics for the active partition:

$$I_{n+1} = [I_n + U_{\mathrm{SE}}(1 - I_n)] \exp\left(-\frac{\Delta t}{\tau_{\mathrm{stdf}}}\right) \quad , \tag{S1.1}$$

with Δt being the time interval between the nth and $(n+1)$st afferent spike.

This active partition can be used to model depressing or facilitating synapses as follows:

$$w_{\mathrm{STP}}^{\mathrm{depression}} = 1 - \lambda \cdot I \tag{S1.2}$$

$$w_{\mathrm{STP}}^{\mathrm{facilitation}} = 1 + \lambda \cdot (I - \beta) \quad . \tag{S1.3}$$

Here, w_{STP}^x corresponds to a multiplicative factor to the static synaptic weight, with λ and β being configurable variables, and x denotes the mode being either depression or facilitation.

According to Equation 8 the n-th effective synaptic weight is then given by

$$w_n^{\mathrm{syn}} = w_{\mathrm{static}} w_{\mathrm{STP}}^x \quad . \tag{S1.4}$$

Due to a technical limitation, the change of synaptic weights by STP can not be larger than the static weight, such that $0 \leq w_{\mathrm{STP}}^x \leq 2$. We refer to [34] for details of the hardware implementation of STP and to [45] for neural network experiments on neuromorphic hardware using this STP model.

Transformation from original model The original model by [44] (Equation 8) can be translated to the hardware model (Equations S1.1 to S1.3) when one of the two time constants (τ_{rec} or τ_{facil}) is equal to zero.

For depression only ($\tau_{\mathrm{facil}} = 0$), the nth synaptic weight is given by (cf. Equation 8):

$$w_n^{\mathrm{syn}} = w_{\mathrm{max}}^{\mathrm{syn}} R_n U \quad . \tag{S1.5}$$

The time course of R can be exactly represented by $(1 - I)$ if the scaling factor λ of the short-term plasticity mechanism is set to 1. Additionally, the static synaptic weight w_{static} has to be adapted such that the applied synaptic weights are equal, giving us the following transformation: $\tau_{\mathrm{stdf}} = \tau_{\mathrm{rec}}$, $U_{\mathrm{SE}} = U$, $\lambda = 1$ and $w_{\mathrm{static}} = w_{\mathrm{max}}^{\mathrm{syn}} U$.

For facilition only ($\tau_{\mathrm{rec}} = 0$), the recovered partition remains fully available all the time ($R = 1 =$ const) and only the utilization varies with time. Thus the nth synaptic weight is given by:

$$w_n^{\mathrm{syn}} = w_{\mathrm{max}}^{\mathrm{syn}} u_n \quad . \tag{S1.6}$$

The time course of u now has to be emulated by the right-hand side of Equation S1.3; more precisely, we use I to represent the course of $u - U$. Additionally we set $U_{\mathrm{SE}} = U$ and $\tau_{\mathrm{stdf}} = \tau_{\mathrm{facil}}$, and level the limits for the synaptic weights. In the original model, u is always between U and 1, while for the hardware model the STP factor is limited to values between 0 and 2 due to technical reasons. By setting $\lambda = 1$ and considering that I is always within 0 and 1, the supplied range for $w_{\mathrm{STP}}^{\mathrm{facilitation}}$ is $[1-\beta, 2-\beta]$. In order to match the range of applied weights of both models, we need to solve the following system of equations:

$$\begin{aligned}(1-\beta)\cdot w_{\mathrm{static}} &= U \cdot w_{\mathrm{max}}^{\mathrm{syn}} \\ (2-\beta)\cdot w_{\mathrm{static}} &= 1 \cdot w_{\mathrm{max}}^{\mathrm{syn}} \quad .\end{aligned}$$

Solving for w_{static} and β yields

$$\begin{aligned}w_{\mathrm{static}} &= (1-U)\cdot w_{\mathrm{max}}^{\mathrm{syn}} \\ \beta &= \frac{1-2U}{1-U} \quad .\end{aligned}$$

S1.2 Parameter ranges

Here, we provide a full list of available parameter ranges for the BSS waferscale platform in Table S1.1. As mentionend in section 2.1.1, one has the choice between two different capacitances in the hardware neuron. The parameter ranges specified in Table S1.1 correspond to the big capacitance (2.6 pF). When using the small capacitance (0.4 pF) some parameter ranges change: the limits of τ_{m} are multiplied by $\frac{0.4}{2.6}$, the ranges for a, b, and the synaptic weight are divided by $\frac{0.4}{2.6}$. The ranges for electric potentials of the AdEx model (E^{spike}, E^{r}, E_{L}, E_{T}, $E^{\mathrm{rev,e}}$ and $E^{\mathrm{rev,i}}$) result from the following transformation from biological to hardware voltages (cf. section 2.2):

$$V_{\mathrm{hardware}} = \alpha_V \cdot V_{\mathrm{bio}} + V_{\mathrm{shift}} \quad , \tag{S1.7}$$

with $\alpha_V = 10$ and $V_{\mathrm{shift}} = 1300\,\mathrm{mV}$.

In Table S1.2 we show how the tradeoff between total neuron number and maximum fan-in per neuron is realized on this device.

S1.3 Parameter Variation Measurements

Figure S1.1 shows variation measurements on HICANN chips. These measurements allow us to estimate the amount of variation that is present in the circuits (Sections 2.1.1 and 2.4).

The measurements are conducted on a single-chip prototype system (plots **A-D**) and on one chip on a prototype wafer system (plots **E** and **F**). Some neurons (on the right-hand-side of the plots) had been previously labeled non-functional and blacklisted, therefore showing no data points. They will also be omitted during system operation. Additionally, neurons that exhibit a larger variation than a chosen threshold can be blacklisted as well, reducing the total number of available neurons, but also limiting the magnitude of parameter noise. This effect is not explicitly included in the ESS simulations in the main text, but it is conceptually covered by some of the experiments, where the network is restricted to only a small fraction of the wafer (Section 3.1.7), or where additionally parts of the synapses are declared as not available (Section 3.2.6).

From the measurements in Figure S1.1, we can e.g. estimate the variation of the voltages E^{spike}, E_{L}, $E^{\mathrm{rev,e}}$ and $E^{\mathrm{rev,i}}$ in the biological domain: For all, the vast majority of neurons has a trial-to-trial variation below 10 mV on the hardware, which corresponds to 1 mV in the biological when using a voltage scaling factor $\alpha_V = 10$ (cf. Equation S1.7).

Table S1.1. Parameter ranges of the BrainScaleS wafer-scale hardware

Description	Name	Min	Max	Unit	Comment
**Neuron (Adaptive Exponential Integrate&Fire)**					
Absolute refractory period	τ_{refrac}	0.16	10.0	ms	
Spike detection potential	E^{spike}	-125.0	45.0	mV	
Reset potential	E^{r}	-125.0	45.0	mV	
Leakage reversal potential	E_{L}	-125.0	45.0	mV	
Membrane time constant	τ_{m}	9	105	ms	
Adaptation coupling param	a	0	10.0	nS	adaptation can be fully disabled
Spike triggered adapt. param	b	0	86	pA	
Adaptation time constant	τ_w	20.0	780.0	ms	
Threshold slope factor	Δ_{T}	0.4	3.0	mV	exponential spike generation
Spike initiation threshold	E_{T}	-125.0	45.0	mV	can be fully disabled
Excitatory reversal potential	$E^{\mathrm{rev,e}}$	-125.0	45.0	mV	
Inhibitory reversal potential	$E^{\mathrm{rev,i}}$	-125.0	45.0	mV	
Exc. synaptic time constant	$\tau^{\mathrm{syn,e}}$	1.0	100.0	ms	
Inh. synaptic time constant	$\tau^{\mathrm{syn,i}}$	1.0	100.0	ms	
**Synapses**					
Weight	w^{syn}	0	0.300	µS	4-bit resolution
Axonal delay (on-wafer)	delay	1.2	2.2	ms	not configurable
**Short Term Plasticity**					
Utilization of synaptic efficacy	U	0.11	0.47		possible values: $[\frac{1}{9}, \frac{3}{11}, \frac{5}{13}, \frac{7}{15}]$
Recovery time constant	τ_{rec}	40.0	900.0	ms	One of the two time constants
Facilitation time constant	τ_{facil}	35.0	200.0	ms	has to be set to 0.0. Available range depends on U (maximum range given).
**Stimulus**					
External spike sources	ν	0.0	4000	Hz	cf. [46]

All ranges correspond to a membrane capacitance of $C_{\mathrm{m}} = 0.2\,\mathrm{nF}$ and a hardware speedup of 10^4 compared to real time. It is possible to choose an arbitrary value for C_{m}, but then the ranges of parameters a, b and of the synaptic weights are multiplied by $\frac{C_{\mathrm{m}}}{0.2\,\mathrm{nF}}$.

Table S1.2. List of typical usage scenarios of the wafer-scale hardware system

Nr of Neurons	Synapses/ Neuron	DenMems/ Neuron	Neurons/ HICANN
196 608	224	1	512
98 304	448	2	256
49 152	896	4	128
24 576	1792	8	64
12 288	3584	16	32
6144	7168	32	16
3072	14 336	64	8

One can either opt for many neurons with few synapses or for fewer neurons but a higher connection density.

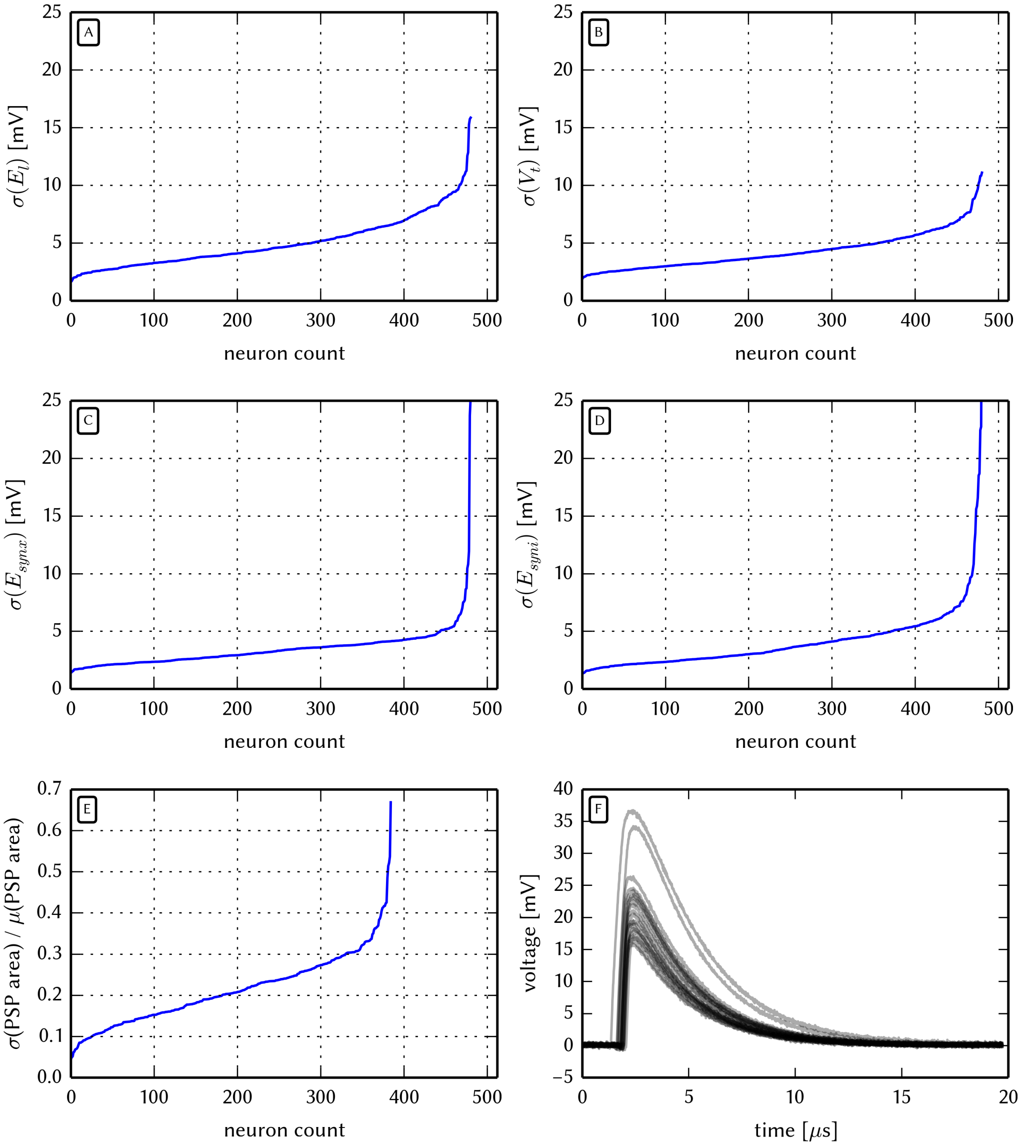

Figure S1.1. (A-D) Cumulative distribution of trial-to-trial variation for selected parameters. Each graph shows the number of neurons on one chip with a standard deviation of the measured value that is less than the value shown on the ordinate. All values are given in hardware units. In order to obtain values in the biological domain (Section 2.2), the voltages must be divided by the conversion factor of $\alpha_V = 10$ (cf. Equation S1.7). The standard deviation was estimated from 30 measurements for each neuron. (A) Leakage potential (B) Threshold potential (C, D) Excitatory and inhibitory reversal potential (E) Relative variation of the PSP integral. The standard deviation was estimated from 20 trials per neuron. Neurons were omitted from the measurements when an initial sweep over the available parameter range did not include the required PSP integral of 8×10^{-9} V s. (F) Example PSP traces for a randomly chosen neuron from the measurement in (E). In order to minimize readout noise, each trace is an average over 400 individual PSPs which were evoked in short succession without rewriting floating gate parameters. As the re-write variation is the main source of trial-to-trial variability (Section 2.1.1), the variation within the 400 samples is much smaller than the trial-to-trial variation that is shown in figures (E) and (F).
