# Supplementary material for: Characterization and Compensation of Network-Level Anomalies in Mixed-Signal Neuromorphic Modeling Platforms
Source: PLoS One. 2014 Oct 10;9(10):e108590. doi: 10.1371/journal.pone.0108590 (PMC4193761; doi:10.1371/journal.pone.0108590)
Supplement: Appendix S2 — Cortical layer 2/3 attractor memory. Methods, model parameters, and detailed simulation results of the Layer 2/3 model. (PDF) [file pone.0108590.s002.pdf]

Appendix S2 Cortical layer 2/3 attractor memory

S2.1 Original model parameters

In Table S2.1, Table S2.2, Table S2.3 and Table S2.4, we summarize the parameters and characteristics of the original model, as found in [59]. These have served as the basis for the model fit, for which the parameters can be found in the next subsection.

S2.2 Fitted Hardware-Compatible Parameters

Table S2.5, Table S2.6 and Table S2.7 contain all parameters required for the fits described in Section 3.1.3. All fits were performed by minimizing the L^2-norm of the distance between the simulated traces (Figure S2.1 **A - C, G - L**) or between spike timings (Figure S2.1 **D - F**).

The diffuse background stimulus was generated by Poisson spike sources at a total rate of 300 Hz per PYR cell.

Apart from random noise, the PYR cells further receive input from other PYR cells in cortical layer 4. The input intensity was calculated from the number of cells in layer 4 likely to project onto layer 2/3, which was estimated to be around 30 with a rate of approximately 10 Hz and a connection density of 25 % [59].

Therefore, a Poisson process with 75 Hz was used for each PYR cell input. Since we used static synapses for the Poisson input, the synaptic weights for source-PYR connections were chosen as 30% of PYR-PYR connections within the MCs. This was verified for compliance the original model from [59], which uses 7 to 8 sources per stimulated PYR cell with a rate of 10 Hz each and depressing synapses. For each stimulus event in the pattern completion and rivalry experiments (described below), layer 4 cells were set to fire for 60 ms. In each stimulated MC, 6 PYR cells were targeted from layer 4.

Table S2.8 shows the average firing rates for the different cell types in the network when only certain synapses are active.

S2.3 Delays

Each connection within the same MC was set to have constant synaptic delay of 0.5 ms. Additionally, axonal delays for connections between different MCs were realized by taking into account their spatial distance at an average axonal propagation speed of 200 µm/ms. Both the HCs in the whole network as well as the MCs within a single HC are laid out on a hexagonal grid with a edge length of 500 µm (HC↔HC) / 60 µm (MC↔MC). In the default network (9HC×9MC) this leads to delays between 0.5 ms and 8 ms.

S2.4 Scaling

Due to the modularity of this network model, several straightforward possibilities exist for increasing or decreasing its size without affecting its basic functionality. One can vary the total number of neurons simply by modifying the number of cells per MC. One can also vary the number of MCs per attractor by varying the total number of HCs. And finally, one can change the number of attractors by changing the number of MCs per HC accordingly.

All such changes need to be accompanied by corresponding modifications in connectivity in order to preserve the network dynamics. This has been done by keeping the average input current per neuron within an active attractor constant, which is equivalent to conserving the fan-in for every neuron from every one of its afferent populations and leads to the scaling rules shown in Table S2.9. In order to facilitate a comparison with the original results from [59] and [60], we have only considered homogeneous changes, meaning that all modules (MCs, HCs) were equal in size and symmetrically connected.

Table S2.1. Original neuron parameters

Parameter	PYR	RSNP	BAS	Unit
g_{ext}	0.082	0.15	0.15	$\mu S/mm^2$
E_{leak}	-75	0.15	-75	mV
E_{Na}	50	50	50	mV
E_{Ca}	150	150	150	mV
E_K	-80	-80	-80	mV
$E_{Ca(NMDA)}$	20	20	20	mV
g_L	0.74	0.44	0.44	$\mu F/mm^2$
C_m	0.01	0.01	0.01	$\mu F/mm^2$
Soma diameter $\pm$ stdev	21 $\pm$ 2.1	7 $\pm$ 0.7	7 $\pm$ 0.7	μm
g_{Na} initial segment	2500	2500	2500	$\mu S/mm^2$
g_K initial segment	83	5010	5010	$\mu S/mm^2$
g_{Na} soma	150	150	150	$\mu S/mm^2$
g_K soma	250	1000	1000	$\mu S/mm^2$
g_{NMDA}	75.0	75.0	-	$\mu S/mm^2$
Ca_V influx rate	1.00	1.00	1.00	$mV^{-1}ms^{-1}mm^{-2}$
Ca_{NMDA} influx rate	2.96	0.0106	-	$s^{-1}mV^{-1}\mu S^{-1}$
Ca_V decay rate	6.3	4	-	s^{-1}
Ca_{NMDA} decay rate	1	1	-	s^{-1}
g_K (Ca_V)	29.4	105	0.368	nS
g_K (Ca_{NMDA})	40	40	-	nS
# compartments	6	3	3	
Dendritic area (relative soma)	4	4	4	
Initial segment area (relative soma)	0.1	0.1	0.1	

Table S2.2. Original synapse parameters

Pre $\rightarrow$ Post	Type	Duration [s]	τ_{raise} [s]	τ_{decay} [s]	E^{rev} [mV]	U	τ_{rec} [s]	E_{slow} [mV]
PYR $\rightarrow$ PYR	Kainate/AMPA	0.0	0.0	0.006	0	0.25	0.575	-
PYR $\rightarrow$ PYR	NMDA	0.02	0.005	0.150	0	0.25	0.575	0.020
PYR $\rightarrow$ BAS	Kainate/AMPA	0.0	0.0	0.006	0	-	-	-
PYR $\rightarrow$ RSNP	Kainate/AMPA	0.0	0.0	0.006	0	-	-	-
PYR $\rightarrow$ RSNP	NMDA	0.02	0.005	0.150	0	-	-	0.020
BAS $\rightarrow$ PYR	GABA	0.0	0.0	0.006	-85	-	-	-
RSNP $\rightarrow$ PYR	GABA	0.0	0.0	0.006	-85	-	-	-

Table S2.3. Original network structure: number of neurons per functional unit

	HCs	MCs	PYR	BAS	RSNP	total neurons
per MC	-	-	30	1	2	33
per HC	-	8	240	8	16	264
network total	9	72	2160	72	144	2376

Table S2.4. Original network structure: connection probabilities

within an MC	
PYR → PYR	0.25
RSNP → PYR	0.70
between MCs inside the same HC	
PYR → BAS	0.70
BAS → PYR	0.70
between MCs in different HCs	
PYR → PYR	0.30
PYR → RSNP	0.17

Table S2.5. Fitted neuron parameters for the L2/3 model

Parameter	PYR	RSNP	BAS	Unit	Comment
C_{m}	0.179	0.0072	0.00688	nF	from the fits in Figure S2.1 **A-C**
$E^{\mathrm{rev,e}}$	0.0	0.0	0.0	mV	difference to original model compensated by synaptic weights
$E^{\mathrm{rev,i}}$	-80.0	-	-	mV	difference to original model compensated by synaptic weights
τ_{m}	16.89	15.32	15.64	ms	from the fits in Figure S2.1 **A-C**
τ_{refrac}	0.16	0.16	0.16	ms	minimum available in hardware at the used speedup
$\tau^{\mathrm{syn,e}}$	17.5	66.6	6.0	ms	see paragraph "Synapses"
$\tau^{\mathrm{syn,i}}$	6.0	-	-	ms	see paragraph "Synapses"
V_{reset}	-60.7	-72.5	-72.5	mV	from the fits in Figure S2.1 **D-F**
E_{L}	-61.71	-57.52	-56.0	mV	from the fits in Figure S2.1 **D-F**
a	0.0	0.28	0.0	nS	see fig from the fit in Figure S2.1 **B**
b	0.0132	0.00103	0.0	nA	from the fits in Figure S2.1 **D**, **E**
ΔT	0.0	0.0	0.0	mV	from the fits in Figure S2.1 **D-F**
τ_w	196.0	250.0	0.0	ms	from the fits in Figure S2.1 **D**, **E**
E^{spike}	-53.0	-51.0	-52.5	mV	from the fits in Figure S2.1 **D-F**
V_T	-	-	-	mV	not used since $\Delta T = 0$

Table S2.6. Fitted synapse parameters for the L2/3 model

Pre-Post	type	weight [μS]	τ^{syn} [ms]	U	τ_{rec} [ms]	τ_{facil} [ms]
PYR-PYR (local)	exc	0.004125	17.5	0.27	575.	0.
PYR-PYR (global)	exc	0.000615	17.5	0.27	575.	0.
PYR-BAS	exc	0.000092	6.0	-	-	-
PYR-RSNP	exc	0.000024	66.6	-	-	-
BAS-PYR	inh	0.0061	6.0	-	-	-
RSNP-PYR	inh	0.0032	6.0	-	-	-
background-PYR	exc	0.000224	17.5	-	-	-

Table S2.7. Stimulus parameters for the L2/3 model

**Background**	
# of sources per PYR	1
rate	300 Hz
weight	0.000 224 µS
**Shared background pool**	
# of sources per PYR	100 out of 5000 total
rate	3 Hz
weight	0.000 224 µS
**L4**	
# of sources per MC	5
$p_{\mathrm{L4}\rightarrow\mathrm{PYR}}$	0.75
weight	0.001 237 5 µS<br>(30% local PYR→PYR)

Table S2.8. Average firing rates (in Hz) of the different cell types of the L2/3 model with only certain synapses active

setup no.	active synapses	ν_{PYR}	ν_{RSNP}	ν_{BAS}
1	background-PYR, PYR-BAS, PYR-RSNP	0.738 ± 0.096	57.946 ± 6.993	4.655 ± 1.081
2	same as 1 + BAS-PYR	0.174 ± 0.021	13.430 ± 1.910	1.119 ± 0.441
3	same as 1 + RSNP-PYR	0.257 ± 0.037	20.375 ± 2.536	1.783 ± 0.954
4	same as 2 + 3 + PYR-PYR (local)	0.200 ± 0.030	14.679 ± 2.261	1.258 ± 0.544
5	same as 2 + 3 + PYR-PYR (global)	0.204 ± 0.078	14.954 ± 5.680	1.337 ± 0.625

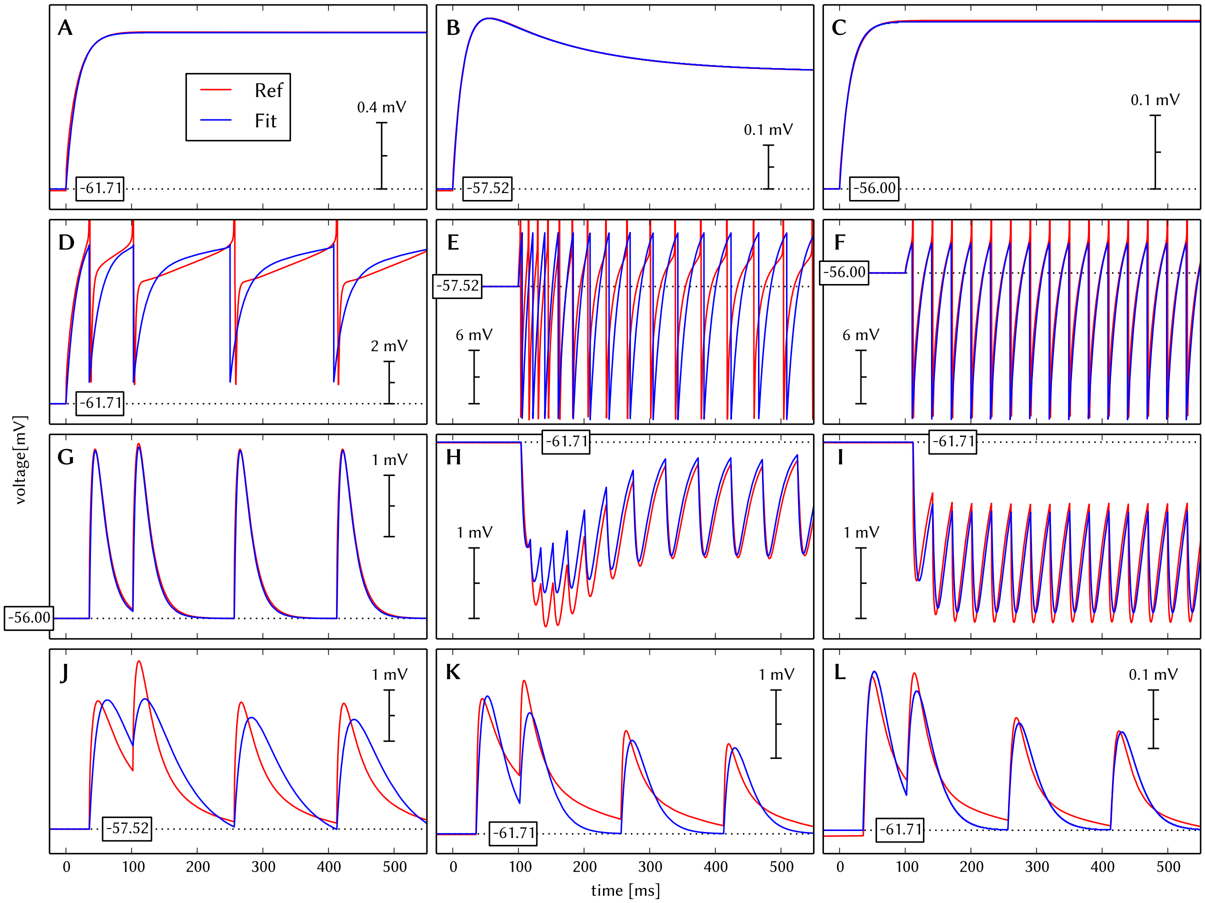

Figure S2.1. Comparison of original neuron and synapse dynamics to the fitted dynamics of hardware-compatible models. (**A - C**) Membrane potential of the three different cell types (PYR, RSNP, and BAS, respectively) under subthreshold current stimulation. These were used to determine the rest voltage E_{L}, total equivalent membrane capacitance C_{m}, membrane time constant τ_{m} and the adaptation coupling parameter a. (**D - F**) Membrane potential of the three different cell types (PYR, RSNP, and BAS, respectively) under spike-inducing current stimulation. While the precise membrane potential time course of the original neuron model can not be reproduced by a single-compartment AdEx neuron, it was possible to reproduce the spike timing and especially average firing rates quite accurately. A small deviation of spiking frequency can be observed for RSNP cells during the first 50 ms - in the original model, they adapt slower than their AdEx counterparts. From these fits, the values for the absolute refractory period τ_{refrac}, reset voltage V_{reset}, threshold voltage V_T, slope factor ΔT, spike-triggered adaptation b and adaptation time constant τ_w were extracted. (**G - L**) PSP fit results for all synapse types of the L2/3 model (PYR→BAS, RSNP→PYR, BAS→PYR, PYR→RSNP, PYR→PYR within a MC, and PYR→PYR between MCs, respectively). The output spikes from **D - F** have been used as input. These fits were used to determine synaptic weights w^{syn}, time constants τ^{syn} and the TSO parameters U and τ_{refrac}. Because the hardware synapses only support a single conductance decay time constant, as opposed to the two different time constants in the original model for AMPA/kainate and NMDA, we have chosen an intermediate value for τ^{syn}, which constitutes the main reason for the difference in PSP shapes. A second reason lies in the saturating nature of synaptic conductances in the original model, which can not be emulated on the hardware without affecting the required TSO parameters (see section 3.1.3).

Table S2.9. Scaling rules for the connection densities of the L2/3 model

Connection	Scaled conn. prob. $\tilde{p}$
PYR → PYR (same MC)	$29/(N_{\mathrm{PYR}} - 1) \cdot p$
PYR → PYR (different MC)	$30/N_{\mathrm{PYR}} \cdot 8/(N_{\mathrm{HC}} - 1) \cdot p$
PYR → RSNP	$30/N_{\mathrm{PYR}} \cdot 8/(N_{\mathrm{HC}} - 1) \cdot p$
PYR → BAS	$30/N_{\mathrm{PYR}} \cdot p$
RSNP → PYR	$2/N_{\mathrm{RSNP}} \cdot p$
BAS → PYR (Enlarging)	$1/N_{\mathrm{BAS}} \cdot p$
BAS → PYR (Shrinking)	$1/N_{\mathrm{BAS}} \cdot 8/N_{\mathrm{MC}} \cdot p$

N_x represents the number of units of type x (the original values are found in Table S2.3). p represents the original connection probability as found in table Table S2.4. Whenever a scaled probability $\tilde{p}$ exceeded 1, it was clipped to 1, but the weights of the corresponding synapses were also increased by $\tilde{w^{\mathrm{syn}}} = w^{\mathrm{syn}} \cdot \tilde{p}$.

The connections to the BAS cells required special treatment for two reasons. Firstly, during an active state, they receive input from a single MC, but are excited by all MCs in a HC during the competition period between active attractors. Only one aspect can be preserved when scaling and we have considered the dynamics during UP states as most important, leading to a "PYR → BAS" scaling rule independent of N_{MC}. Secondly, because PYR cells in MCs only project to the nearest 8 BAS cells, there are always precisely 8 active BAS cells per HC within an active attractor, which yields a simple "BAS → PYR" scaling rule. When decreasing the number of attractors however, the number of existing BAS cells per HC also decreases, making an appropriate connection density scaling necessary. This is the reason for the two different "BAS → PYR" scaling rules found in Table S2.9.

Table S2.10 shows the combinations of N_{HC} and N_{MC} used for the quantification of synapse loss after mapping the L2/3 model onto the hardware in Figure 11. In these mapping sweeps the diffusive background noise was modeled, as for the large-scale network ported to the ESS (Section 3.1.7), with a background pool of 5000 Poisson sources and every PYR cell receiving input from 100 of the sources.

S2.5 UP-state detection

One crucial element of the analysis is the detection of UP-states from which various other properties such as dwell times, competition times as well as average spike-rates in UP- and DOWN-states are determined. The method of choice for detecting UP-states is based on the fact that the mean spike rate of an attractor during an UP-state is much higher than the spike rate in all remaining patterns in their corresponding DOWN-states, whereas – in times of competition – two or more attractors have elevated but rather similar spike rates. A measure which quantifies this relationship is the standard deviation σ of all mean spike rates per attractor *at a given time* t. The attractor with index i is then said to be in an UP-state at time t if the following relation holds:

$$r_i(t) > c \cdot \sigma(t) > \max_{r \in \{1,\ldots,N_{\mathrm{MC}}\} n i} r_k(t) \quad , \tag{S2.1}$$

where $r_i(t)$ is the rate of attractor i at time t and c is a numerical constant which is set to 1.

This method of detection has several advantages: it is based exclusively on spike trains (and not voltages or conductances, which are more difficult to read out and require much more storage space), it has a clear notion of there being at most one UP-state at any given time and it is completely local (in

Table S2.10. Scaling table for the L2/3 model used for the synapse loss estimation in Figure 11

N_{HC}	N_{MC}	total neurons
18	2	1188
9	6	1782
27	3	2673
18	6	3564
36	4	4752
9	18	5346
18	12	7128
27	9	8019
18	18	10 692
18	36	21 384
36	24	28 512
36	36	42 768
27	54	48 114
45	45	66 825

time), meaning that a very large value somewhere on the time axis cannot bias the detection at other times.

In small networks with randomly spiking neurons, it might happen by chance that all but one of the spike rates lie below the (approximately) constant standard deviation. These falsely detected UP-states are very short and can thus easily be filtered out by requiring a minimal duration for UP states, which we chose at $100\,ms$. This value was chosen after investigating dwell time histograms, as it distinguishes reliably between random fluctuations and actual active attractors.

S2.6 Pattern Completion

Pattern completion is a basic property of associative-memory networks. By only stimulating a subset of PYR cells pwithin a pattern, the complete pattern is recalled. The activity first spreads within the stimulated MCs, turning them dominant in their corresponding HCs. After that, the activity spreads further to other HCs – while the already dominating MCs stabilize each other through mutual stimulation – activating the whole pattern while suppressing all others. All PYR cells in the corresponding attractor hence enter an UP-state.

To verify the pattern completion ability of the network, a series of simulations was performed. In order to reduce the occurrence of spontaneously activating attractors – which would interfere with the activation of the stimulated attractor – competition was investigated in larger networks of size 25HC×25MC, as they exhibit almost no spontaneous attractors (the competition time fractions are much higher, see Figure 6 **H**).

For each network, all of the 25 patterns were stimulated one by one in random order. The time between consecutive stimuli was chosen to be 1000 ms to ensure minimal influence between patterns. The number of stimulated MCs (one per HC) was varied over the course of multiple simulations.

After simulation, each network was analyzed for successfully activated patterns. An activation attempt was said to be successful if the stimulated pattern was measured as active within 200 ms after the stimulus onset. If another pattern was active up to 75 ms or if the stimulated pattern had already been active between $20 - 500$ ms prior to the stimulus onset, the attempt was deemed invalid and ignored during the calculation of success ratios. This was done to take into account the fact that a pattern is more difficult

to activate when another one is already active or while it is still recovering from a prior activation. From all valid attempts the success probability (assuming a binomial distribution of successful trials) was estimated using the Wilson interval

$$\tilde{p} = \frac{1}{1 + \frac{z^2}{n}} \left[\hat{p} + \frac{z^2}{2n} \pm z \sqrt{\frac{\hat{p}(1-\hat{p})}{n} + \frac{z^2}{4n^2}} \right] \tag{S2.2}$$

where $\hat{p}$ represents the success ratio, n the number of valid attempts and $z = 1$ the desired quantile.

For most experiments (regular, synaptic weight noise and homogeneous synaptic loss) the number of invalid activations was always below 5 (out of 25). The only exception was the PYR population size scaling: starting at 15 PYR cells, the validity rate roughly halves for every reduction in size (by 5 PYR cells per step) due to the increased occurrence of spontaneous attractors. For simulations carried out on the ESS, only 10 patterns out of 25 were stimulated. Out of these 10 attempts, only 5 were valid, on average.

S2.7 Pattern Rivalry / Attentional Blink

Another important feature of the L2/3 model is its ability to reproduce the attentional blink phenomenon, i.e., the inability of one pattern, stimulated by layer 4 input, to terminate another already active pattern and become active itself. This phenomenon was investigated through a series of different networks of same size as in Section S2.6 (25HC×25MC). For each network, 24 out of 25 patterns were randomly assigned to 12 pairs. Afterwards, pattern rivalry was tested on all of these pairs in intervals of 1000 ms.

Let the two patterns in each pair be denoted A and B. In order to guarantee an immediate activation of pattern A, 6 out of 25 HCs were stimulated (as then all completion attempts are successful, see Figure 6 **N**). Then, after a certain delay ΔT, pattern B was stimulated with a varying amount of HCs. Both the number of stimulated HCs as well as the delay ΔT were varied for each network.

The same way as in Section S2.6, each network was then analyzed as to whether pattern B was successfully activated or not. If the competing pattern B was activated within 200 ms after the stimulus onset and stayed active for at least 100 ms, the attempt was counted as successful, otherwise it was deemed unsuccessful. As before, attempts during which spontaneously activated patterns intervened were ignored. From all successful and unsuccessful attempts, the success probability was then estimated the same way as in pattern completion, using Equation S2.2.

The validity ratios for pattern rivalry are not significantly different from those discussed in Section S2.6. Most experiments (regular, synaptic weight noise and homogeneous synaptic loss) have 10 to 12 valid attempts (out of 12). As before, for the PYR population size scaling experiments, the number of valid attempts dropped progressively (8.2 ± 1.7, 4.8 ± 2.1 and 2.2 ± 1.5 valid attempts for 15, 10 and 5 PYR per MCs respectively). Simulations carried out on the ESS had an average of 4 (distorted case) and 6 (compensated case) valid attempts (out of 10).

Different network configurations have been compared in terms of *attentional blink* by estimating the 0.5 iso-probability contour in the following way. For every delay ΔT, the transition point from below to above 0.5 probability for successful activation of the second pattern was estimated by linearly interpolating between the two nearest data points with a success ratio of above and below 0.5, respectively. In case there were several such transition points only the one with the highest stimulus was considered. If no transition point could be identified, the transition was fixed at at either 25 or 0 stimulated MCs, depending on whether all success ratios were above or below 0.5. When there were no valid attempts for a certain delay/stimulus pair, its success probability estimate was replaced by the median of all valid activation attempts for that particular time delay ΔT (this only occurred sporadically in ESS and PYR population size scaling with less than 15 PYR cells per MC). After identifying the transition point for every time delay ΔT, intermediate values were interpolated linearly. Finally, the interpolated values were Gauss-filtered ($\mu = 0.25 \times$ step size for ΔT in the dataset) to better approximate the true 0.5 iso-probability

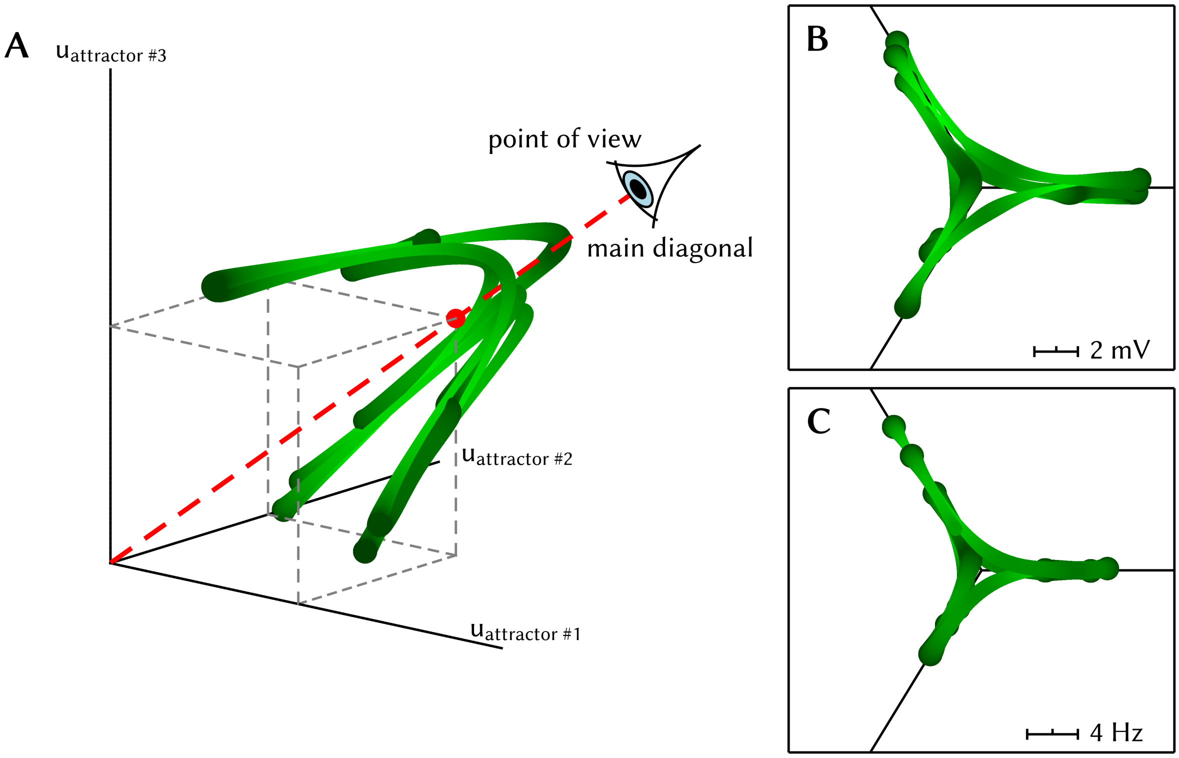

Figure S2.2. Visualization of the star plot as a projection in the case of a three-dimensional state space. (**A**) Illustration of the view point with average membrane voltage data plotted in three-dimensional Cartesian coordinates. The data was taken from a (9HC×3MC)-network and covers a 2.5 s period of network activity. (**B**) Resulting star plot from regular view point. (**C**) Star plot of the corresponding average attractor rate data.

contour.

S2.8 Star plots

While the spiking activity of many cells can be visualized quite well in raster plots, illustrating the temporal evolution of their membrane potentials is less straightforward. Here, we have chosen to use so-called star plots for visualizing both average voltages and average firing rates of entire cell populations.

In a system evolving in an abstract space with 3 dimensions, a star plot represents the orthogonal projection of the state space trajectory along the main diagonal of the corresponding Cartesian coordinate system onto a plane perpendicular to it. For n dimensions, points $\mathbf{x}$ in the star plot are no longer projections of the states $\mathbf{z}$, but are rather calculated as

$$\mathbf{x} = \sum_{i=1}^{n} z_i \left(\cos \frac{2\pi i}{n}, \sin \frac{2\pi i}{n} \right) \tag{S2.3}$$

A visualization for $n = 3$ is illustrated in Figure S2.2.

In case of the L2/3 network, the number of dimensions is given by the number of attractors, with each axis describing some particular feature of the corresponding attractor (such as the average voltage

or spike rate of the constituent PYR cells).

In addition to the position in state space, the state space velocity is also encoded in a star plot by both the thickness and the color of the trajectory. Especially in the case of the L2/3 network, this can be very useful in visualizing e.g. attractor stability or competition times. Here, both line thickness and lightness were chosen proportional to (const $+ e^{-|d\mathbf{x}|/dt}$), with $\mathbf{x}$ being the position in state space.

Figure S2.3 **B** and **C** show two characteristic examples of star plots used for visualizing the dynamics of the L2/3 network.

S2.9 Average synaptic conductance due to Poisson stimulation

For a single Poisson source with rate ν_i connected to the neuron by a synapse with weight w_i and time constant τ^{syn}, the conductance course can be viewed as a sum of independent random variables, each of them representing the conductance change caused by a single spike. In the limit of large ν_i, the central limit theorem guarantees the convergence of the conductance distribution to a Gaussian, with moments given by

$$\begin{aligned}
\langle g_i^{\text{syn}} \rangle &= \sum_{\text{spk}\, s} \langle w_i \Theta(t - t_s) \exp\left(-\frac{t - t_s}{\tau^{\text{syn}}}\right) \rangle \\
&= \lim_{T\to\infty} \frac{\langle N \rangle}{T} w_i \int_0^T \exp\left(-\frac{t}{\tau^{\text{syn}}}\right) \, dt \\
&= w_i \nu_i \tau^{\text{syn}} \quad . \qquad \text{(S2.4)} \\
\text{Var}\left[g_i^{\text{syn}}\right] &= \sum_{\text{spk}\, s} \text{Var}\left[w_i \Theta(t - t_s) \exp\left(-\frac{t - t_s}{\tau^{\text{syn}}}\right)\right] \\
&= \lim_{T\to\infty} \langle N \rangle \left\{ \langle \left[w_i \Theta(t) \exp\left(-\frac{t}{\tau^{\text{syn}}}\right)\right]^2 \rangle \right. \\
&+ \left. \langle \left[w_i \Theta(t) \exp\left(-\frac{t}{\tau^{\text{syn}}}\right)\right] \rangle^2 \right\} \\
&= \lim_{T\to\infty} \nu_i T \left\{ \frac{1}{T} w_i^2 \int_0^T \exp\left(-2\frac{t}{\tau^{\text{syn}}}\right) \, dt \right. \\
&- \left. \frac{1}{T^2} \left[\int_0^T \exp\left(-\frac{t}{\tau^{\text{syn}}}\right) \, dt\right] \right\} \\
&= \frac{w_i^2 \nu_i \tau^{\text{syn}}}{2} \quad . \qquad \text{(S2.5)}
\end{aligned}$$

Since conductances sum up linearly, N Poisson sources lead to an average conductance of

$$\begin{aligned}
\langle g^{\text{syn}} \rangle &= \langle \sum_{i=1}^{N} g_i^{\text{syn}} \rangle \\
&= N \langle w \rangle \langle \nu \rangle \tau^{\text{syn}} \quad . \qquad \text{(S2.6)}
\end{aligned}$$

S2.10 Detailed simulations of synapse loss and PYR population reduction

Figure S2.3 and S2.4 show the effects of various levels of synapse loss and PYR population reduction, respectively.

S2.11 Synaptic weight noise

As can be seen in Figure S2.5, the firing rate of single PYR cells is highly dependent on the synaptic input weight that connects them to their respective Poisson source. For example, a variation of 20% in the input weight can cause the firing rate to either effectively vanish or more than triple. This heavily distorts network dynamics as PYR cells within MCs will exhibit highly disparate firing rates, thereby disrupting the network's ability to maintain stable UP states (in which all participating PYR cells should fire roughly with the same rate).

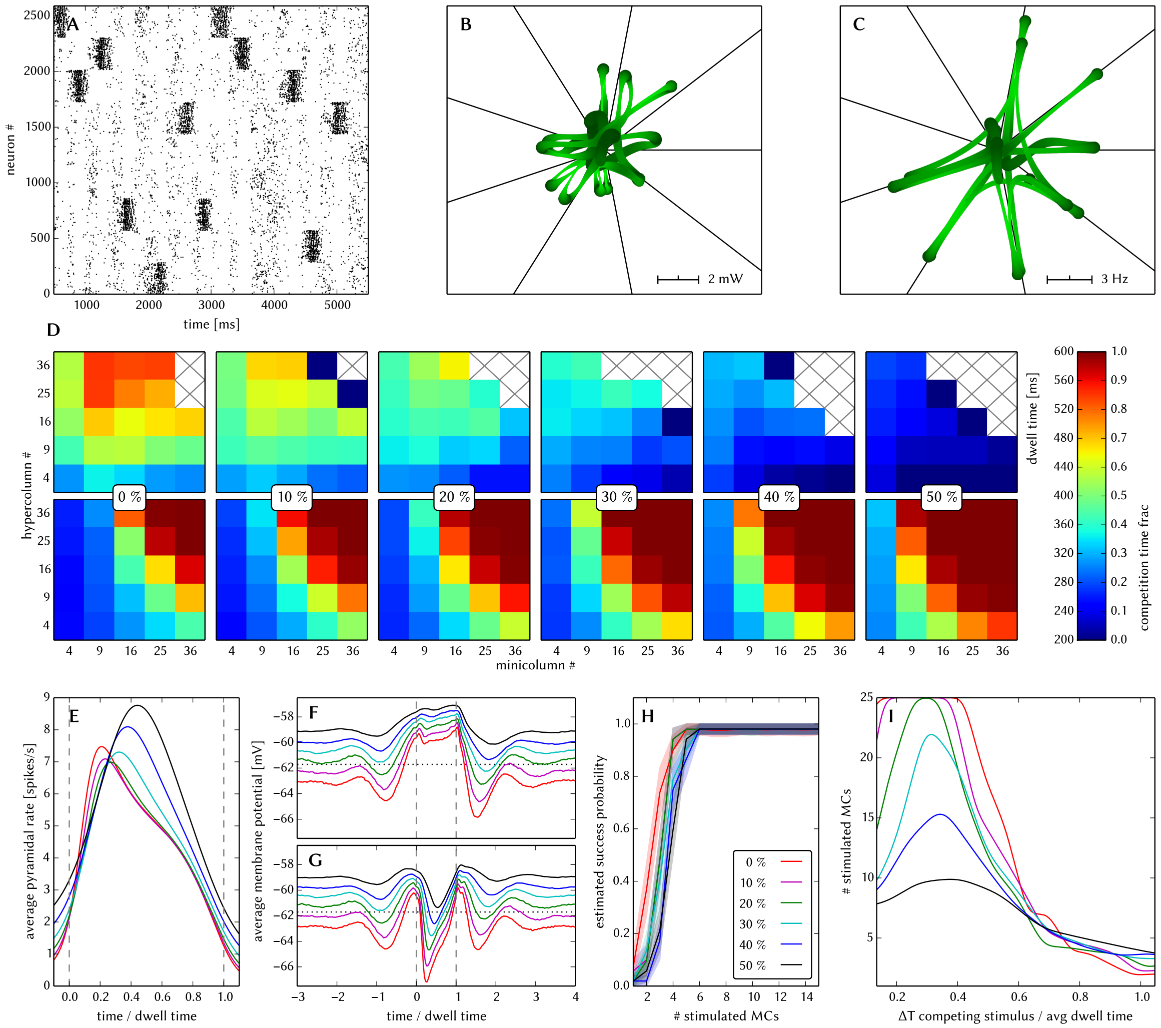

Figure S2.3. Effects of homogeneous synapse loss on the L2/3 model. Unless explicitly stated otherwise, the default network model (9HC×9MC) was used. The topmost 3 figures exemplify the dynamics of the network at 50% synapse loss, all other figures show the effects of various degrees of synapse loss (0-50%). (**A**) Raster plot of spiking activity. Only PYR cells are shown. The MCs are ordered such that those belonging to the same attractor (and *not* those within the same HC) are grouped together. (**B**) Star plot of average PYR cell voltages from a sample of 5 PYR cells per MC. (**C**) Star plot of average PYR cell firing rates. (**D**) Average dwell times and relative competition times for various network sizes. (**E**) Average firing rate of PYR cells during an UP state. (**F**) Average voltage of PYR cells before, during and after their parent attractor is active (UP state). (**G**) Average voltage of PYR cells before, during and after an attractor they do not belong to is active. For the previous three plots, the abscissa has been subdivided into multiples of the attractor dwell time. In subplots **F** and **G** the dotted line indicates the leak potential E_L of the PYR cells. (**H**) Pattern completion in a 25HC×25MC network. (**I**) Attentional blink in a 25HC×25MC network: $p = 0.5$ iso-probability contours.

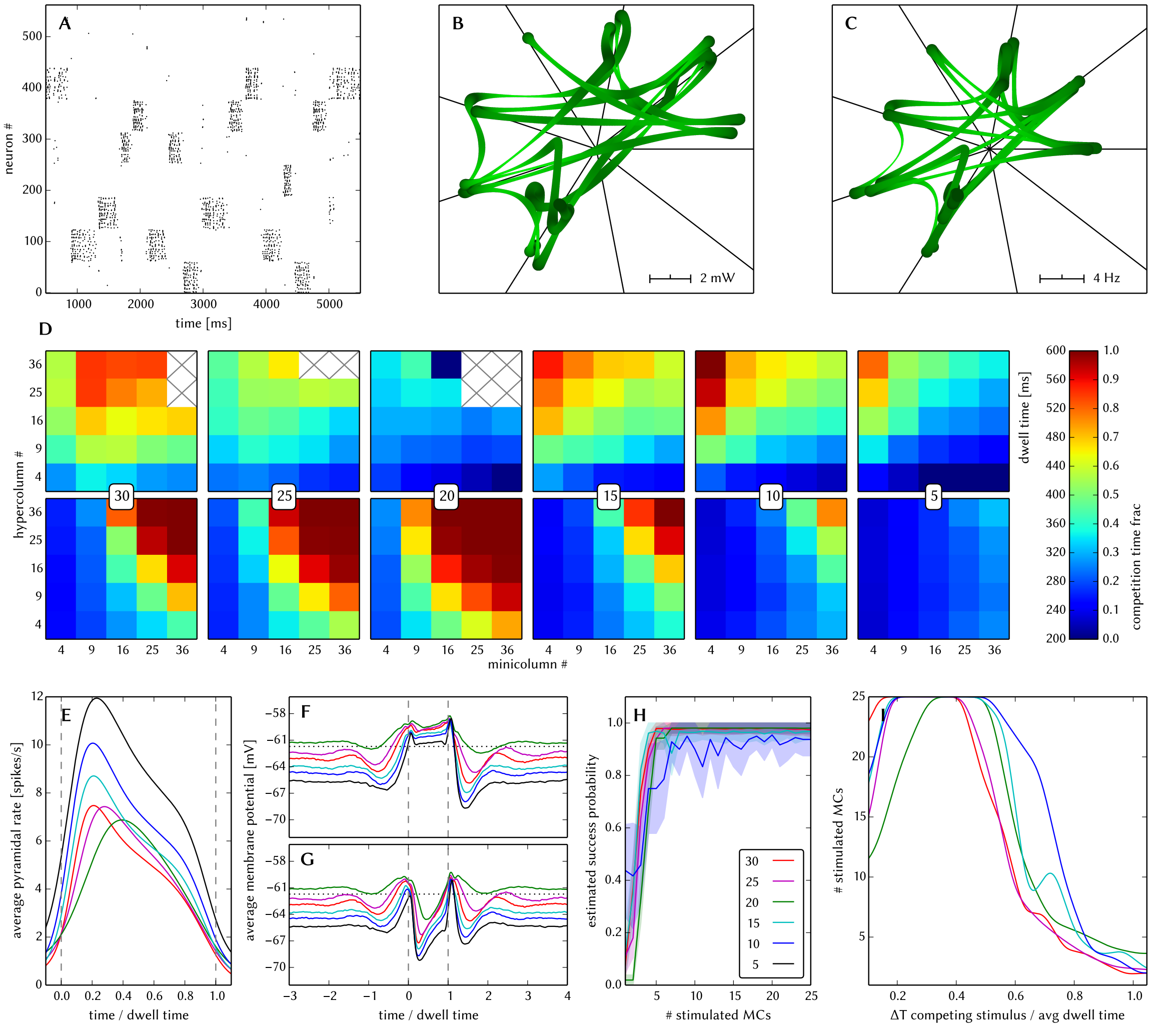

Figure S2.4. Effects of PYR population size scaling on the L2/3 model. Unless explicitly stated otherwise, the default network model (9HC×9MC) was used. The topmost 3 figures exemplify the dynamics of the network at 50% of its original PYR population size, all other figures show the effects of various degrees of PYR population reduction (0-50%). (**A**) Raster plot of spiking activity. Only PYR cells are shown. The MCs are ordered such that those belonging to the same attractor (and *not* those within the same HC) are grouped together. (**B**) Star plot of average PYR cell voltages from a sample of 5 PYR cells per MC. (**C**) Star plot of average PYR cell firing rates. (**D**) Average dwell times and relative competition times for various network sizes. (**E**) Average firing rate of PYR cells during an UP state. (**F**) Average voltage of PYR cells before, during and after their parent attractor is active (UP state). (**G**) Average voltage of PYR cells before, during and after an attractor they do not belong to is active. For the previous three plots, the abscissa has been subdivided into multiples of the attractor dwell time. In subplots **F** and **G** the dotted line indicates the leak potential $E_{\rm L}$ of the PYR cells. (**H**) Pattern completion in a 25HC×25MC network. (**I**) Attentional blink in a 25HC×25MC network: $p = 0.5$ iso-probability contours. In **H** and **I**, the dataset for 5 PYR cells per MC was omitted because of its extremely low validity rate.

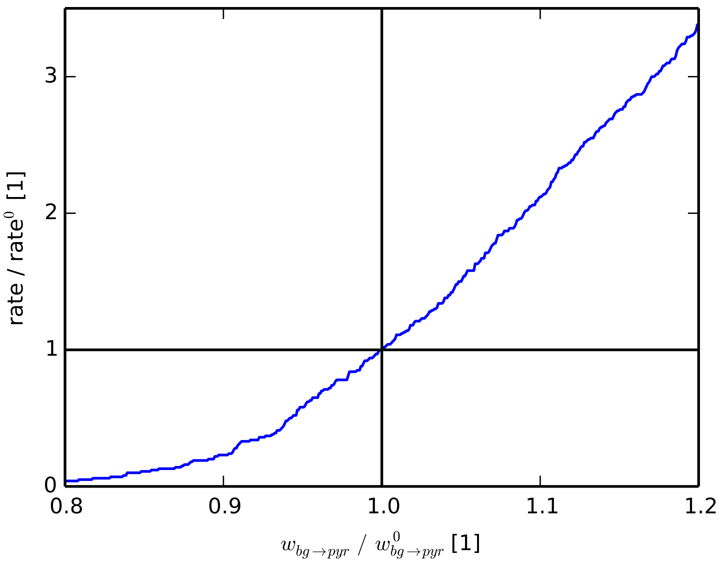

Figure S2.5. Single PYR cell firing rate for different synaptic input weights. Each weight configuration was simulated for 100 s.
