# Supplementary material for: Characterization and Compensation of Network-Level Anomalies in Mixed-Signal Neuromorphic Modeling Platforms
Source: PLoS One. 2014 Oct 10;9(10):e108590. doi: 10.1371/journal.pone.0108590 (PMC4193761; doi:10.1371/journal.pone.0108590)
Supplement: Appendix S3 — Synfire chain with feed-forward inhibition. Model parameters and additional simulations of the synfire chain network. (PDF) [file pone.0108590.s003.pdf]

Appendix S3 Synfire chain with feed-forward inhibition

S3.1 Model parameters

The neuron and connectivity parameters are given in Table S3.1 and Table S3.2.

S3.2 Network scaling

In the default setup studied in this article, the synfire chain consists of 6 groups of 125 neurons (100 excitatory and 25 inhibitory). In order to quantify the amount of synapse loss after mapping the network to the BrainScaleS wafer-scale hardware for different network sizes, we define the following network scaling rules. When increasing the network size, we vary both the number of synfire groups and the number of neurons per group while keeping the number of incoming synapses per neuron constant (cf. Table S3.2). The fraction of inhibitory neurons always amounts to 20 %. Neuron and synapse parameters are not altered. Table S3.3 lists the combinations of group size and group count used for the synapse loss estimation in Figure 17 **A**.

The background Poisson stimulus is scaled as follows. For the hardware implementation of the synfire chain we can not use one individual Poisson source for each neuron due to input bandwidth limitations. Instead, we assume one pool of 32 Poisson sources for each synfire group, and each neuron receives input from 8 random sources from that pool. The size of the background pool is then scaled with the number of neurons per synfire group, while always drawing 8 sources from the pool per neuron. This scaling of the background pool was chosen to make the total number of background sources proportional to the total number of neurons and independent of the group count.

S3.3 Additional simulation

S3.3.1 All distortion mechanisms

To check that the compensation methods do not interfere with each other, all distortion mechanisms were applied simultaneously with weight noise values of 20 % and 50 % and synapse loss values of 30 % and 50 %, with an axonal delay of 1.0 ms. Without compensation no stable region exists in all four cases. Figure S3.2 shows the result with all compensation methods applied. When several methods required modification of a network parameter, all modifications were applied. For instance, in the case of the synaptic weight which was scaled by both synapse loss and delay compensation methods, both scaling factors were multiplied. Figure S3.2 shows the restoration of input selectivity in all four cases.

Table S3.1. Neuron parameters used in the synfire chain benchmark model

Parameter	Value	Unit
C_{m}	0.29	nF
τ_{refrac}	2	ms
E^{spike}	-57	mV
E^{r}	-70	mV
E_{L}	-70	mV
τ_{m}	10	ms
$E^{\mathrm{rev,e}}$	0	mV
$E^{\mathrm{rev,i}}$	-75	mV
$\tau^{\mathrm{syn,e}}$	1.5	ms
$\tau^{\mathrm{syn,i}}$	10	ms

Table S3.2. Projection properties for the feed-forward synfire chain

Projection	weight µS	incoming synapses	delay ms
$RS_n \rightarrow RS_{n+1}$	0.001	60	20
$RS_n \rightarrow FS_{n+1}$	0.0035	60	20
$FS_n \rightarrow RS_n$	0.002	25	4

Table S3.3. Scaling table for the synfire chain used for the synapse loss estimation in Figure 17 A

groups	group size	total neurons
8	125	1000
16	125	2000
24	125	3000
20	200	4000
25	200	5000
15	400	6000
20	350	7000
20	400	8000
30	300	9000
25	400	10 000
20	500	10 000
40	500	20 000
60	500	30 000
40	1000	40 000
50	1000	50 000
30	2000	60 000
20	3500	70 000
20	4000	80 000
30	3000	90 000
25	4000	100 000

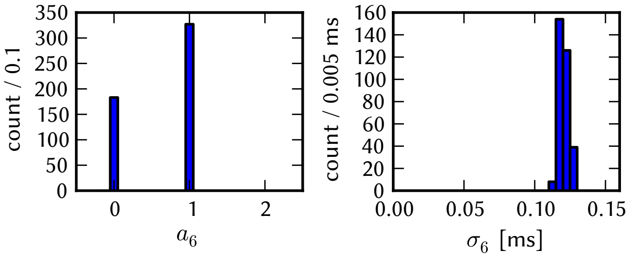

Figure S3.1. Distribution of a_6 and σ_6 in the reference experiment for the synfire chain model.

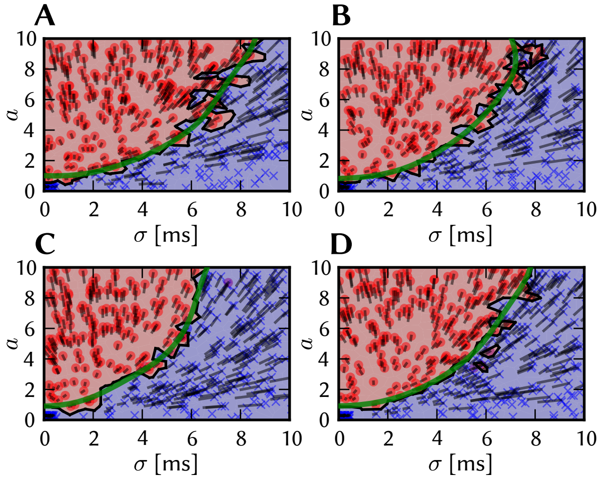

Figure S3.2. (σ, a) state space of the synfire chain model with all compensation methods applied for four different levels of distortion. **(A)** 30 % synapse loss, 20 % weight noise **(B)** 30 % synapse loss, 50 % weight noise **(C)** 50 % synapse loss, 20 % weight noise **(D)** 50 % synapse loss, 50 % weight noise

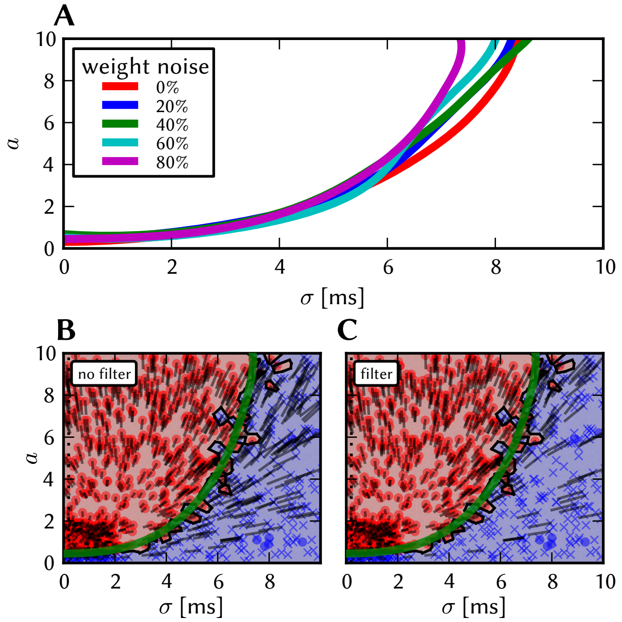

Figure S3.3. Demonstration of spontaneous event filter in the weight noise compensation (Section S3.3.4). **(A)** The same experiment as in Figure 15 **C** (weight noise with active compensation) but without the filter for background spikes. The separatrix locations are comparable as the filter does not influence the result significantly in the compensated case. (**B**, **C**) Complete state space response for weight noise of 80%, once with, once without filter. This demonstrates that the applied filter does not affect the result in the compensated case.

S3.3.2 Separatrix fit

To compare different separatrices, the a-values of the last group are characterized as successful $(+1)$ or extinguished (-1) and the resulting values interpolated and smoothed by a gaussian kernel with a standard deviation $(1.5\,\mathrm{ms}, 1.5)$ in the (σ, a) space. The iso-contour line of the resulting surface at a value of 0 is used as an approximation of the separatrix location, as shown in Figure 13 **C** together with the individual simulation results. Data points with $\sigma \leq 0.2\,\mathrm{ms}$ were not included in the fit to avoid distortions induced by bandwidth limitations in ESS simulations (Section 3.2.6) from affecting the fit quality. The data points are still shown individually as blue dots and regions, e.g., in Figure 17. This modification was also included in the software simulations for consistency. Cases in which the separatrix does not capture the relevant behavior, e.g., if the separation is not reliable in a large region of the state space, are shown separately.

S3.3.3 Weight noise compensation

Figure S3.3 **A** shows the separatrix in the case of compensated weight noise.

S3.3.4 Filtering of spontaneous activity

To prevent spontaneous background events from impeding the analysis, spikes are discarded as part of spontaneous activity if less then N spikes in the same excitatory group occur in a time window of $\pm T$. The utilized values for N and T are given at each point where the filter is applied; They are chosen such that authentic synchronous volleys with $a \geq 0.5$ (which would be counted as successful propagation, as defined above) are not removed. Figure S3.3 **B** and **C** show that the influence of the filter for spontaneous activity is minimal in the compensated case.

S3.3.5 Further ESS simulations

Distortion and compensation without synapse loss For the ESS simulation in section 3.2.6 we enforced a certain amount of synapse loss by restricting the synfire chain network to very limited hardware resources. However, due to its feed-forward structure, the network can be easily mapped onto the BrainScaleS hardware without any synapse loss (Figure 17 **A**). Thus, we also investigated the network without synapse loss, such that the active distortion mechanisms in the ESS simulations were synaptic weight noise, non-configurable axonal delays as well as spike loss and jitter. The state space of the distorted network (Figure S3.4 **A**) contains only a small and loosely connected region of sustained activity which indicates unreliable separation. Applying the compensation mechanism for synaptic weight noise and axonal delays fully restores the filter property of the synfire chain, as can be seen in Figure S3.4 **B**, where different separatrices mimic different delay-dependent realizations. Compared to the compensation for all distortion mechanisms, the compensated state space without synapse loss does not show any flaws (**C**).

Effect of spike loss and jitter We investigated the effect of spike loss and jitter in the HICANN, where the spikes of the neurons connected to the same on-wafer routing bus are processed subsequently (Section 2.1.2), which can lead to spike time jitter and in rare cases to spike loss when firing is highly synchronized.

Which 64 neurons inject their spikes into a routing bus is determined by the placement of the neurons on the HICANN. Hence, in order to study the effect of spike loss and jitter, we simulated the synfire chain network in two different placement setups: First, neurons of the same synfire group were placed sequentially onto the same routing bus, and second, neurons were distributed in a round-robin manner over different routing buses, such that neurons of different groups injected their spikes into one routing bus. Hence, we expect the spiking activity on each routing bus to be more synchronous in the first case than in

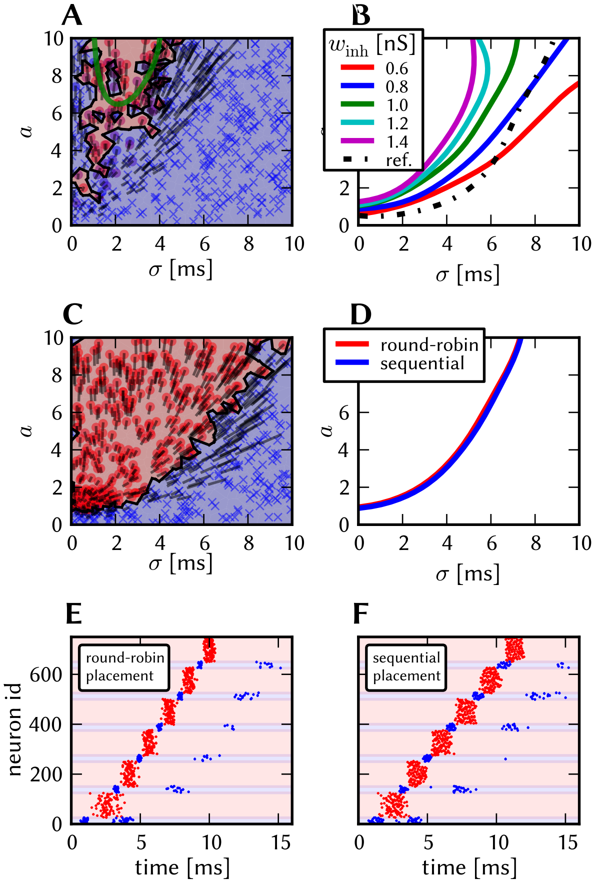

Figure S3.4. Additional simulations of the feed-forward synfire chain on the ESS without synapse loss: (**A**) (σ,a) state space on the ESS with default parameters and 20% weight noise. (**B**) After compensation of for all distortion mechanisms, different separatrices are possible by setting different values of the inhibitory weight. (**C**) Compensated state space belonging to the blue separatrix in **B**. w refers to the synaptic weight of local inhibition. (**D-F**) Investigation of effects of spike loss and jitter by using two different approaches for neuron placement. (**D**) Separatrices for round-robin and sequential neuron placement with parameters as for the green curve in **B**. Raster plots for round-robin (**E**) and sequential (**F**) neuron placement. Stimulus parameters: $a_0 = 1$ and $\sigma_0 = 1\,\mathrm{ms}$.

the second. In both setups, the utilized hardware and the number of neurons per routing bus was equal, allowing a fair competition between both. The separatrices for the two different placement strategies with otherwise identical parameters are virtually indistinguishable (Figure S3.4 **D**). Nevertheless, the raster plots (Figure S3.4 **E** and **F**) reveal the effect of the introduced jitter: For sequential placement, the spread of spike times within a group is roughly double than for round-robin placement and also the onset of the volley in the last group comes 1.5 ms later. In contrast to the reference simulation (cf. Figure S3.1), the fixed point of succesful propagation is not (0.12 ms,1) but (0.21 ms,1) for round-robin and (0.36 ms,1) for sequential placement.

We conclude that, especially for dense pulses, the subsequent processing of spikes in the hardware

leads to a temporal spread of the pulse volley, which however has virtually no influence on the filter properties of the synfire chain.
