# Supplementary material for: Characterization and Compensation of Network-Level Anomalies in Mixed-Signal Neuromorphic Modeling Platforms
Source: PLoS One. 2014 Oct 10;9(10):e108590. doi: 10.1371/journal.pone.0108590 (PMC4193761; doi:10.1371/journal.pone.0108590)
Supplement: Appendix S4 — Self-sustained asynchronous irregular activity. Methods, model parameters, and further simulations of the AI network. (PDF) [file pone.0108590.s004.pdf]

Appendix S4 Self-sustained asynchronous irregular activity

S4.1 Network simulation setup

The default model consists of 3920 neurons (80 % pyramidal and 20 % inhibitory) equally distributed on a two-dimensional lattice of 1×1 mm^2 folded to a torus. The connection probability is distance-dependent and is normalized such that each neuron receives synaptic input from 200 excitatory and 50 inhibitory neurons. All simulations run for 10 s. 2 % of all neurons in the network are initially stimulated by one individual Poisson source for 100 ms in order to induce initial network activity. The default size was chosen such that the model can be fully realized on the BrainScaleS hardware without losing any synaptic connections in the mapping step (Section 2.2), thereby allowing us to compare topologically equivalent software simulations, with the only remaining difference lying in the non-configurable delays and dynamic constraints on the ESS.

S4.1.1 Model parameters

The neuron parameters of the AdEx model used in this benchmark are listed in Table S4.1 and are equal to those in [88] with the only difference being that excitatory pyramidal cells have neuronal spike-triggered adaptation while inhibitory cells do not. Sweeps are performed over the two-dimensional (g_{exc}, g_{inh}) parameter space, with the ranges being 3 nS to 11 nS for g_{exc} and 50 nS to 130 nS for g_{inh}. The Poisson sources for the initial network stimulation have a mean rate of 100 Hz and project onto the network's neurons with a synaptic weight of 100 nS. The distance-dependent connection probability has a Gaussian profile with a spatial width of $\sigma = 0.2$ mm. Synaptic delays depend on the distance according to the following equation: $t_{\mathrm{delay}} = 0.3\,\mathrm{ms} + \frac{d}{v_{\mathrm{prop}}}$, with d being the distance between two cells and $v_{\mathrm{prop}} = 0.2\,\mathrm{mm\,ms^{-1}}$ the spike propagation velocity. The distribution of delays is shown in Figure S4.1, the average delay in the network amounts to 1.55 ms.

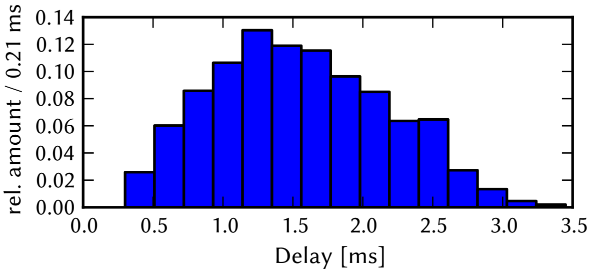

Figure S4.1. Histogram of delays in the AI network. The mean delay is 1.55 ms.

S4.1.2 Network scaling

When the network is scaled up in size, we only increase the number of neurons while keeping the number of afferent synapses per neuron constant. All other parameters concerning the connectivity do not change, including the size of the cortical sheet, the distance-dependent delays and connection probability, as well as the ratio of excitatory to inhibitory cells. Neuron and synapse parameters remain unaltered.

Table S4.1. AdEx Neuron parameters used in the AI network

Parameter	Pyramidal	Inhibitory	Unit
C_m	0.25	0.25	nF
τ_refrac	5	5	ms
E^spike	-40	-40	mV
E^r	-70	-70	mV
E_L	-70	-70	mV
τ_m	15	15	ms
a	1	1	nS
b	0.005	0	nA
Δ_T	2.5	2.5	mV
τ_w	600	600	ms
E_T	-50	-50	mV
$E^\mathrm{rev,e}$	0	0	mV
$E^\mathrm{rev,i}$	-80	-80	mV
$\tau^\mathrm{syn,e}$	5	5	ms
$\tau^\mathrm{syn,i}$	5	5	ms

S4.2 Functionality criteria

The survival time is defined as the last spike time in the network. If the network survives until the end of the simulation, we consider it as self-sustaining. Additionally, several criteria are employed to characterize the network's activity, regularity and synchrony.

The mean firing rate of all pyramidal neurons is used to classify the overall activity of the network. The variance of the firing rates across the pyramidal neurons measures the homogeneity of their response. For a better comparison, we look at the relative variance, i.e., the coefficient of variation of the firing rates $\mathrm{CV}_\mathrm{rate} = \frac{\sigma(\nu)}{\overline{\nu}}$, where $\overline{\nu}$ and $\sigma(\nu)$ are the mean and standard deviation of the average firing rates ν of the inidividual neurons.

The coefficient of variation of interspike intervals (CV_ISI) serves as an indicator of spiking regularity. It is calculated via

$$\mathrm{CV}_\mathrm{ISI} = \frac{1}{N}\sum_{i=1}^{N}\frac{\sigma_i(\mathrm{ISI})}{\overline{\mathrm{ISI}_i}} \tag{S4.1}$$

$\sigma_i(\mathrm{ISI})$ is the standard deviation of interspike intervals in the i-th spike train, while $\overline{\mathrm{ISI}}_i$ is the mean interspike interval in the same spike train. N is the number of averaged spike trains which is set to the number of pyramidal cells for each simulation. CV_ISI is 0 for a regular spike train and approaches 1 for a sufficiently long Poisson spike train.

The correlation coefficient CC is defined via

$$\mathrm{CC} = \frac{1}{P}\sum_{j,k}^{P}\frac{\mathrm{Cov}(S_j, S_k)}{\sigma(S_j)\sigma(S_k)} \tag{S4.2}$$

The sum runs over $P = 5000$ randomly chosen pairs of spike trains (j, k) from the excitatory population. S_i is the time-binned spike count in the i-th spike train with a bin width of $\Delta = 5\,\mathrm{ms}$. $\sigma(S_i)$ denotes the

standard deviation of S_i, and $\mathrm{Cov}(S_j, S_k)$ the covariance of S_j and S_k. CC approaches 0 for sufficiently long independent spike trains and is 1 for linearly dependent (S_j, S_k). The simulation results were cross-checked with a bin width of $\Delta = 2\,\mathrm{ms}$.

The power spectrum $S(\omega)$ of a spike train is calculated via

$$A_k = \sum_{m=0}^{N-1} r_m \exp\left(-2\pi i \frac{mk}{N}\right) \qquad k = 0, \ldots, N-1 \tag{S4.3}$$

$$\omega_k := \frac{2\pi k}{N\Delta} \tag{S4.4}$$

$$S(\omega_k) := |A_k|^2 N\Delta \tag{S4.5}$$

using the time-binned population firing rate r_i with $i \in \{0, ..., N-1\}$ with a bin width of Δ for a spike train of length $N\Delta$ (see, e.g. 3.1.4 in [97]). For the AI network we used a bin width of $\Delta = 1\,\mathrm{ms}$ for calculating the raw power spectra, and a $\sigma = 5\,\mathrm{Hz}$ for the Gauss-filtered versions which where then used to determine the peak frequency (i.e. the first non-zero peak in the power spectrum).

In case of the L2/3 model, the power spectra were calculated from Gauss-filtered ($\sigma = 5\,\mathrm{ms}$) spike data with a bin width of $\Delta = 0.1\,\mathrm{ms}$ and (unless otherwise stated) smoothed with a $\sigma = 0.3\,\mathrm{ms}$ Gauss-filter.

For all statistics, the first second of the simulation is left out, i.e. only the 9 seconds from 1 s to 10 s are considered. If the network did not survive until the end of the simulation, the firing rate was calculated between 1 s and the survival time, or between 0.1 s and the survival time for the case when the latter was smaller than 1 s.

S4.3 Iterative compensation

In the so-called iterative compensation, we sequentially modify individual parameters such that the response of each neuron is modified to match its target response. In our case, we iteratively change the spike detection voltage E_T such that the firing rate of each neuron is shifted towards the target rate. At each step, the threshold voltage is adapted as follows:

$$E_\mathrm{T}^{\mathrm{n+1,i}} = E_\mathrm{T}^{\mathrm{n,i}} + (\nu^{\mathrm{tgt}} - \nu^{\mathrm{n,i}}) c_{\mathrm{comp}} \tag{S4.6}$$

where $E_\mathrm{T}^{\mathrm{n,i}}$ and $\nu^{\mathrm{n,i}}$ are the threshold voltage and firing rate of neuron i of the n-th step, ν^{tgt} is the target rate for all neurons of a population and c_{comp} is a compensation factor that links the firing response and the threshold voltage. The target rate ν^{tgt} is computed separately for the excitatory and inhibitory population from the reference simulations (Section 3.3.2). We choose the compensation factor for each $(g_{\mathrm{exc}}, g_{\mathrm{inh}})$ state in the following manner: Similar to the mean-field approach in section 3.3.6, we consider the response rate of an excitatory neuron given a network firing rate of ν^{tgt}, that is, the neuron is stimulated by 200 excitatory and 50 inhibitory Poisson sources with rate ν^{tgt}. We then vary the threshold voltage of said neuron between $-54\,\mathrm{mV}$ and $-46\,\mathrm{mV}$ and thereby determine the dependency of the response rate on the threshold voltage. From a linear fit of this dependency, we extract the slope m, and set the compensation factor to $c_{\mathrm{comp}} = \frac{0.5}{m}$ (Figure S4.2). The factor of 0.5 was chosen to limit the change of the mean rate in each step in order to avoid oscillations in the compensation procedure. Whenever we changed the spike initiation voltage E_T, we shifted the spike detection voltage E^{spike} equally.

We remark that this compensation method requires the parameters for every individual neuron to be fine-tunable. This is the case for the BrainScaleS wafer-scale hardware, where the AdEx parameters of every hardware neuron are independently configurable with sufficient precision by means of analog floating gate memories (Section 2.1), in contrast to the synaptic weights which are restricted to a 4-bit

precision in typical operation mode.

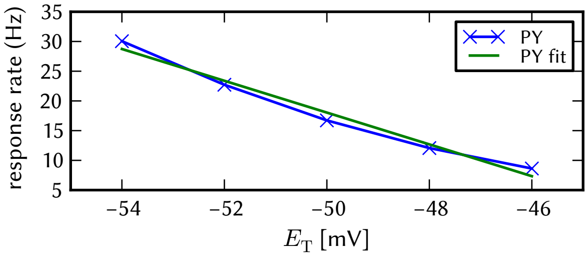

Figure S4.2. Example compensation factor assertion for the state ($g_\mathrm{exc} = 9\,\mathrm{nS}$, $g_\mathrm{inh} = 90\,\mathrm{nS}$) of the AI network: The Figure shows the response rate of an excitatory neuron stimulated by 200 excitatory and 50 inhibitory Poisson sources with rate 12.38 Hz depending on its spike initiation threshold E_T. The slope $m = -2.6745\,\frac{\mathrm{Hz}}{\mathrm{mV}}$ of the linear fit is then used to calculate the compensation factor $c_\mathrm{comp} = \frac{0.5}{m} = -0.18695\,\frac{\mathrm{mV}}{\mathrm{Hz}}$.

S4.4 Further simulations

S4.4.1 Network size scaling behavior

To investigate what happens when the network is scaled according to the rules given in section S4.1.2, we pick one state of the (g_exc, g_inh) space and vary the network size between 5000 and 50 000 neurons. The results for the (9 nS, 90 nS) state can be seen in Figure S4.3: The mean firing rate slightly increases with size until its saturates, while the variance of the firing rate across neurons remains approximately constant (**A**). Like the firing rate, the irregularity (CV_ISI) increases and saturates with size (**B**). The synchronicity (CC) decreases with size, as one would expect (**C**). The power spectrum of global activity exhibits the same profile for all sizes, however the power is scaled inversely to the network size (**D** and **E**).

S4.4.2 Non-configurable axonal delays

In section 3.3.3 we argue that non-configurable delays on the BrainScaleS hardware only have a minimal effect on the AI network because the average delay in the model matches the estimated average delay on the hardware. Here, we provide the simulation results and further investigations on the influence of the delay on the network dynamics. For the analysis of the effects of non-configurable delay we repeated the (g_exc, g_inh) sweep with all synaptic delays set to 1.5 ms, cf. section 2.4. This distortion mechanism only affected the power spectrum of global actitivity but not the other criteria such that we show only the peak frequency parameter spaces in Figure S4.4. The distorted network (**B**) with a constant delay of 1.5 ms is not significantly different from the default network with distance-dependent delays (**A**), both the region of sustained activity and the position of the peak in the power spectrum are in good match. The same holds for ESS simulations (**C**), where non-configurable delays were the only active distortion mechanism.

To further investigate the influence of the delays, we ran additional simulations where all delays in the network were set to 0.1 ms (**D**), and 3 ms (**E**), respectively. Lowering delays increases the speed of activity propagation such that the position of the peak in the power spectrum is shifted towards higher frequencies. For higher delays the peak frequency decreases analogously, but also the region of sustained activity diminishes significantly. (**F**) shows simulations with distance-dependent delays scaled by a factor

of 2 with respect to the baseline model, thus having an average delay of 3.1 ms (cf. Figure S4.1). While the peak frequency is in good agreement with the 3 ms simulations, the region of sustained states is extended and even larger than in the baseline setup. Herewith our simulations affirm that distance-dependent delays in fact do expand the region of self-sustained states in the (g_{exc}, g_{inh}) space (cf. section 3.3.1).

S4.4.3 Combining distortion mechanisms: synapse loss and synaptic weight noise

We also investigate what happens when both synapse loss and synaptic weight noise are active at the same time. Additionally, we test up to which extent we can compensate for both sources of distortions. To do so we scaled both mechanisms up to 90 % and tried to restore the original behavior for two states: (9 nS, 90 nS) and (10 nS, 70 nS).

The relative change of the mean rate and $\mathrm{CV}_{\mathrm{rate}}$ are shown in Figure S4.5 for the (9 nS, 90 nS) state. For this state, synapse loss compensation works fine up to a level of 50 %: the relative change of the mean rate and $\mathrm{CV}_{\mathrm{rate}}$ are close to 0. The compensation fails for synapse losses of 70 % and above: when the original firing rate is recovered, the network is unstable, i.e. it does not survive until the end of the experiment. The amount of synaptic weight noise has no effect on this behavior. We remark that, during the iterative compensation, there are stable networks with a slightly higher firing rate than the target rate: the network becomes unstable when approaching its target rate. This is in accordance with observations from the 50 % loss parameter space compensation in Figure 21, where the region of sustained activity is smaller than before, i.e. requiring a higher frequency for fewer synapses. We also note that our iterative compensation algorithm does not recover distorted networks that die out shortly after initial stimulation (cf. the 90 % synapse loss column in Figure S4.5 **A**). Synaptic weight noise does not pose a problem to the iterative compensation: In all cases the mean rate could be fully recovered and the variance of firing rates close to the original level, with the relative difference of $\mathrm{CV}_{\mathrm{rate}}$ being smaller than 1.5.

For the (10 nS, 70 nS) state, compensation was capable of restoring a synapse loss including 70 %, cf. Figure S4.6. Interestingly, the reduction of the variation of firing rates after 10 compensation steps performed slightly better when starting with a higher synapse loss.

We summarize that the iterative method effectively compensates the distortions induced by synapse loss combined with synaptic weight noise, at least when the synapse loss does not exceed 50 %. Furthermore, we expect these results to hold also for a large area in the (g_{exc}, g_{inh}) space where the network is in the asynchronous regime.

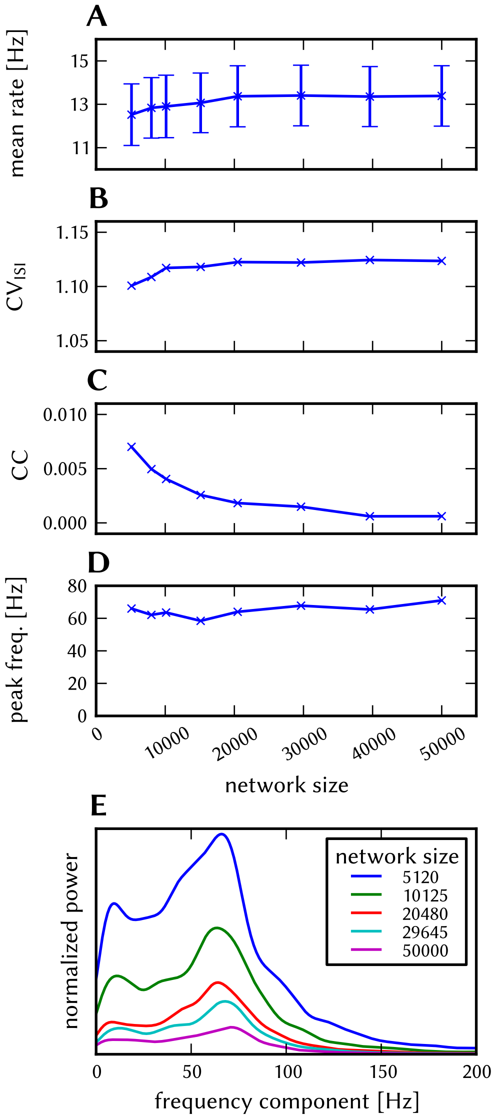

Figure S4.3. Network size scaling behavior of the AI network for the (9 nS, 90 nS) state. Mean and variance of firing rate across the PY neurons (**A**), coefficient of variance of inter-spike intervals (**B**), coefficient of pairwise cross-correlation (**C**), and power spectrum of global activity: full spectra (**D**) and peak frequencies (**E**).

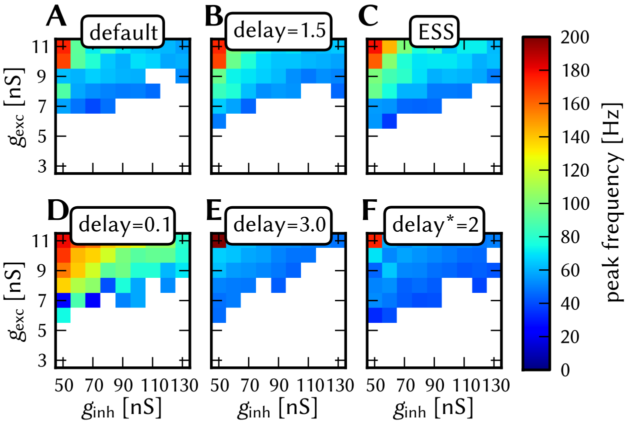

Figure S4.4. Effects of axonal delays on the AI network. (g_{exc}, g_{inh}) spaces with the peak frequency of the global pyramidal activity for different axonal delay setups: default with distance-dependent delays (**A**), constant delay of 1.5 ms (**B**), simulation on the ESS where delay is not configurable (**C**), constant delay of 0.1 ms (**D**), constant delay of 3.0 ms (**E**), distance-dependent delays scaled by factor of 2 with respect to default setup (**F**).

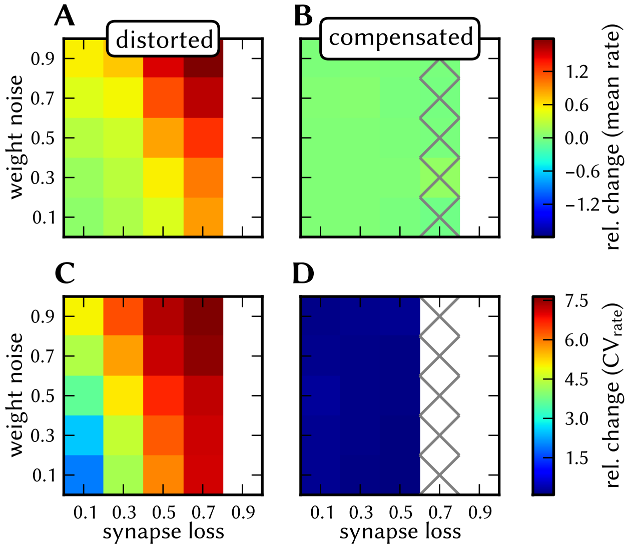

Figure S4.5. Compensation for combined distortion mechanisms in the AI network with the iterative method. Sweep over synapse loss and synaptic weight noise for the ($g_{\mathrm{exc}} = 9\,\mathrm{nS}$, $g_{\mathrm{inh}} = 90\,\mathrm{nS}$) state. Relative change of the firing rate with respect to the undistorted network for distorted (**A**) and compensated (**B**) simulations. Relative change of $\mathrm{CV}_{\mathrm{rate}}$ with respect to the undistorted network for distorted (**C**) and compensated (**D**) simulations. The compensated simulations refer to the 10th step of iterative compensation. White data points stand for networks where the distorted network did not survive. Data points marked with a cross denote cases where the compensated network did not survive.

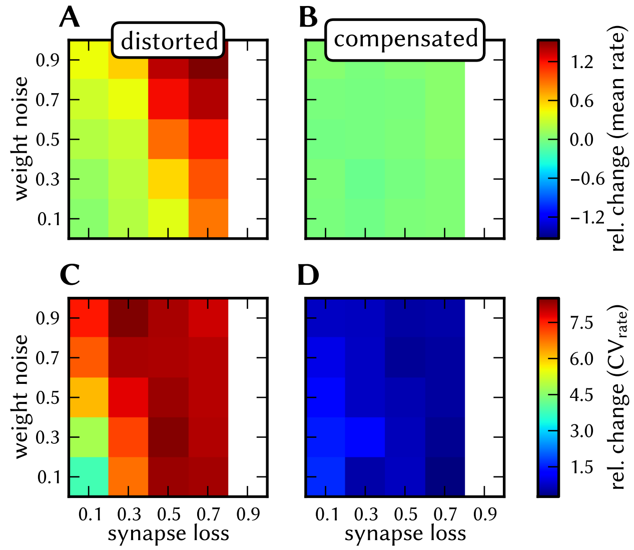

Figure S4.6. Compensation for combined distortion mechanisms in the AI network with the iterative method. Sweep over synapse loss and synaptic weight noise for the ($g_{\mathrm{exc}} = 10\,\mathrm{nS}$, $g_{\mathrm{inh}} = 70\,\mathrm{nS}$) state. Relative change of the firing rate with respect to the undistorted network for distorted (**A**) and compensated (**B**) simulations. Relative change of $\mathrm{CV}_{\mathrm{rate}}$ with respect to the undistorted network for distorted (**C**) and compensated (**D**) simulations. The compensated simulations refer to the 10th step of iterative compensation. White data points stand for cases where the distorted network did not survive.
